# Xenacoelomorph Neuropeptidomes Reveal a Major Expansion of Neuropeptide Systems during Early Bilaterian Evolution

**DOI:** 10.1093/molbev/msy160

**Published:** 2018-08-24

**Authors:** Daniel Thiel, Mirita Franz-Wachtel, Felipe Aguilera, Andreas Hejnol

**Affiliations:** 1 Sars International Centre for Marine Molecular Biology, University of Bergen, Bergen, Norway; 2 Proteome Center Tübingen, University of Tübingen, Tübingen, Germany; 3 Departamento de Bioquímica y Biología Molecular, Facultad de Ciencias Biológicas, Universidad de Concepción, Concepción, Chile

**Keywords:** neuropeptides, G protein-coupled receptors, Xenacoelomorpha, Cnidaria, CNS evolution, Bilateria

## Abstract

Neuropeptides are neurosecretory signaling molecules in protostomes and deuterostomes (together Nephrozoa). Little, however, is known about the neuropeptide complement of the sister group of Nephrozoa, the Xenacoelomorpha, which together form the Bilateria. Because members of the xenacoelomorph clades *Xenoturbella*, Nemertodermatida, and Acoela differ extensively in their central nervous system anatomy, the reconstruction of the xenacoelomorph and bilaterian neuropeptide complements may provide insights into the relationship between nervous system evolution and peptidergic signaling. Here, we analyzed transcriptomes of seven acoels, four nemertodermatids, and two *Xenoturbella* species using motif searches, similarity searches, mass spectrometry and phylogenetic analyses to characterize neuropeptide precursors and neuropeptide receptors. Our comparison of these repertoires with previously reported nephrozoan and cnidarian sequences shows that the majority of annotated neuropeptide GPCRs in cnidarians are not orthologs of specific bilaterian neuropeptide receptors, which suggests that most of the bilaterian neuropeptide systems evolved after the cnidarian–bilaterian evolutionary split. This expansion of more than 20 peptidergic systems in the stem leading to the Bilateria predates the evolution of complex nephrozoan organs and nervous system architectures. From this ancient set of neuropeptides, acoels show frequent losses that correlate with their divergent central nervous system anatomy. We furthermore detected the emergence of novel neuropeptides in xenacoelomorphs and their expansion along the nemertodermatid and acoel lineages, the two clades that evolved nervous system condensations. Together, our study provides fundamental insights into the early evolution of the bilaterian peptidergic systems, which will guide future functional and comparative studies of bilaterian nervous systems.

## Introduction

**Fig. 1.**
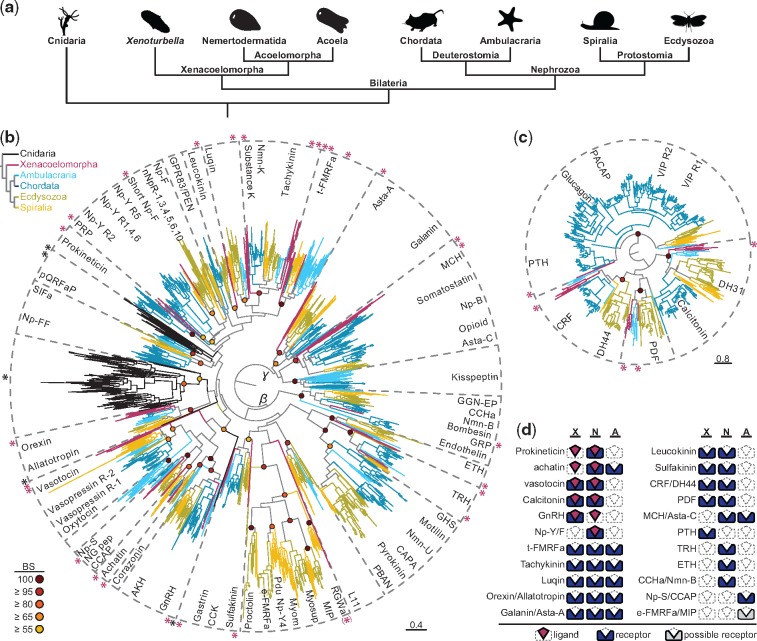
Phylogenetic analysis of neuropeptide GPCRs. (*a*) Simplified phylogeny of Bilateria with Cnidaria as their sister group. (*b*) RAxML analysis of rhodopsin type neuropeptide GPCRs. Rhodopsin *beta* GPCRs are rooted against rhodopsin *gamma* GPCRs. Bootstrap values of the particular nodes are represented as red-to-yellow circles as shown on the lower left. The scale bar on the lower right indicates amino acids substitution rate per site. Magenta asterisks indicate the position of xenacoelomorph sequences, and black asterisks indicate the position of cnidarian sequences. If more than one monophyletic cluster of xenacoelomorph sequences is present within one receptor type, it is indicated by the corresponding number of asterisks. Dashed lines demarcate orthologous receptor types of different animal groups. Color coding of tree branches is shown on the upper left: Magenta = xenacoelomorphs, blue = chordates, light blue = ambulacrarians, olive = ecdysozoans, yellow = spiralians, black = cnidarians. (*c*) RAxML analysis of secretin-type neuropeptide GPCRs. Bootstrap support, substitution rate per site, and taxa color coding are depicted as in figure (*b*). (*d*) Distribution of conserved bilaterian neuropeptide receptors and ligands in Xenacoelomorpha. Blue boxes show the presence of conserved receptors, whereas the gray boxes display the presence of a possible conserved receptor (boot strap support < 50%). Magenta diamonds show presence of conserved ligand. A, Acoela; a, amide; AKH, adipokinetic hormone; Asta, allatostatin; BS, bootstrap support; CCAP, crustacean cardioaccelatory peptide; CCK, cholecystokinin; CRF, corticotropin releasing factor; DH, diuretic hormone; GGN-EP, GGN excitatory peptide; e-, ecdysozoan; ETH, ecdysis triggering hormone; GHS, growth hormone segretagogue; GnRH, gonadotropin releasing hormone; GRP, gastrin releasing peptide; N, Nemertodermatida; Np, neuropeptide; nNpR, nematode neuropeptide receptor; Nmn, neuromedin; L11 = elevenin; MCH, melanin concentrating hormone; MIP, myoinhibitory peptide; Myosup, myosuppressin; PACAP, pituitary adenylate cyclase-activating polypeptide; PBAN, pheromone biosynthesis activating neuropeptide; PDF, pigment dispersing factor; Pdu, *Platynereis dumerilii*; NG pep, NG peptide; pQRFaP, pyroglutaminated RFamide peptide; PTH, parathyroid hormone; PRP, prolactin releasing peptide; R, receptor; t-, trochozoan; TRH, thryotropin releasing hormone; VIP, vasoactive intestinal peptide; X, *Xenoturbella*. Silhouette pictures are downloaded from www.phylopic.org under the Public Domain Dedication 1.0 (CC0 1.0) or Public Domain Mark 1.0 license without any copyright restrictions or were drawn by one of the authors.

Neuropeptides are a diverse group of signaling molecules that play a crucial role in the function of the nervous system in most metazoan animals (Hökfelt et al. 2000; Grimmelikhuijzen et al. 2002; Jékely 2013; Mirabeau and Joly 2013; Roch and Sherwood 2014). These signaling molecules do not only mediate by direct synaptic transmission but mostly transfer signals by volume transmission as neurohormones or neuromodulators and, thus, play important roles in the modulation of neural circuits (Christie et al. 1995; Nassel and Winther 2010; Catak et al. 2014; Diao et al. 2017; Senatore et al. 2017; Williams et al. 2017; Jékely et al. 2018). Most neuropeptides are about 3–20 amino acids long and transfer signals via conserved G protein-coupled receptors (GPCRs) (Frooninckx et al. 2012; Jékely 2013; Mirabeau and Joly 2013; Stevens et al. 2013). Comparison of neuropeptides and neuropeptide GPCRs of different animals has shown that the last common ancestor of the Deuterostomia and Protostomia (together Nephrozoa, fig. 1*a*) had at least 30 different peptidergic systems (Janssen et al. 2008; Grimmelikhuijzen and Hauser 2012; Jékely 2013; Mirabeau and Joly 2013; Semmens et al. 2015; Tian et al. 2016; Van Sinay et al. 2017; Zandawala et al. 2017; Elphick et al. 2018). These peptidergic systems are involved in various physiological and behavioral processes such as osmoregulation and water balance (Salzet et al. 1994; Fujino et al. 1999; De Mota et al. 2004), muscle activity (Semmens et al. 2013; Dickinson et al. 2015), metabolism and growth (Mizoguchi and Okamoto 2013; Van Sinay et al. 2017), feeding and defecation (Sakurai et al. 1998; Wang et al. 2013; Williams et al. 2015; Chung et al. 2017), reproduction (Lindemans et al. 2009; Beets et al. 2013), and stress tolerance (Schank et al. 2012; Terhzaz et al. 2015; Cannell et al. 2016). In contrast to the large number of plesiomorphic peptidergic systems of nephrozoans, the only peptidergic systems that are shared between Nephrozoa and Cnidaria are insulin-like peptides (ILPs), glycoprotein hormone (GPH)-related peptides, and prokineticin-related peptides (Jékely 2013; Roch and Sherwood 2014; Elphick et al. 2018). The existence of a few putative cnidarian orthologs of nephrozoan neuropeptides or neuropeptide GPCRs has been proposed, but their actual phylogenetic relationship is inconclusive (Anctil 2009; Alzugaray et al. 2013; Jékely 2013; Krishnan and Schioth 2015; Alzugaray et al. 2016). Therefore, the available data suggest that the major radiation of the nephrozoan peptidergic systems occurred sometime after the cnidarian–bilaterian evolutionary split (Jékely 2013; Elphick et al. 2018). Compared with cnidarians, nephrozoans have several evolutionary novelties, such as a coelom, a through gut, an excretory system, and a circulatory system, which need to be controlled by defined neural circuits (Holzer and Guth 1991; Zhang et al. 2014; Halberg et al. 2015; Cropper et al. 2018), and importantly, nephrozoans typically have highly condensed centralized nervous systems (CNS) (Hejnol and Lowe 2015). Many of the nephrozoan neuropeptides are also expressed in brains. Thus, orthologous neuropeptides have been used to compare brains and other parts of the nervous system between different animal species (Tessmar-Raible et al. 2007; Conzelmann and Jékely 2012; Tosches and Arendt 2013; Arendt et al. 2016; Kerbl et al. 2017). This led to the hypothesis that the origin of the bilaterian peptidergic systems is tightly connected to the origin of the different nephrozoan organ systems, such as their complex, condensed CNS (Jékely 2013). Such a hypothesis, however, has been difficult to test.

An important animal group for understanding the evolution of nervous systems is the Xenacoelomorpha. Recent phylogenomic evidence supports an earlier hypothesis that places the Xenacoelomorpha as the sistergroup to the Nephrozoa (Ruiz-Trillo et al. 1999; Hejnol et al. 2009; Srivastava et al. 2014; Cannon et al. 2016; Rouse et al. 2016). This placement has been challenged in a different phylogenomic analysis (Philippe et al. 2011) that places this clade as a sistergroup to Ambulacraria instead (see Discussion). Xenacoelomorphs lack several nephrozoan features, including coeloms, excretory organs, a circulatory system, and a through gut, but share their bilateral symmetry with clearly defined body-axes and the mesodermal germ layer as synapomorphic characters with nephrozoans (Jondelius et al. 2011; Hejnol 2015a, 2015b; Haszprunar 2016; Hejnol and Pang 2016). Furthermore, xenacoelomorphs display highly diverse neuroanatomies and seem to have evolved clade-specific CNS with multiple nerve cords and brain-like structures, which have evolved convergently to those of other bilaterians (Achatz and Martinez 2012; Hejnol 2015a; Perea-Atienza et al. 2015; Gavilan et al. 2016; Haszprunar 2016; Hejnol and Pang 2016; Raikova et al. 2016; Martin-Duran et al. 2018). Their divergent neuroanatomies provide a unique case to investigate general mechanisms regarding the early nervous system evolution in bilaterians. Xenacoelomorpha comprises three major clades: *Xenoturbella*, Nemertodermatida, and Acoela, with the latter two forming the clade Acoelomorpha (fig. 1*a*). The nervous system of *Xenoturbella* species is often considered to reflect a more ancestral form, as it only consists of a basiepidermal—somewhat cnidarian-like—nerve net without any considerable condensations (Raikova et al. 2000a; Hejnol and Rentzsch 2015; Stach 2015; Gavilan et al. 2016; Haszprunar 2016; Hejnol and Pang 2016). Nemertodermatids possess additional condensed basiepidermal nerve cords that can be located at different places along the dorsoventral axis, and additional basiepidermal anterior brain-like condensations are observed in many species (Raikova et al. 2004, 2016; Børve and Hejnol 2014; Martínez et al. 2017). The nervous system of acoels is considered as more derived with several novelties, including internalized anterior brains and multiple subepidermal pairs of longitudinal nerve cords (Achatz and Martinez 2012; Hejnol 2015a; Gavilan et al. 2016; Hejnol and Pang 2016; Raikova et al. 2016; Martínez et al. 2017). Immunohistochemical studies have demonstrated reactivity of antibodies that were raised against neuropeptides of nephrozoan animals, like different RFamides and SALMFamides (Raikova et al. 2000a, 2004; Reuter et al. 2001; Stach et al. 2005; Kotikova and Raiikova 2008; Semmler et al. 2010; Achatz and Martinez 2012; Børve and Hejnol 2014; Dittmann et al. 2018). However, not much is known about the actual xenacoelomorph neuropeptide repertoire, except for the presence of GPCRs that are related to FMRFamide, luqin, tachykinin, and neuropeptide F receptors (Thiel et al. 2017).

Due to the phylogenetic position of Xenacoelomorpha, investigations on these animals can be informative for the reconstruction of the evolutionary origin of nephrozoan peptidergic systems, with the potential to provide a deeper understanding of the connection between the emergence of neural morphological novelties and changes in peptidergic systems. Here, we conducted a detailed bioinformatic survey for neuropeptides and neuropeptide GPCRs in transcriptomes of 13 xenacoelomorph species of varying relatedness. Our in silico approach included a survey for neuropeptide precursors using sequence similarity and sequence motif searches that were complemented by a mass spectrometric analysis of peptide extracts from three acoel species. This nested survey allowed not only comparisons between different xenacoelomorph neuropeptide complements but also comparisons with other bilaterians and cnidarians. Together, we provide novel insights into the early diversification of bilaterian neuropeptide signaling systems and the evolution of peptidergic signaling in Xenacoelomorpha.

## Results

**Fig. 2.**
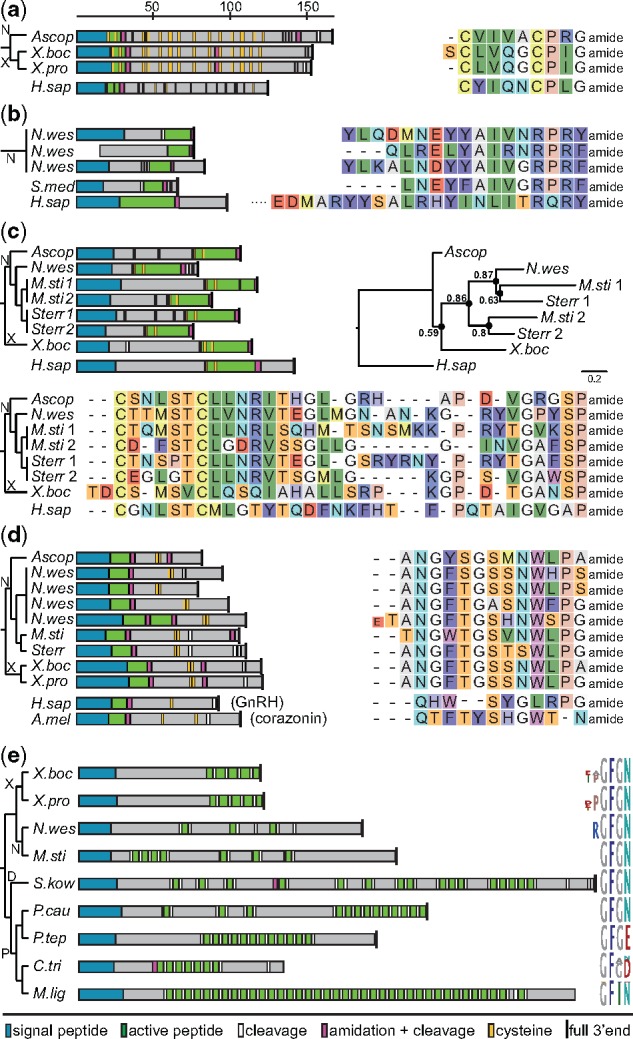
Xenacoelomorph orthologs of bilaterian neuropeptides. (*a*) Vasotocin-neurophysin precursor schemes and vasotocin sequences. (*b*) Neuropeptide Y/F precursor diagrams and active peptide sequences. (*c*) Calcitonin precursor schemes, phylogenetic FastTree analysis of predicted active ligands and alignment of predicted active peptides. (*d*) GnRH/corazonin related peptide precursor diagrams and predicted active ligands. (*e*) Achatin precursor schemes and peptide sequence logo of xenacoelomorph and nephrozoan sequences. D, Deuterostomia; N, Nemertodermatida; P, Protostomia; X, *Xenoturbella*; *A.mel*, *Apis mellifera*; *Ascop*, *Ascoparia sp.*; *C.tri*, *Charonia tritonis*; *H.sap*, *Homo sapiens*; *M.lig*, *Macrostomum lignano*; *M.sti*, *Meara stichopi*; *N.wes*, *Nemertoderma westbladi*; *P.tep*, *Parasteatoda tepidariorum*; *P.cau*, *Priapulus caudatus*; *S.kow*, *Saccoglossus kowalevskii*; *S.med*, *Schmidtea mediterranea*; *Sterr*, *Sterreria sp.*; *X.boc*, *Xenoturbella bocki*; *X.pro*, *Xenoturbella profunda.* Scale bar on the top indicates length of neuropeptide precursors in number of amino acids. Color coding of amino acid residues of the sequences is according to their biochemical properties (i.e., Rasmol coloring).

**Fig. 3.**
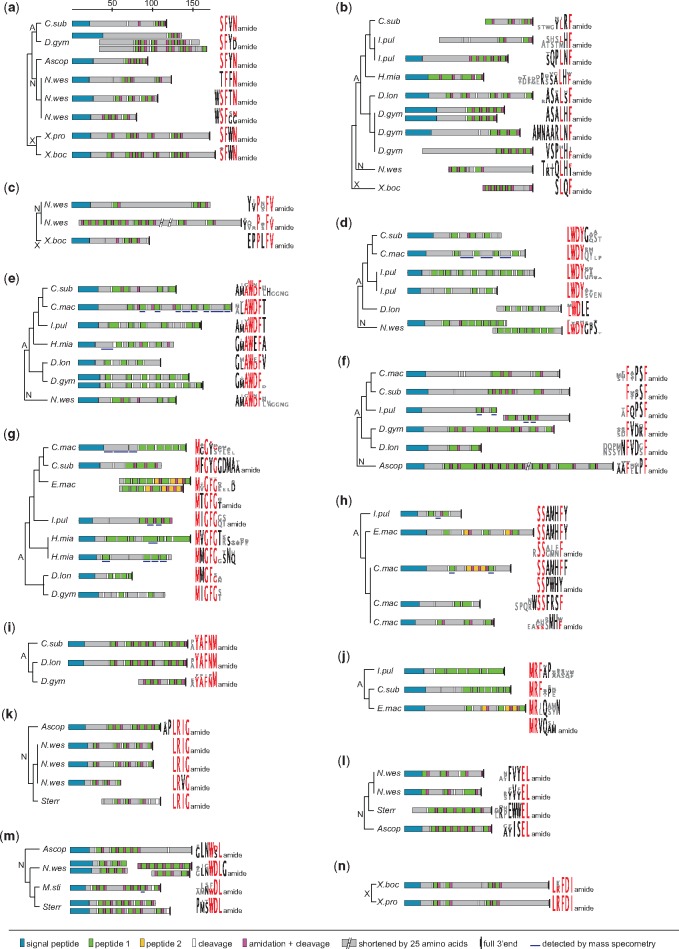
Neuropeptide precursors and their peptide sequence logo representations of xenacoelomorph-specific MCPs. (*a*) SFxNamides. (*b*) LxFamides. (*c*) PxFVamides. (*d*) LWDY peptides. (*e*) AWDF peptides. (*f*) FxxxFamides. (*g*) MxGFG peptides. (*h*) SSxxxF peptides. (*i*) YAFNMamides. (*j*) MRF peptides. (*k*) LRIGamides. (*l*) ELamides. (*m*) WDLamides. (*n*) LRFDIamides. Peptide sequence logo representations are created using all aligned peptide sequences of the corresponding precursor. Conserved amino acid residues between species are highlighted in red. A, Acoela; N, Nemertodermatida; X, *Xenoturbella*; *Ascop*, *Ascoparia sp.*; *C.sub*, *Childia submaculatum*; *C.mac*, *Convolutriloba macropyga*; *D.gym*, *Diopisthoporus gymnopharyngeus*; *D.lon*, *Diopisthoporus longitubus*; *E.mac*, *Eumecynostomum macrobursalium*; *H.mia*, *Hofstenia miamia*; *I.pul*, *Isodiametra pulchra*; *M.sti*, *Meara stichopi*; *N.wes*, *Nemertoderma westbladi*; *Sterr*, *Sterreria sp.*; *X.boc*, *Xenoturbella bocki*; *X.pro*, *Xenoturbella profunda*.

**Fig. 4.**
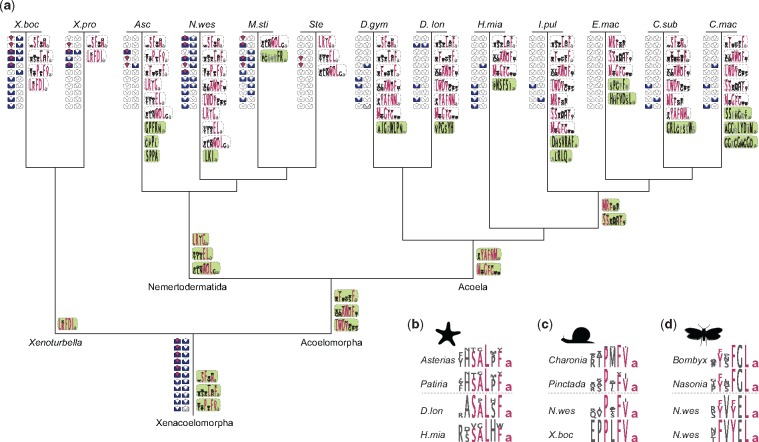
Distribution of peptidergic systems in Xenacoelomorpha and comparision of xenacoelomorph MCPs. (*a*) Distribution of detected ancestral bilaterian systems (left side of a branch) and novel MCPs (right side of a branch) in the 13 xenacoelomorph species. Symbols and order of bilaterian systems are similar to figure 1. Newly evolved MCPs are shown with a green background, next to the branch where they evolved. (*b*) Comparison of two xenacoelomorph LxFamides with echinoderm L-type SALMFamides of *Asterias rubens* and *Patiria miniata*. (*c*) Comparison of two xenacoelomorph PxFVamides with molluskan PxFVamides of *Charonia tritonis* and *Pinctada fucata*. (*d*) Comparison of two xenacoelomorph ELamides with insect allatostatin A of *Bombyx mori* and *Nasonia vitripennis*. Residues of peptide sequence logos that are conserved across species are highlighted in magenta.

We identified various types of peptidergic systems in xenacoelomorphs that have previously been reported from other animal lineages and found that most of the annotated cnidarian neuropeptide GPCRs are not orthologs of the proposed nephrozoan neuropeptide GPCRs. The three types of ancient metazoan peptidergic systems that have orthologs in nephrozoans as well as some nonbilaterians are also present in xenacoelomorphs (supplementary fig. 1, Supplementary Material online). Furthermore, we detected that 21 out of the 28 peptidergic systems that have previously been characterized in nephrozoans are present in xenacoelomorphs (figs. 1 and 2). In addition, we detected 14 types of multicopy peptides (MCPs) that are specific to all xenacoelomorphs or particular subclades (fig. 3) and several MCPs that seem specific to single xenacoelomorph species (supplementary fig. 2, Supplementary Material online). A comparison of the peptidergic complements of *Xenoturbella*, Nemertodermatida, and Acoela shows great differences in the representation of ancestral bilaterian systems and novel neuropeptides (fig. 4*a*; see supplementary tables 1 and 2, Supplementary Material online, for a detailed distribution), indicating multiple changes in the different xenacoelomorph clades.

### Most of the Acoel Species Lack Otherwise Conserved Metazoan Neuropeptides

The three types of neuropeptides that are conserved between nephrozoans and nonbilaterians, the GPH-related peptides, the ILPs and the prokineticin-related peptides, are also found in the xenacoelomorph lineages (supplementary fig. 1, Supplementary Material online). GPH alpha precursors are found in both *Xenoturbella* species (i.e., *X. bocki* and *X. profunda*) and in the nemertodermatid *Nemertoderma westbladi*. In both *Xenoturbella* species we also identified GPH beta, and in *X. bocki* the GPH-related bursicon beta precursor. In acoels, we detected a single sequence in *Isodiametra pulchra*, which shows no clear similarity with a specific type of GPH-related peptide in our phylogenetic analysis (supplementary fig. 1*a*, Supplementary Material online), but this protein sequence has the typical cysteine arrangement of a bursicon (see Roch and Sherwood 2014).

ILPs were found in the nemertodermatids *N. westbladi*, *Meara stichopi* and *Ascoparia* sp., as well as in both *Xenoturbella* species. The only identified acoel ILP was detected in the transcriptome of *Diopisthoporus gymnopharyngeus*, and this sequence shows an unusually long C-chain between the predicted bioactive A- and B-chains (supplementary fig. 1*b*, Supplementary Material online). Interestingly, the nemertodermatid *Ascoparia* sp. has a large expansion of 23 potential ILPs, compared with the one, two or three paralogs of the other xenacoelomorphs (supplementary fig. 1*b*, Supplementary Material online). Although many bilaterians possess more than one ILP, such an expansion is only known from a few species (Pierce et al. 2001; Mizoguchi and Okamoto 2013).

Prokineticin precursors were found in *X. bocki*, and in the nemertodermatids *M. stichopi* and *N. westbladi* (supplementary fig. 1*c*, Supplementary Material online). We could not detect a prokineticin-related peptide in any of the acoel transcriptomes. While receptors of GPH-related peptides and ILPs are also known to exist outside bilaterians (Steele et al. 1996; Jékely 2013; Roch and Sherwood 2014), prokineticin receptors are so far only known from deuterostomes (Jékely 2013; Mirabeau and Joly 2013). However, we identified an ortholog of the deuterostome prokineticin GPCR in the nemertodermatid *N. westbladi* (figs. 1*b* and *d*), which is the first evidence that the prokineticin GPCR already existed at the dawn of bilaterians.

### The Analysis of Cnidarian GPCRs Confirms That the Major Radiation of Peptidergic Systems Occurred after the Cnidarian–Bilaterian Evolutionary Split

While a previous large analysis of cnidarian and bilaterian taxa showed that only relaxin-like and GPH-related GPCRs are plesiomorphic to cnidarians and bilaterians (Jékely 2013), other smaller, but rather inconclusive studies, proposed the presence of additional cnidarian orthologs of bilaterian neuropeptide GPCRs (Anctil 2009; Alzugaray et al. 2013, 2016; Krishnan and Schioth 2015). In addition, various sequences deposited into NCBI database, which were predicted by the NCBI eukaryotic genome annotation pipeline as part of the genome projects from the cnidarians *Exaiptasia pallida* (Baumgarten et al. 2015), *Acropora digitifera* (Shinzato et al. 2011), and *Orbicella faveolata* (http://montastraea.psu.edu/genome/; last accessed August 23, 2018), were also annotated as bilaterian neuropeptide GPCRs. These neuropeptides include neuropeptide FF, neuropeptide Y, orexin, cholecystokinin-, qRFamide-, RYamide, substance-K-, bombesin-, gastrin-, galanin-, and somatostatin-GPCRs. In order to resolve the inconsistencies between previous studies and to better reconstruct the ancestral cnidarian–bilaterian neuropeptide complement, we reanalyzed the automatically annotated sequences, as well as GPCR sequences that were predicted from the genome of *Nematostella vectensis* as galanin-like, tachykinin/SIFamide-like, RFamide/neuropeptide FF/GnIH/neuropeptide Y-like, and GnRH/vasopressin-like receptors (Anctil 2009). Our analysis shows that most of these cnidarian GPCRs are closely related to each other, rather than to specific nephrozoan receptors (fig. 1*b* and supplementary figs. 3*a* and 4*a*, Supplementary Material online). Only the *Nema*. *vectensis* sequences showed some degree of diversity, with four to five types of GPCRs that clustered separately from each other (fig. 1*b* and supplementary figs. 3*a* and 4*a*, Supplementary Material online). One group of *Nema*. *vectensis* receptors showed similarity to the pQRFamide peptide receptors, while others had a more basal position to several different bilaterian GPCR types (fig. 1*b* and supplementary fig. 3*a*, Supplementary Material online). One of these basal sequences was originally predicted as a GnRH/vasotocin-like receptor by Anctil (2009). This sequence was also grouped in both phylogenetic analyses as a sister group to the “superclade” that consists of the bilaterian vasotocin, CCAP, GnRH, corazonin, and achatin GPCRs (fig. 1*b* and supplementary fig. 3*a*, Supplementary Material online). This result indicates that a single ancestral receptor might have been present in the last common ancestor of cnidarians and bilaterians, which later diversified along the stem leading to bilaterians into five different receptor types. The presence of several *Nema*. *vectensis* neuropeptide GPCR types also suggests that the last common ancestor of cnidarians and bilaterians possessed a few different neuropeptide GPCRs (four or five in our analysis, in addition to the relaxin-like and GPH-related GPCRs), while the main diversification into the different bilaterian GPCR groups occurred after the cnidarian–bilaterian evolutionary split, with a parallel diversification in the cnidarian lineage.

### Xenacoelomorph Orthologs of Nephrozoan Neuropeptide GPCRs Reveal the Early Diversification of Peptidergic Systems in Bilaterians

In our GPCR survey, we detected potential orthologs of 22 of the 29 previously characterized nephrozoan GPCR types, including the first nondeuterostome prokineticin GPCR (figs. 1*b*–*d*, and supplementary fig. 3, Supplementary Material online). Therefore, our analysis indicates the presence of these receptors in the last common ancestor of all bilaterians. Eighteen of these receptors were detected in nemertodermatids and 13 in *Xenoturbella* species, whereas only nine homologs were identified in Acoela (fig. 1*d* and supplementary table 1, Supplementary Material online). The different types of rhodopsin- and secretin-type GPCRs were confirmed in both the phylogenetic analysis (figs. 1*b* and *c*, and supplementary figs. 3*a* and *b*, Supplementary Material online) and the cluster analysis (supplementary figs. 4*a* and *b*, Supplementary Material online). We identified GPCRs related to trochozoan FMRFamide, tachykinin, luqin, allatotropin, and allatostatin A signaling in all three xenacoelomorph clades. Vasotocin, leukokinin, sulfakinin, calcitonin, DH44, and PDF type GPCRs were only found in *Xenoturbella* and Nemertodermatida. GPCRs that were only found in *Xenoturbella* but not in Acoelomorpha (Nemertodermatida + Acoela) were those related to GnRH and PTH signaling, whereas GPCRs related to achatin and allatostatin C signaling were found in both Acoelomorpha clades, but not in *Xenoturbella*. Receptors related to prokineticin, neuropeptide Y/F, TRH, ETH, and CCHamide signaling were restricted to Nemertodermatida. The only GPCRs exclusively detected in acoels were receptors that are related to CCAP signaling, and a single sequence that shows similarity to a large cluster that diversified greatly in protostomes into proctolin, arthropod FMRFamide, myomodulin, RGWamide, and myoinhibitory peptide receptors (fig. 1*b* and supplementary fig. 3*a*, Supplementary Material online). These results indicate an extensive loss of conserved bilaterian neuropeptide GPCRs in acoels. We did not include uncharacterized receptors in our survey (see Elphick et al. [2018] for more details about those receptors). The only receptor types that we could not identify in xenacoelomorphs, but that are known to be plesiomorphic to nephrozoans, are GPCRs related to corazonin, GPR83/PEN, neuromedin U/pyrokinin, neuropeptide FF/SIFamide, elevenin, pQRFamide peptide, and Kisspeptin signaling. The neuropeptide FF/SIFamide receptors and the pQRFamide receptors show in both phylogenetic analyses and in the cluster map an affinity to each other. The other missing receptors show no putative relationship, indicating that most receptor types without clear orthologs in xenacoelomorphs evolved as independent novelties in the nephrozoan lineage or were lost independently in the xenacoelomorph lineage. The pQRFamide receptors, however, show potential cnidarian orthologs (fig. 1*b*, supplementary figs. 3*a* and 4*a*, Supplementary Material online), which might indicate that this group of receptors could belong to the ancestral repertoire of bilaterian receptors and was lost in xenacoelomorphs.

### Conserved Bilaterian Neuropeptide Precursors Are Found in Nemertodermatida and *Xenoturbella*, but Not in Acoela

We identified neuropeptide precursors related to five nephrozoan neuropeptides: Vasotocin, neuropeptide Y/F, calcitonin, GnRH/corazonin, and achatin, which were only found in nemertodermatid and *Xenoturbella* species but not in any of the acoel transcriptomes (figs. 1*c* and 2). The presence of these neuropeptide precursors in xenacoelomorphs and the high similarity to their nephrozoan orthologs show that these ligands have diverged less when compared with many other bilaterian neuropeptides. Vasotocin–neurophysin precursors were found in both *Xenoturbella* species and in the nemertodermatid *Ascoparia* sp. (fig. 2*a*). Neuropeptide Y/F precursors were only found in the nemertodermatid *N. westbladi*, where we detected three potential paralogs (fig. 2*b*). Interestingly, the C-terminal YYAIVGRPRFamide motif of one of the *N. westbladi* paralogs is similar to the C-terminus of the neuropeptide Y of different protostome species (McVeigh et al. 2009; Veenstra 2010, 2011; Nassel and Wegener 2011; Conzelmann et al. 2013), which might be an indication for a similar ancestral neuropeptide Y/F. Calcitonin precursors were found in all four nemertodermatid species and in *X. bocki* (fig. 2*c*). The two more closely related species *M. stichopi* and *Sterreria* sp. possess two calcitonin paralogs that likely arose from a single duplication event (fig. 2*c*). GnRH/AKH/corazonin-like peptides were found in all four nemertodermatids and in both *Xenoturbella* species (fig. 2*d*). The predicted active ligands lack an N-terminal glutamine, which is characteristic for GnRH/CRZ-related peptides (Hansen et al. 2010; Lindemans et al. 2011; Hauser and Grimmelikhuijzen 2014; Roch et al. 2014; Li et al. 2016; Zandawala et al. 2017) and only absent in a few cases (Tian et al. 2016). It seems like one of the *N. westbladi* paralogs underwent a modification where the peptide precursor encodes two, instead of one ligand, which is typical for GnRH/AKH/CRZ peptides. Achatins were found in both *Xenoturbella* species and in the nemertodermatids *M. stichopi* and *N. westbladi* (fig. 2*e*). All predicted ligands share the GFGN sequence known from the achatin of the hemichordate *Saccoglossus kowalevskii*, the cephalochordate *Branchiostoma floridae*, and the ecdysozoans *Priapulus caudatus* and *Halicryptus spinulosus* (fig. 2*e* and supplementary precursor sequences, Supplementary Material online). The presence of identical GFGN sequences in deuterostome, protostome, and xenacoelomorph taxa could be due to a similar ancestral achatin.

### Acoelomorphs Show a Larger Expansion of Clade-Specific Novel Neuropeptides Compared with *Xenoturbella*

Beside the neuropeptides that are known from other animals, we identified 14 types of MCPs that seem to be specific to Xenacoelomorpha or particular xenacoelomorph clades (fig. 3). Three of these MCP types are symplesiomorphic for all Xenacoelomorpha (figs. 3*a*–*c* and 4*a*), three types seem to have emerged in the last common ancestor of acoelomorphs (figs. 3*d*–*f* and 4*a*), four types of MCPs are only shared among acoel species (figs. 3*g*–*j* and 4*a*), three types are only present in nemertodermatid species (figs. 3*k*–*m* and 4*a*), and one type of novel MCP seems to have appeared in the last common ancestor of *Xenoturbella* (figs. 3*n* and 4*a*). In addition, we found 11 full-length neuropeptide precursors that were only identified in single acoel species and five full-length precursors that were restricted to single nemertodermatid species (fig. 4*a* and supplementary fig. 2, Supplementary Material online).

#### Three Types of Novel MCPs Are Plesiomorphic to Xenacoelomorpha

The three types of ancestral xenacoelomorph MCPs are SFxNamides, LxFamides, and PxFVamides, with “x” standing for a variable amino acid position. SFxNamide peptides are 4–5 amino acids long and show a high conservation of the paracopies within each precursor (fig. 3*a*). LxFamide peptides show a higher variability between the paracopies of the precursors and partially great variability between the different species (fig. 3*b*). Some of the LxFamides show similarity to echinoderm L-type SALMFamide (fig. 4*b*). PxFVamides were only found in *N. westbladi* and *X. bocki* but were entirely absent in acoel species (fig. 3*c*). These PxFVamide peptides show similarity to neuropeptides with a similar motif that are known from mollusk species (fig. 4*c*).

#### Three Types of Novel MCP Are Plesiomorphic to Acoelomorpha

In acoel as well as nemertodermatid species, we discovered peptides that share the motifs AWDF, LWDY, and FxxxFamide (figs. 3*d*–*f*). The *Ascoparia* sp. FxxxFamide can also be grouped into the LxFamide peptides, as some of the paracopies possess a leucine in their third to last position (fig. 3*f*). In contrast to other xenacoelomorph peptides, most AWDF and LWDY orthologs have precursors on which the paracopies are very evenly distributed (figs. 3*d* and *e*). These two types of peptides also share a Trp-Asp sequence that is followed by an aromatic amino acid. This could be an indication that the AWDF and LWDY peptides might have evolved from a common ancestral sequence that split into two paralogs early in the stem leading to acoelomorphs.

#### Four Types of Novel MCPs Are Plesiomorphic to Acoela

Four types of MCPs are only present in acoel species. These peptides possess the motifs MxGFG(amide) (fig. 3*g*), SSxxxF(amide) (fig. 3*h*), YAFNMamide (fig. 3*i*), and MR(F) (fig. 3*j*). The MR(F), SSxxxF(amide), and MxGFG(amide) peptides are in some species amidated, in some species nonamidated, and in other species the precursor encodes both types. A mixture of otherwise structurally similar amidated and nonamidated peptides on the same precursor is rather uncommon for MCPs. The SSxxxFamides might be paralogs of the LxFamides, as they share several residues, particularly with the LxFamides of *D. longitubus*, *D. gymnopharyngeus* and *Hofstenia miamia*, indicating a possible duplication and diversification of an ancestral LxFamide peptide. The MxGFG peptides share the GFG motif with achatin peptides, but they do however vary between species and between paracopies in terms of length and amino acid sequence, whereas bilaterian achatins are usually tetrapeptides.

#### Three Types of Novel MCPs Are Plesiomorphic to Nemertodermatida

The three types of peptides that are present in several nemertodermatid species have the motifs LRIGamide (fig. 3*k*), ELamide (fig. 3*l*), and WDL(G)amide (fig. 3*m*). The *N. westbladi* ELamide peptides possess aromatic amino acids on position 3 and 5 from the C-terminus, which makes them somewhat similar to arthropod allatostatin A peptides (fig. 4*d*). The *N. westbladi* MCP complement differs from the other nemertodermatid species in having three LRIG paralogs (fig. 3*k*), two ELamide paralogs (fig. 3*l*), and a WDL peptide that ends in WDLGamide, instead of WDLamide (fig. 3*m*).

#### One Type of Novel MCP Is Plesiomorphic to *Xenoturbella*

The only potential MCP type that is specific to the two *Xenoturbella* species is the LRFDIamide (fig. 3*n*). The precursors of both species are very similar with four copies of the peptide in the same arrangement and an overall high conservation of the nonrepetitive peptides.

#### Full-Length MCPs without Orthologs Were Only Detected in Acoelomorph Species

We detected several putative neuropeptide precursors that were only identified in single acoelomorph species, without any orthologous sequences in other xenacoelomorph transcriptomes (fig. 4*a* and supplementary fig. 2, Supplementary Material online). A few partial sequences with a repetitive structure were found in *Xenoturbella* species, but all of them are missing the 5′-end; therefore, they could not be tested for the presence of a signal peptide. Such single repetitive sequences with a missing 5′-end were also found in acoel and nemertodermatid species. Full-length precursors with a signal peptide belonged exclusively to acoel and nemertodermatid species.

#### The Mass Spectrometric Analysis Confirms Several Types of MCPs in Acoel Species

Our mass spectrometric analysis of peptide extracts from three acoel species (*Convolutriloba macropyga*, *H. miamia*, and *I. pulchra*) was mainly used to confirm our bioinformatic predictions of the neuropeptide precursors. From the predicted peptide precursors, the presence of processed LWDY peptides, AWDF peptides, SSxxxF peptides, FxxxFamide peptides, and amidated as well as nonamidated MxGFG peptides was confirmed by LC-MS/MS in at least one species (see fig. 3*d*–*h*, supplementary precursor sequences and supporting mass spectrometric data files, Supplementary Material online). In addition, our LC-MS/MS analysis showed evidence for peptide candidates that either had no repetition of similar peptides or were lacking an N-terminal signal peptide; however, these peptides are not listed here (see additional preproneuropeptide candidates in the supplementary precursor sequences and the supporting mass spectrometric data, Supplementary Material online).

## Discussion

In our analyses, we found that a high number of peptidergic systems that are plesiomorphic to Nephrozoa are also present in Xenacoelomorpha, whereas most cnidarian neuropeptide GPCRs are not directly orthologous to these bilaterian GPCRs. In addition, we detected not only many MCPs that seem to be restricted to Xenacoelomorpha but also some MCPs that show a sequence similarity to neuropeptides that have so far only been reported in specific deuterostome or protostome clades. When we compare the peptidergic systems of *Xenoturbella*, Nemertodermatida, and Acoela, we observed differences that might reflect the differences in their nervous system anatomy.

The phylogenetic placement of the Xenacoelomorpha has been controversial in the past, and contradicting phylogenetic characteristics of different genes are, for example, also reflected by the varying affinities of the xenacoelomorph neuropeptide GPCRs to deuterostome and protostome sequences in our phylogenetic GPCR analysis (fig. 1*b*, supplementary fig. 3*a* and table 3, Supplementary Material online). Here, we discuss our results on the background of recent phylogenomic analyses which place Xenacoelomorpha as the sister group to Nephrozoa (Deuterostomia + Protostomia) (Srivastava et al. 2014; Cannon et al. 2016; Rouse et al. 2016). This position has previously been challenged by a phylogenomic study that instead suggests a sister-group relationship to Ambulacraria (Echinodermata + Hemichordata) (Philippe et al. 2011), albeit with low support (see also Giribet [2016] and Cannon et al. [2016] for a critical assessment and reanalysis). The alternative placements would have generally different implications on our understanding of bilaterian evolution, some of which are, for example, discussed in Lowe and Pani (2011) and Telford and Copley (2016). Also smaller analyses that only use up to 13 mitochondrial genes place xenacoelomorphs as either sister group to Chordata (Rouse et al. 2016) or sister group to all remaining deuterostomes (Philippe et al. 2011; Robertson et al. 2017). However, the general utility of such small mitochondrial data sets for resolving deep phylogenetic nodes has been questioned before (Bernt et al. 2013; Rouse et al. 2016). Even though we interpret our findings on the background of the position of Xenacoelomorpha as a sister group to all remaining Bilateria (Srivastava et al. 2014; Cannon et al. 2016; Rouse et al. 2016), we want to encourage the reader to also see the presented data from the view of an alternative placement of xenacoelomorphs as a deuterostome clade.

### The Early Expansion of the Bilaterian Peptidergic Systems Is Independent from the Origin of Complex Bilaterian Organ Systems

Our GPCR survey and phylogenetic analysis revealed a novel expansion into more than 20 different types of neuropeptide GPCRs in the bilaterian stem lineage. This is surprising, because it suggests that many of the extant nephrozoan peptidergic systems evolved and diverged before the split of Nephrozoa and Xenacoelomorpha. Because organ systems such as the circulatory system, excretory organs, or a through gut are absent from xenacoelomorphs (Hejnol and Martindale 2008; Haszprunar 2016), the major diversification of the bilaterian peptidergic system is not directly related to the origin of organ systems that characterize more complex animals. Neuropeptides in bilaterians are very divergent in their function and can trigger simple reactions to complex behaviors in animals. The early bilaterian neuropeptides thus likely were involved in different roles, as orthologous neuropeptides are not tightly associated with the same structures, functions, or behaviors in different clades. GnRH, for example, controls mammalian reproduction by stimulating the synthesis and release of follicle stimulating hormone and luteinizing hormone from the anterior pituitary (Okubo and Nagahama 2008), whereas its insect ortholog, the neuropeptide AKH, is involved in energy mobilization in different insects (Gade and Auerswald 2003). Ancestral peptides can also be related to clade- or species-specific behaviors, such as FMRFamide-related peptides to the startle behavior of the brachiopod *Terebratalia transversa* (Thiel et al. 2017), to chromatophore expansions in the cuttlefish *Sepia officinalis* (Loi and Tublitz 1997), or to the feeding behavior of the sea hare *Aplysia californica* (Vilim et al. 2010). Some studies that suggest a homology of deuterostomian and protostomian brains include overlapping expression of homologous neuropeptides in their line of evidence (Tessmar-Raible et al. 2007; Tosches and Arendt 2013; Arendt et al. 2016). An immunohistochemical study using antibodies against various neuropeptides in three dinophilid species (Annelida), however, shows that orthologous neuropeptides can be expressed in different brain regions, even in closely related species with morphologically similar brains (Kerbl et al. 2017). This indicates that a general long-term conservation of strictly defined local peptidergic expression is rather unlikely. Furthermore, nephrozoans that lack condensed brains, such as ambulacrarians, possess basically all bilaterian peptidergic systems (Jékely 2013; Mirabeau and Joly 2013; Semmens et al. 2016; Tian et al. 2016; Suwansa-Ard et al. 2018). The integration of existing neuropeptides into novel circuits and roles can, therefore, be rather plastic, and the ancestral peptidergic systems were later integrated into the complex circuits and behaviors that are known from deuterostomes and protostomes. Closer investigations of the function of neuropeptides in xenacoelomorphs and their implementation in neural circuits might, therefore, help to understand early roles of neuropeptides in Bilateria.

### Similarities of Xenacoelomorph MCPs with Clade-Specific Deuterostome and Protostome MCPs: Ancestral Peptides or Convergent Evolution?

In our analysis, we discovered MCPs that show similarities to MCPs that are only known from restricted deuterostome or protostome clades. The similarity to clade-specific neuropeptides might indicate that either these are homologous peptides with ancestral motifs or these motifs evolved convergently. Several acoel LxFamides that share the SxLHFamide motif and some of the SSxxxFamide peptides show sequence similarity to L-type SALMFamides from echinoderms (fig. 4*b*) (Elphick 2014; Elphick et al. 1991, 2015). It has also been reported that one of the antibodies that was raised against echinoderm SALMFamides shows immunoreactivity not only in different ambulacrarian species but also in *X. bocki* and three acoel species (Stach et al. 2005; Dittmann et al. 2018). These similarities would be in line with the previous notion of a phylogenetic affinity of Xenacoelomorpha to ambulacrarians (Philippe et al. 2011). Other xenacoelomorph MCPs, however, show similarities to neuropeptides that are only present in protostomes, such as the PxFVamide, which is similar to trochozoan PxFVamides (fig. 4*c*). Indeed, PxFVamides have been described in various mollusk species (Fujisawa et al. 1991; Veenstra 2010; Stewart et al. 2014; Adamson et al. 2015; Zatylny-Gaudin et al. 2016; Bose et al. 2017), and in the annelid *Capitella teleta* (Veenstra 2011), which altogether are referred to as “PxFVamide” (Veenstra 2010; Adamson et al. 2015; Bose et al. 2017) “FVamide” (Veenstra 2011) or “*Mytilus* inhibitory peptide” (Fujisawa et al. 1991; Stewart et al. 2014). Other peptides with similarity to protostome peptides are the ELamides; particularly two sequences from *N. westbladi* show similarity to arthropod allatostatin A peptides (fig. 4*d*). Allatostatin A’s are MCPs that are especially known from arthropods and share the C-terminal ending [Y/F]xFGLamide (Woodhead et al. 1989, 1994; Pratt et al. 1991; Wegener and Gorbashov 2008; Hauser et al. 2010; Nassel and Winther 2010; Jékely 2013) with somewhat similar neuropeptides in trochozoans, such as buccalins (Veenstra 2010; Conzelmann et al. 2013; Bose et al. 2017). Orthologs of the allatostatin A receptors are present in Xenacoelomorpha, including *N. westbladi*. The receptors of the echinoderm SALMFamides and the trochozoan PxFVamides are to this date unknown, which makes it so far impossible to test the potential binding affinities. The general variability of the SALMFamides in echinoderms and the PxFVamides in trochozoans, however, shows that these peptides have a high evolutionary rate within those animal groups. Due to the short length and the high diversity of closely related orthologs of MCPs, the possibility of a convergent evolution of similar motifs in distantly related taxa is likely. RFamide-type neuropeptides, for example, share a similar C-terminal motif, but not all of them are homologous (Espinoza et al. 2000; Walker et al. 2009; Jékely 2013; Mirabeau and Joly 2013; Elphick and Mirabeau 2014; Peymen et al. 2014).

### Changes in the Neuropeptide Complement Correlate with the Evolution of Nervous System Architectures in Xenacoelomorphs

**Fig. 5.**
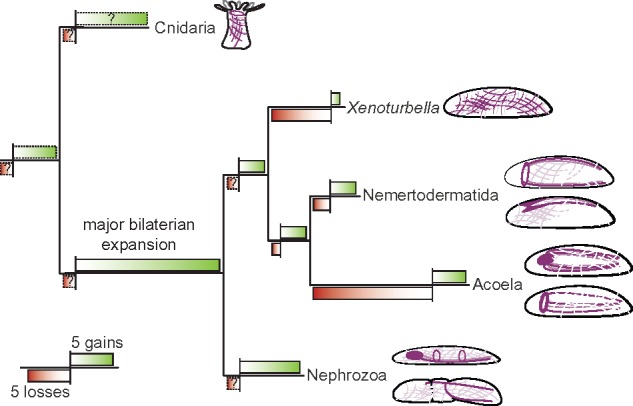
Changes in the complement of peptidergic systems during bilaterian evolution. Tree shows major expansion of peptidergic systems in the ancestral bilaterian lineage followed by clade-specific changes in sublineages. Bars show number of changes as detected losses (red bars underneath the branches) and detected gains (green bars above the branches) of peptidergic systems in the corresponding lineage. Length of bars are estimated based on the results of this study, with 4–5 peptidergic systems in the cnidarian–bilaterian ancestor that gave rise to the main diversification in the bilaterian lineage (excluding relaxin GPCRs and GPH GPCRs). The two bars on the lower left side indicate the bar-length for five gains or five losses. Cartoons depict simplified examples of nervous system anatomies (purple) from representative taxa (top to bottom: anthozoan, *Xenoturbella bocki*, *Nemertoderma westbladi*, *Meara stichopi*, *Isodiametra pulchra*, *Dipisthoporus longitubus*, annelid, and hemichordate).

The common notion is that the ancestral xenacoelomorph possessed a basiepidermal nerve net (Hejnol 2015a; Hejnol and Rentzsch 2015; Gavilan et al. 2016; Haszprunar 2016; Raikova et al. 2016; Martin-Duran et al. 2018). Because many of the ancestral bilaterian peptidergic systems are present in *Xenoturbella*, which only possess a basiepidermal nerve net (Raikova et al. 2000a; Stach 2015), it suggests that the presence of the bilaterian neuropeptide systems seems not to be correlated to the presence of a condensed CNS with brain-like structures. Brains and nerve cords are only present in acoels and nemertodermatids, and these structures seem to have evolved independently from the CNS of other bilaterians (Gavilan et al. 2016; Haszprunar 2016; Martin-Duran et al. 2018). While the nemertodermatid nerve condensations are usually situated in a basiepidermal position, the acoel nervous system components are generally internalized, which is considered to be a derived state in the Xenacoelomorpha (fig. 5) (Raikova et al. 2000b, 2004, 2016; Achatz and Martinez 2012; Hejnol 2015a; Gavilan et al. 2016; Haszprunar 2016; Hejnol and Pang 2016; Dittmann et al. 2018). Interestingly, our results show that acoels have a great reduction of the conserved bilaterian peptidergic systems, which are still present in nemertodermatids. This observation is in line with a previous genomic survey that describes the presence of only a subset of conserved bilaterian GPCRs in the acoel *Symsagittifera roscoffensis* (Perea-Atienza et al. 2015). On the other hand, we also observe a gain of novel neuropeptides in the acoel lineages, similar to the nemertodermatid lineages. The expansion of MCPs in both acoelomorph lineages correlates with the formation of nervous system condensations in nemertodermatids and acoels, which could indicate a connection between a gain of morphological nervous system complexity and an expansion of the peptidergic complement. While it is difficult to correlate the origin of the bilaterian peptidergic system with the formation of a CNS, our study suggests that morphological novelties of the nervous system can be accompanied by a change in the peptidergic complement that comprises differential gains and losses. To further investigate this correlation and how it relates to the nervous system function (e.g., which circuits have been lost, changed or gained), more detailed characterizations of neuropeptides in xenacoelomorphs including their localization and function are necessary. By doing so, we would provide a better understanding of the origins of bilaterian nervous system evolution.

## Conclusion

This study shows that the major diversification of the peptidergic signaling that is plesiomorphic to deuterostomes and protostomes occurred after the cnidarian–bilaterian evolutionary split, along the stem leading to Nephrozoa and Xenacoelomorpha (fig. 5). This result, therefore, contradicts previous hypotheses that proposed the correlation of the evolution of the sophisticated bilaterian peptidergic systems with the origin of complex organ systems. The differential gains and losses of neuropeptide systems within xenacoelomorphs, however, do correlate with morphological novelties of their nervous systems, which mirrors the correlation of neuropeptide complement changes during the evolution of complex bilaterian brains (fig. 5). Therefore, our in silico analysis provides the basis for future research on neuropeptides within the nervous systems of xenacoelomorphs. This way, one might be able to further test a connection between the ancestral changes in peptidergic signaling and an increase in nervous system complexity.

## Materials and Methods

### Sequence Data

We investigated assembled transcriptomes from 13 Xenacoelomorpha species deposited into public databases. These species include: *Childia submaculatum* (Acoela), *C. macropyga* (Acoela), *D. gymnopharyngeus* (Acoela), *D. longitubus* (Acoela), *Eumecynostomum macrobursalium* (Acoela), *H. miamia* (Acoela), *I. pulchra* (Acoela), *Ascoparia* sp. (Nemertodermatida), *M. stichopi* (Nemertodermatida), *N. westbladi* (Nemertodermatida), *Sterreria* sp. (Nemertodermatida), *X. bocki* (*Xenoturbella*), *X. profunda* (*Xenoturbella*). Most of the transcriptomes were generated from several whole adults, except for *X. profunda*, and should thus include neuronal expressions (see supplementary table 4, Supplementary Material online, for more details). We collected neuropeptide precursor and neuropeptide receptor sequences from previous publications (Hauser et al. 2010; Veenstra 2011; Conzelmann et al. 2013; Jékely 2013; Mirabeau and Joly 2013; Adamson et al. 2015; Bauknecht and Jékely 2015; Semmens et al. 2016; Suwansa-Ard et al. 2018) and public databases (i.e., NCBI and UniProt) for further analyses. We aimed for a broad sampling across Bilateria and covered different clades of chordates (i.e., Craniata, Cephalochordata, and Urochordata), ambulacrarians (i.e, Echinodermata and Hemichordata), ecdysozoans (i.e., Nematoda and Arthropoda), and spiralians (i.e., Molluska and Annelida). To increase the amount of ambulacrarian sequences in our phylogenetic analyses, we included additional echinoderm and hemichordate transcriptomes and genome-derived proteomes. The echinoderm and hemichordate species included *Astrotoma agssizii*, *Labidiaster annulatus*, *Leptosynapta clarki*, *Saccoglossus mereschkowskii*, *Ptychodera flava*, and *Acanthaster planci* (see supplementary table 5, Supplementary Material online, for more details).

To compare the bilaterian neuropeptide GPCRs with the cnidarian neuropeptide GPCRs, we used a diverse set of receptors that were predicted by the automated eukaryotic NCBI annotation pipeline from the genomes of the anthozoans *Exaiptasia pallida*, *Acropora digitifera* and *Orbicella faveolata*, as well as receptor sequences of *Nema. vectensis* that were already predicted and published (Anctil 2009).

### Identification and Analysis of Neuropeptide GPCRs

GPCR sequences were clustered using CLANS2 (Frickey and Lupas 2004), and a subset of diverse sequences from each group was used as query sequences in TBlastN searching with an *e*-value cutoff of 1e-30. All resulting sequences from xenacoelomorphs and ambulacrarians were used as new query sequences for an additional search to find potential hidden orthologs (Martin-Duran et al. 2017). We analyzed all retrieved sequences in a cluster analysis using CLANS2 (Frickey and Lupas 2004) with the standard BlastP BLOSUM 62 scoring matrix. Sequences for phylogenetic trees were aligned with ClustalX v2.1 (Larkin et al. 2007), nonconserved regions were automatically removed with TrimAl (Capella-Gutierrez et al. 2009), and phylogenetic trees were generated with RAxML v8.2.9 (Stamatakis 2014) and FastTree v2.1 (Price et al. 2010) using the LG amino acid substitution model. Phylogenetic trees were visualized with FigTree v1.4.3 (http://tree.bio.ed.ac.uk/software/figtree; last accessed August 23, 2018).

### Identification of Neuropeptide Precursors

Preproneuropeptide sequences of related neuropeptides were compared using CLANS2, and a set of diverse sequences was used as query sequences. The reference sequences were used in TBlastN searching using BLOSUM62 and BLOSUM45 substitution matrices with an *e*-value cutoff of 1. In addition to sequence similarity search, we employed a Perl script that uses PROSITE pattern syntax and flat files to detect specific protein sequence motifs. We screened translated transcriptomes for the presence of recurrent cleavage and amidation sites that are commonly found in multicopy neuropeptide precursor sequences, including x(5, 200)-G-[KR]-[KR]-x(2, 35)-G-[KR]-[KR]-x(2, 35)-G-[KR](0, 1) and x(5, 200)-[KR]-[KR]-x(2, 35)-[KR]-[KR]-x(2, 35)-[KR]-[KR]-x(2, 35)-[KR]-[KR]. For possible RFamides with alternative monobasic cleavage sites, we searched for the following protein sequence motif: x(5, 200)-R-F-G-[KR]-x(2, 35)-R-F-G-[KR]-x(2, 35)-R-F-G-[KR](0, 1). This approach gave us candidate neuropeptides that were further analyzed manually for recurrent peptide sequences between the cleavage sites. All sequences from the motif and similarity search with complete 5′-ends were tested for the presence of a signal peptide with Signal-3L 2.0 (Zhang and Shen 2017) as well as with SignalP 4.1 (Petersen et al. 2011) using a *D*-cutoff value of 0.34. Cleavage sites were predicted at dibasic sites [R/K]-[R/K], and alternative monobasic cleavage sites were predicted with the online application of NeuroPred (http://stagbeetle.animal.uiuc.edu; last accessed August 23, 2018) (Southey et al. 2006). A C-terminal glycine residue in the predicted peptides was used as a signal for C-terminal amidation of the prior amino acid residue. Results were manually examined, and the positive hits were used for a reciprocal search in all transcriptomes to identify hidden orthologs, as described by Martin-Duran et al. (2017). Peptide sequence logo representations and precursor diagrams were created with CLC Main Workbench (Qiagen Bioinformatics). All figures were created using Adobe Illustrator CS6 with the phylogenetic relationships among Xenacoelomorpha based on Cannon et al. (2016) and Jondelius et al. (2011).

### Peptide Extraction and Mass Spectrometry (LC-MS/MS)

We extracted peptides from 5 to 10 specimens of whole animals of the acoels *C. macropyga*, *I. pulchra*, and *H. miamia* following the protocol described by Conzelmann et al. (2013). Collected specimens were rinsed with distilled water, transferred into the extraction buffer (90% methanol, 9% acetic acid, and 1% distilled water), grinded with a pestle, and vortexed vigorously. The suspension was centrifuged at maximum speed for 20 min at 4 °C. The supernatant was collected, completely evaporated in a vacuum concentrator, and dissolved in 200 µl of ultrapure water. Neuropeptide mixtures were reduced and alkylated as described by Borchert et al. (2010) and desalted with C_18_ StageTips (Rappsilber et al. 2007).

LC-MS analysis was performed on an EasyLC nano-UHPLC (Thermo Scientific) coupled with a Q Exactive HF mass spectrometer (Thermo Scientific). Separations of the peptide mixtures were performed as previously described (Conzelmann et al. 2013) with slight modifications. Peptides were eluted with a 57-min segmented gradient of 10–20–50–90% HPLC solvent B (80% acetonitrile in 0.1% formic acid). The mass spectrometer was operated in the positive ion mode. Full scan was acquired in the mass range from *m*/*z* 300 to 1650 at a resolution of 120,000 followed by Higher energy collisional dissociation (HCD) fragmentation of the seven most intense precursor ions. High-resolution HCD MS/MS spectra were acquired with a resolution of 60,000. The target values for the MS scan and MS/MS fragmentation were 3 × 10^6^ and 10^5^ charges with a maximum fill time of 25 and 110 ms, respectively. Precursor ions were excluded from sequencing for 30 s after MS/MS. MS/MS on singly charged precursor ions was enabled. The acquired MS raw files were processed using the MaxQuant software suite v.1.5.2.8 (Cox and Mann 2008). Extracted peak lists were submitted to database search using the Andromeda search engine (Cox et al. 2011) to query target-decoy databases consisting of the predicted propeptides and the predicted active neuropeptides, commonly observed contaminants (285 entries), and the reversed complements of those sequences. Cleavage specificity for N- and C-terminal of arginine and lysine and no enzyme definition were defined. The minimal peptide length was set to four amino acids. The initial precursor mass tolerance was set to 4.5 ppm; for fragment ions, a mass tolerance of 20 ppm was used. Carbamidomethylation of cysteines was defined as fixed modification in the database search. A set of expected variable modifications was defined in the database search: Oxidation of methionine, acetylation of the peptide N-terminus, amidation of the peptide C-terminus, and sulfation of tyrosine. False discovery rates were set to 1% at peptide, modification site, and protein group level, estimated by the target/decoy approach (Elias and Gygi 2007). Spectra of peptides having scores below 100 were validated manually.

## Data Accession

Amino acid sequences of xenacoelomorph neuropeptide precursors and neuropeptide GPCRs are available in the supplementary material, Supplementary Material online. Supporting files that include original alignments, RAxML and FastTree output files, CLANS files, amino acid sequences of GPCRs and precursors, and mass spectrometric data are available on figshare https://doi.org/10.6084/m9.figshare.c.4064639.v1; last accessed August 23, 2018 and https://doi.org/10.6084/m9.figshare.6287759; last accessed August 23, 2018. The script for the pattern searches is available on https://github.com/faguil/protein-motif-searching_neuropeptides; last accessed August 23, 2018.

## Supplementary Material

Supplementary Data

## References

[msy160-B1] Achatz JG, Martinez P. 2012 The nervous system of *Isodiametra pulchra* (Acoela) with a discussion on the neuroanatomy of the Xenacoelomorpha and its evolutionary implications. Front Zool. 9 1: 27. 23072457 10.1186/1742-9994-9-27 PMC3488495

[msy160-B2] Adamson KJ, Wang T, Zhao M, Bell F, Kuballa AV, Storey KB, Cummins SF. 2015 Molecular insights into land snail neuropeptides through transcriptome and comparative gene analysis. BMC Genomics 16:308. 25884396 10.1186/s12864-015-1510-8 PMC4408573

[msy160-B3] Alzugaray ME, Adami ML, Diambra LA, Hernandez-Martinez S, Damborenea C, Noriega FG, Ronderos JR. 2013 Allatotropin: an ancestral myotropic neuropeptide involved in feeding. PLoS One 8 10: e77520. 24143240 10.1371/journal.pone.0077520 PMC3797082

[msy160-B4] Alzugaray ME, Hernandez-Martinez S, Ronderos JR. 2016 Somatostatin signaling system as an ancestral mechanism: myoregulatory activity of an Allatostatin-C peptide in *Hydra*. Peptides 82:67–75. 27288244 10.1016/j.peptides.2016.05.011

[msy160-B5] Anctil M. 2009 Chemical transmission in the sea anemone *Nematostella vectensis*: a genomic perspective. Comp Biochem Physiol Part D Genomics Proteomics. 4 4: 268–289. 20403752 10.1016/j.cbd.2009.07.001

[msy160-B6] Arendt D, Tosches MA, Marlow H. 2016 From nerve net to nerve ring, nerve cord and brain–evolution of the nervous system. Nat Rev Neurosci. 17 1: 61–72. 26675821 10.1038/nrn.2015.15

[msy160-B7] Bauknecht P, Jékely G. 2015 Large-scale combinatorial deorphanization of *Platynereis* neuropeptide GPCRs. Cell Rep. 12 4: 684–693. 26190115 10.1016/j.celrep.2015.06.052

[msy160-B8] Baumgarten S, Simakov O, Esherick LY, Liew YJ, Lehnert EM, Michell CT, Li Y, Hambleton EA, Guse A, Oates ME. 2015 The genome of *Aiptasia*, a sea anemone model for coral symbiosis. Proc Natl Acad Sci U S A. 112 38: 11893–11898. 26324906 10.1073/pnas.1513318112 PMC4586855

[msy160-B9] Beets I, Temmerman L, Janssen T, Schoofs L. 2013 Ancient neuromodulation by vasopressin/oxytocin-related peptides. Worm 2 2: e24246. 24058873 10.4161/worm.24246 PMC3704447

[msy160-B10] Bernt M, Bleidorn C, Braband A, Dambach J, Donath A, Fritzsch G, Golombek A, Hadrys H, Juhling F, Meusemann K, et al 2013 A comprehensive analysis of bilaterian mitochondrial genomes and phylogeny. Mol Phylogenet Evol. 69 2: 352–364. 23684911 10.1016/j.ympev.2013.05.002

[msy160-B11] Borchert N, Dieterich C, Krug K, Schutz W, Jung S, Nordheim A, Sommer RJ, Macek B. 2010 Proteogenomics of *Pristionchus pacificus* reveals distinct proteome structure of nematode models. Genome Res. 20 6: 837–846. 20237107 10.1101/gr.103119.109 PMC2877580

[msy160-B12] Børve A, Hejnol A. 2014 Development and juvenile anatomy of the nemertodermatid *Meara stichopi* (Bock) Westblad 1949 (Acoelomorpha). Front Zool. 11:50. 25024737 10.1186/1742-9994-11-50 PMC4094782

[msy160-B13] Bose U, Suwansa-Ard S, Maikaeo L, Motti CA, Hall MR, Cummins SF. 2017 Neuropeptides encoded within a neural transcriptome of the giant triton snail *Charonia tritonis*, a Crown-of-Thorns starfish predator. Peptides 98:3–14. 28082215 10.1016/j.peptides.2017.01.004

[msy160-B14] Cannell E, Dornan AJ, Halberg KA, Terhzaz S, Dow JAT, Davies SA. 2016 The corticotropin-releasing factor-like diuretic hormone 44 (DH44) and kinin neuropeptides modulate desiccation and starvation tolerance in *Drosophila melanogaster*. Peptides 80:96–107. 26896569 10.1016/j.peptides.2016.02.004 PMC4889782

[msy160-B15] Cannon JT, Vellutini BC, Smith J 3rd, Ronquist F, Jondelius U, Hejnol A. 2016 Xenacoelomorpha is the sister group to Nephrozoa. Nature 530 7588: 89–93. 26842059 10.1038/nature16520

[msy160-B16] Capella-Gutierrez S, Silla-Martinez JM, Gabaldon T. 2009 trimAl: a tool for automated alignment trimming in large-scale phylogenetic analyses. Bioinformatics 25 15: 1972–1973. 19505945 10.1093/bioinformatics/btp348 PMC2712344

[msy160-B17] Catak Z, Aydin S, Sahin I, Kuloglu T, Aksoy A, Dagli AF. 2014 Regulatory neuropeptides (ghrelin, obestatin and nesfatin-1) levels in serum and reproductive tissues of female and male rats with fructose-induced metabolic syndrome. Neuropeptides 48 3: 167–177. 24786976 10.1016/j.npep.2014.04.002

[msy160-B18] Christie AE, Skiebe P, Marder E. 1995 Matrix of neuromodulators in neurosecretory structures of the crab *Cancer borealis*. J Exp Biol. 198(Pt 12): 2431–2439. 8576680 10.1242/jeb.198.12.2431

[msy160-B19] Chung BY, Ro J, Hutter SA, Miller KM, Guduguntla LS, Kondo S, Pletcher SD. 2017 *Drosophila* neuropeptide F signaling independently regulates feeding and sleep-wake behavior. Cell Rep. 19 12: 2441–2450. 28636933 10.1016/j.celrep.2017.05.085 PMC5536846

[msy160-B20] Conzelmann M, Jékely G. 2012 Antibodies against conserved amidated neuropeptide epitopes enrich the comparative neurobiology toolbox. Evodevo 3 1: 23. 23020891 10.1186/2041-9139-3-23 PMC3503879

[msy160-B21] Conzelmann M, Williams EA, Krug K, Franz-Wachtel M, Macek B, Jékely G. 2013 The neuropeptide complement of the marine annelid *Platynereis dumerilii*. BMC Genomics 14:906. 24359412 10.1186/1471-2164-14-906 PMC3890597

[msy160-B22] Cox J, Mann M. 2008 MaxQuant enables high peptide identification rates, individualized p.p.b.-range mass accuracies and proteome-wide protein quantification. Nat Biotechnol. 26 12: 1367–1372. 19029910 10.1038/nbt.1511

[msy160-B23] Cox J, Neuhauser N, Michalski A, Scheltema RA, Olsen JV, Mann M. 2011 Andromeda: a peptide search engine integrated into the MaxQuant environment. J Prot Res. 10 4: 1794–1805. 10.1021/pr101065j 21254760

[msy160-B24] Cropper EC, Jing J, Vilim FS, Barry MA, Weiss KR. 2018 Multifaceted expression of peptidergic modulation in the feeding system of *Aplysia*. ACS Chem Neurosci. 9 8: 1917–1927. 29309115 10.1021/acschemneuro.7b00447 PMC9719044

[msy160-B25] De Mota N, Goazigo ARL, El Messari S, Chartrel N, Roesch D, Dujardin C, Kordon C, Vaudry H, Moos FO, Llorens-Cortes C. 2004 Apelin, a potent diuretic neuropeptide counteracting vasopressin actions through inhibition of vasopressin neuron activity and vasopressin release. Proc Natl Acad Sci U S A. 101 28: 10464–10469. 15231996 10.1073/pnas.0403518101 PMC478592

[msy160-B26] Diao F, Elliott AD, Diao F, Shah S, White BH. 2017 Neuromodulatory connectivity defines the structure of a behavioral neural network. Elife 6:e29797. 29165248 10.7554/eLife.29797 PMC5720592

[msy160-B27] Dickinson PS, Calkins A, Stevens JS. 2015 Related neuropeptides use different balances of unitary mechanisms to modulate the cardiac neuromuscular system in the American lobster, *Homarus americanus*. J Neurophysiol. 113 3: 856–870. 25392168 10.1152/jn.00585.2014 PMC4312872

[msy160-B28] Dittmann IL, Zauchner T, Nevard LM, Telford MJ, Egger B. 2018 SALMFamide2 and serotonin immunoreactivity in the nervous system of some acoels (Xenacoelomorpha). J Morphol. 279 5: 589–597. 29388261 10.1002/jmor.20794 PMC5947262

[msy160-B29] Elias JE, Gygi SP. 2007 Target-decoy search strategy for increased confidence in large-scale protein identifications by mass spectrometry. Nat Methods. 4 3: 207–214. 17327847 10.1038/nmeth1019

[msy160-B30] Elphick MR. 2014 SALMFamide salmagundi: the biology of a neuropeptide family in echinoderms. Gen Comp Endocrinol. 205:23–35. 24583124 10.1016/j.ygcen.2014.02.012

[msy160-B31] Elphick MR, Mirabeau O. 2014 The evolution and variety of RFamide-type neuropeptides: insights from deuterostomian invertebrates. Front Endocrinol. 5:93. 10.3389/fendo.2014.00093 PMC4062910 24994999

[msy160-B32] Elphick MR, Mirabeau O, Larhammar D. 2018 Evolution of neuropeptide signalling systems. J Exp Biol. 221 3: jeb151092. 29440283 10.1242/jeb.151092 PMC5818035

[msy160-B33] Elphick MR, Price DA, Lee TD, Thorndyke MC. 1991 The SALMFamides: a new family of neuropeptides isolated from an echinoderm. Proc Biol Sci. 243 1307: 121–127. 1676515 10.1098/rspb.1991.0020

[msy160-B34] Elphick MR, Semmens DC, Blowes LM, Levine J, Lowe CJ, Arnone MI, Clark MS. 2015 Reconstructing SALMFamide neuropeptide precursor evolution in the phylum Echinodermata: ophiuroid and crinoid sequence data provide new insights. Front Endocrinol. 6:2. 10.3389/fendo.2015.00002 PMC4313774 25699014

[msy160-B35] Espinoza E, Carrigan M, Thomas SG, Shaw G, Edison AS. 2000 A statistical view of FMRFamide neuropeptide diversity. Mol Neurobiol. 21(1–2): 35–56. 11327149 10.1385/MN:21:1-2:035

[msy160-B36] Frickey T, Lupas A. 2004 CLANS: a Java application for visualizing protein families based on pairwise similarity. Bioinformatics 20 18: 3702–3704. 15284097 10.1093/bioinformatics/bth444

[msy160-B37] Frooninckx L, Van Rompay L, Temmerman L, Van Sinay E, Beets I, Janssen T, Husson SJ, Schoofs L. 2012 Neuropeptide GPCRs in *C. elegans*. Front Endocrinol. 3:167. 10.3389/fendo.2012.00167 PMC3527849 23267347

[msy160-B38] Fujino Y, Nagahama T, Oumi T, Ukena K, Morishita F, Furukawa Y, Matsushima O, Ando M, Takahama H, Satake H, et al 1999 Possible functions of oxytocin/vasopressin-superfamily peptides in annelids with special reference to reproduction and osmoregulation. J Exp Zool. 284 4: 401–406. 10451417 10.1002/(sici)1097-010x(19990901)284:4<401::aid-jez6>3.3.co;2-l

[msy160-B39] Fujisawa Y, Kubota I, Ikeda T, Minakata H, Muneoka Y. 1991 A variety of *Mytilus* inhibitory peptides in the ABRM of *Mytilus edulis*: isolation and characterization. Comp Biochem Physiol C. 100 3: 525–531. 1687551

[msy160-B40] Gade G, Auerswald L. 2003 Mode of action of neuropeptides from the adipokinetic hormone family. Gen Comp Endocrinol. 132 1: 10–20. 12765639 10.1016/s0016-6480(03)00159-x

[msy160-B41] Gavilan B, Perea-Atienza E, Martinez P. 2016 Xenacoelomorpha: a case of independent nervous system centralization? Philos Trans R Soc Lond B Biol Sci. 371 1685: 20150039. 26598722 10.1098/rstb.2015.0039 PMC4685578

[msy160-B42] Giribet G. 2016 Genomics and the animal tree of life: conflicts and future prospects. Zool Scr. 45:14–21.

[msy160-B43] Grimmelikhuijzen CJ, Hauser F. 2012 Mini-review: the evolution of neuropeptide signaling. Regul Pept. 177(Suppl):S6–S9. 22726357 10.1016/j.regpep.2012.05.001

[msy160-B44] Grimmelikhuijzen CJP, Williamson M, Hansen GN. 2002 Neuropeptides in cnidarians. Can J Zool. 80 10: 1690–1702.

[msy160-B45] Halberg KA, Terhzaz S, Cabrero P, Davies SA, Dow JA. 2015 Tracing the evolutionary origins of insect renal function. Nat Commun. 6:6800. 25896425 10.1038/ncomms7800 PMC4410669

[msy160-B46] Hansen KK, Stafflinger E, Schneider M, Hauser F, Cazzamali G, Williamson M, Kollmann M, Schachtner J, Grimmelikhuijzen CJ. 2010 Discovery of a novel insect neuropeptide signaling system closely related to the insect adipokinetic hormone and corazonin hormonal systems. J Biol Chem. 285 14: 10736–10747. 20068045 10.1074/jbc.M109.045369 PMC2856281

[msy160-B47] Haszprunar G. 2016 Review of data for a morphological look on Xenacoelomorpha (*Bilateria incertae sedis*). Org Divers Evol. 16 2: 363–389.

[msy160-B48] Hauser F, Grimmelikhuijzen CJP. 2014 Evolution of the AKH/corazonin/ACP/GnRH receptor superfamily and their ligands in the Protostomia. Gen Comp Endocrinol. 209:35–49. 25058364 10.1016/j.ygcen.2014.07.009

[msy160-B49] Hauser F, Neupert S, Williamson M, Predel R, Tanaka Y, Grimmelikhuijzen CJ. 2010 Genomics and peptidomics of neuropeptides and protein hormones present in the parasitic wasp *Nasonia vitripennis*. J Proteome Res. 9 10: 5296–5310. 20695486 10.1021/pr100570j

[msy160-B50] Hejnol A. 2015a Acoelomorpha In: Schmidt-Rhaesa A, Harzsch S, Purschke G, editors. Structure and evolution of invertebrate nervous systems. Oxford (UK): Oxford University Press p. 56–61.

[msy160-B51] Hejnol A. 2015b Acoelomorpha and Xenoturbellida In: Wanninger A, editor. Evolutionary developmental biology of invertebrates 1. Vienna (Austria): Springer p. 203–214.

[msy160-B52] Hejnol A, Lowe CJ. 2015 Embracing the comparative approach: how robust phylogenies and broader developmental sampling impacts the understanding of nervous system evolution. Philos Trans R Soc Lond B Biol Sci. 370 1684: 20150045. 26554039 10.1098/rstb.2015.0045 PMC4650123

[msy160-B53] Hejnol A, Martindale MQ. 2008 Acoel development supports a simple planula-like urbilaterian. Philos Trans R Soc Lond B Biol Sci. 363 1496: 1493–1501. 18192185 10.1098/rstb.2007.2239 PMC2614228

[msy160-B54] Hejnol A, Obst M, Stamatakis A, Ott M, Rouse GW, Edgecombe GD, Martinez P, Baguna J, Bailly X, Jondelius U, et al 2009 Assessing the root of bilaterian animals with scalable phylogenomic methods. Proc Biol Sci. 276 1677: 4261–4270. 19759036 10.1098/rspb.2009.0896 PMC2817096

[msy160-B55] Hejnol A, Pang K. 2016 Xenacoelomorpha’s significance for understanding bilaterian evolution. Curr Opin Genet Dev. 39:48–54. 27322587 10.1016/j.gde.2016.05.019

[msy160-B56] Hejnol A, Rentzsch F. 2015 Neural nets. Curr Biol. 25 18: R782–R786. 26394095 10.1016/j.cub.2015.08.001

[msy160-B57] Hökfelt T, Broberger C, Xu ZQ, Sergeyev V, Ubink R, Diez M. 2000 Neuropeptides—an overview. Neuropharmacology 39 8: 1337–1356. 10818251 10.1016/s0028-3908(00)00010-1

[msy160-B58] Holzer P, Guth PH. 1991 Neuropeptide control of rat gastric mucosal blood flow. Increase by calcitonin gene-related peptide and vasoactive intestinal polypeptide, but not substance P and neurokinin A. Circ Res. 68 1: 100–105. 1702035 10.1161/01.res.68.1.100

[msy160-B59] Janssen T, Meelkop E, Lindemans M, Verstraelen K, Husson SJ, Temmerman L, Nachman RJ, Schoofs L. 2008 Discovery of a cholecystokinin-gastrin-like signaling system in nematodes. Endocrinology 149 6: 2826–2839. 18339709 10.1210/en.2007-1772

[msy160-B60] Jékely G. 2013 Global view of the evolution and diversity of metazoan neuropeptide signaling. Proc Natl Acad Sci U S A. 110 21: 8702–8707. 23637342 10.1073/pnas.1221833110 PMC3666674

[msy160-B61] Jékely G, Melzer S, Beets I, Kadow ICG, Koene J, Haddad S, Holden-Dye L. 2018 The long and the short of it—a perspective on peptidergic regulation of circuits and behaviour. J Exp Biol. 221 3: jeb166710. 29439060 10.1242/jeb.166710

[msy160-B62] Jondelius U, Wallberg A, Hooge M, Raikova OI. 2011 How the worm got its pharynx: phylogeny, classification and bayesian assessment of character evolution in Acoela. Syst Biol. 60 6: 845–871. 21828080 10.1093/sysbio/syr073

[msy160-B63] Kerbl A, Conzelmann M, Jékely G, Worsaae K. 2017 High diversity in neuropeptide immunoreactivity patterns among three closely related species of Dinophilidae (Annelida). J Comp Neurol. 525 17: 3596–3635. 28744909 10.1002/cne.24289

[msy160-B64] Kotikova EA, Raiikova OI. 2008 Architectonics of the central nervous system in Acoela, Plathelminthes, and Rotifera. Zh Evol Biokhim Fiziol. 44:83–93. 18411518

[msy160-B65] Krishnan A, Schioth HB. 2015 The role of G protein-coupled receptors in the early evolution of neurotransmission and the nervous system. J Exp Biol. 218(Pt 4): 562–571. 25696819 10.1242/jeb.110312

[msy160-B66] Larkin MA, Blackshields G, Brown NP, Chenna R, McGettigan PA, McWilliam H, Valentin F, Wallace IM, Wilm A, Lopez R, et al 2007 Clustal W and Clustal X version 2.0. Bioinformatics 23 21: 2947–2948. 17846036 10.1093/bioinformatics/btm404

[msy160-B67] Li S, Hauser F, Skadborg SK, Nielsen SV, Kirketerp-Moller N, Grimmelikhuijzen CJ. 2016 Adipokinetic hormones and their G protein-coupled receptors emerged in Lophotrochozoa. Sci Rep. 6:32789. 27628442 10.1038/srep32789 PMC5024129

[msy160-B68] Lindemans M, Janssen T, Beets I, Temmerman L, Meelkop E, Schoofs L. 2011 Gonadotropin-releasing hormone and adipokinetic hormone signaling systems share a common evolutionary origin. Front Endocrinol. 2:16. 10.3389/fendo.2011.00016 PMC3356000 22649364

[msy160-B69] Lindemans M, Liu F, Janssen T, Husson SJ, Mertens I, Gade G, Schoofs L. 2009 Adipokinetic hormone signaling through the gonadotropin-releasing hormone receptor modulates egg-laying in *Caenorhabditis elegans*. Proc Natl Acad Sci U S A. 106 5: 1642–1647. 19164555 10.1073/pnas.0809881106 PMC2629444

[msy160-B70] Loi PK, Tublitz N. 1997 Molecular analysis of FMRFamide- and FMRFamide-related peptides (FaRPS) in the cuttlefish *Sepia officinalis*. J Exp Biol. 200(Pt 10): 1483–1489. 9192498 10.1242/jeb.200.10.1483

[msy160-B71] Lowe CJ, Pani AM. 2011 Animal evolution: a soap opera of unremarkable worms. Curr Biol. 21 4: R151–R153. 21334293 10.1016/j.cub.2010.12.017

[msy160-B72] Martin-Duran JM, Pang K, Børve A, Le HS, Furu A, Cannon JT, Jondelius U, Hejnol A. 2018 Convergent evolution of bilaterian nerve cords. Nature 553 7686: 45–50. 29236686 10.1038/nature25030 PMC5756474

[msy160-B73] Martin-Duran JM, Ryan JF, Vellutini BC, Pang K, Hejnol A. 2017 Increased taxon sampling reveals thousands of hidden orthologs in flatworms. Genome Res. 27 7: 1263–1272. 28400424 10.1101/gr.216226.116 PMC5495077

[msy160-B74] Martínez P, Hartenstein V, Sprecher SG. 2017 Xenacoelomorpha nervous systems. Oxford Research Encyclopedia of Neuroscience, Oxford, UK: Oxford University Press.

[msy160-B75] McVeigh P, Mair GR, Atkinson L, Ladurner P, Zamanian M, Novozhilova E, Marks NJ, Day TA, Maule AG. 2009 Discovery of multiple neuropeptide families in the phylum Platyhelminthes. Int J Parasitol. 39 11: 1243–1252. 19361512 10.1016/j.ijpara.2009.03.005 PMC2749192

[msy160-B76] Mirabeau O, Joly JS. 2013 Molecular evolution of peptidergic signaling systems in bilaterians. Proc Natl Acad Sci U S A. 110 22: E2028–E2037. 23671109 10.1073/pnas.1219956110 PMC3670399

[msy160-B77] Mizoguchi A, Okamoto N. 2013 Insulin-like and IGF-like peptides in the silkmoth *Bombyx mori:* discovery, structure, secretion, and function. Front Physiol. 4:217. 23966952 10.3389/fphys.2013.00217 PMC3745042

[msy160-B78] Nassel DR, Wegener C. 2011 A comparative review of short and long neuropeptide F signaling in invertebrates: any similarities to vertebrate neuropeptide Y signaling? Peptides 32 6: 1335–1355. 21440021 10.1016/j.peptides.2011.03.013

[msy160-B79] Nassel DR, Winther AM. 2010 Drosophila neuropeptides in regulation of physiology and behavior. Prog Neurobiol. 92 1: 42–104. 20447440 10.1016/j.pneurobio.2010.04.010

[msy160-B80] Okubo K, Nagahama Y. 2008 Structural and functional evolution of gonadotropin-releasing hormone in vertebrates. Acta Physiol (Oxf). 193 1: 3–15. 18284378 10.1111/j.1748-1716.2008.01832.x

[msy160-B81] Perea-Atienza E, Gavilan B, Chiodin M, Abril JF, Hoff KJ, Poustka AJ, Martinez P. 2015 The nervous system of Xenacoelomorpha: a genomic perspective. J Exp Biol. 218(Pt 4): 618–628. 25696825 10.1242/jeb.110379

[msy160-B82] Petersen TN, Brunak S, von Heijne G, Nielsen H. 2011 SignalP 4.0: discriminating signal peptides from transmembrane regions. Nat Methods. 8 10: 785–786. 21959131 10.1038/nmeth.1701

[msy160-B83] Peymen K, Watteyne J, Frooninckx L, Schoofs L, Beets I. 2014 The FMRFamide-like peptide family in nematodes. Front Endocrinol. 5:90. 10.3389/fendo.2014.00090 PMC4058706 24982652

[msy160-B84] Philippe H, Brinkmann H, Copley RR, Moroz LL, Nakano H, Poustka AJ, Wallberg A, Peterson KJ, Telford MJ. 2011 Acoelomorph flatworms are deuterostomes related to *Xenoturbella*. Nature 470 7333: 255–258. 21307940 10.1038/nature09676 PMC4025995

[msy160-B85] Pierce SB, Costa M, Wisotzkey R, Devadhar S, Homburger SA, Buchman AR, Ferguson KC, Heller J, Platt DM, Pasquinelli AA, et al 2001 Regulation of DAF-2 receptor signaling by human insulin and ins-1, a member of the unusually large and diverse *C. elegans* insulin gene family. Genes Dev. 15 6: 672–686. 11274053 10.1101/gad.867301 PMC312654

[msy160-B86] Pratt GE, Farnsworth DE, Fok KF, Siegel NR, McCormack AL, Shabanowitz J, Hunt DF, Feyereisen R. 1991 Identity of a second type of allatostatin from cockroach brains: an octadecapeptide amide with a tyrosine-rich address sequence. Proc Natl Acad Sci U S A. 88 6: 2412–2416. 2006179 10.1073/pnas.88.6.2412 PMC51242

[msy160-B87] Price MN, Dehal PS, Arkin AP. 2010 FastTree 2—approximately maximum-likelihood trees for large alignments. PLoS One 5 3: e9490. 20224823 10.1371/journal.pone.0009490 PMC2835736

[msy160-B88] Raikova OI, Meyer-Wachsmuth I, Jondelius U. 2016 The plastic nervous system of Nemertodermatida. Org Divers Evol. 16 1: 85–104.

[msy160-B89] Raikova OI, Reuter M, Gustafsson MK, Maule AG, Halton DW, Jondelius U. 2004 Basiepidermal nervous system in *Nemertoderma westbladi* (Nemertodermatida): GYIRFamide immunoreactivity. Zoology 107 1: 75–86. 16351929 10.1016/j.zool.2003.12.002

[msy160-B90] Raikova OI, Reuter M, Jondelius U, Gustafsson MKS. 2000a An immunocytochemical and ultrastructural study of the nervous and muscular systems of *Xenoturbella westbladi* (Bilateria inc. sed.). Zoomorphology 120 2: 107–118.

[msy160-B91] Raikova OI, Reuter M, Jondelius U, Gustafsson MKS. 2000b The brain of the Nemertodermatida (Platyhelminthes) as revealed by anti-5HT and anti-FMRFamide immunostainings. Tissue Cell 32 5: 358–365. 11201275 10.1054/tice.2000.0121

[msy160-B92] Rappsilber J, Mann M, Ishihama Y. 2007 Protocol for micro-purification, enrichment, pre-fractionation and storage of peptides for proteomics using StageTips. Nat Protoc. 2 8: 1896–1906. 17703201 10.1038/nprot.2007.261

[msy160-B93] Reuter M, Raikova OI, Jondelius U, Gustafsson MKS, Maule AG, Halton DW. 2001 Organisation of the nervous system in the Acoela: an immunocytochemical study. Tissue Cell 33: 119–128. 11392663 10.1054/tice.2000.0134

[msy160-B94] Robertson HE, Lapraz F, Egger B, Telford MJ, Schiffer PH. 2017 The mitochondrial genomes of the acoelomorph worms *Paratomella rubra*, *Isodiametra pulchra* and *Archaphanostoma ylvae*. Sci Rep. 7 1: 1847. 28500313 10.1038/s41598-017-01608-4 PMC5431833

[msy160-B95] Roch GJ, Sherwood NM. 2014 Glycoprotein hormones and their receptors emerged at the origin of metazoans. Genome Biol Evol. 6 6: 1466–1479. 24904013 10.1093/gbe/evu118 PMC4079206

[msy160-B96] Roch GJ, Tello JA, Sherwood NM. 2014 At the transition from invertebrates to vertebrates, a novel GnRH-like peptide emerges in amphioxus. Mol Biol Evol. 31 4: 765–778. 24361996 10.1093/molbev/mst269 PMC3969558

[msy160-B97] Rouse GW, Wilson NG, Carvajal JI, Vrijenhoek RC. 2016 New deep-sea species of *Xenoturbella* and the position of Xenacoelomorpha. Nature 530 7588: 94–97. 26842060 10.1038/nature16545

[msy160-B98] Ruiz-Trillo I, Riutort M, Littlewood DT, Herniou EA, Baguna J. 1999 Acoel flatworms: earliest extant bilaterian Metazoans, not members of Platyhelminthes. Science 283 5409: 1919–1923. 10082465 10.1126/science.283.5409.1919

[msy160-B99] Sakurai T, Amemiya A, Ishii M, Matsuzaki I, Chemelli RM, Tanaka H, Williams SC, Richardson JA, Kozlowski GP, Wilson S, et al 1998 Orexins and orexin receptors: a family of hypothalamic neuropeptides and G protein-coupled receptors that regulate feeding behavior. Cell 92:573–585. 9491897 10.1016/s0092-8674(00)80949-6

[msy160-B100] Salzet M, Bulet P, Wattez C, Malecha J. 1994 FMRFamide-related peptides in the sex segmental ganglia of the Pharyngobdellid leech *Erpobdella octoculata*—Identification and involvement in the control of hydric balance. Eur J Biochem. 221 1: 269–275. 8168516 10.1111/j.1432-1033.1994.tb18738.x

[msy160-B101] Schank JR, Ryabinin AE, Giardino WJ, Ciccocioppo R, Heilig M. 2012 Stress-related neuropeptides and addictive behaviors: beyond the usual suspects. Neuron 76 1: 192–208. 23040815 10.1016/j.neuron.2012.09.026 PMC3495179

[msy160-B102] Semmens DC, Beets I, Rowe ML, Blowes LM, Oliveri P, Elphick MR. 2015 Discovery of sea urchin NGFFFamide receptor unites a bilaterian neuropeptide family. Open Biol. 5 4: 150030. 25904544 10.1098/rsob.150030 PMC4422128

[msy160-B103] Semmens DC, Dane RE, Pancholi MR, Slade SE, Scrivens JH, Elphick MR. 2013 Discovery of a novel neurophysin-associated neuropeptide that triggers cardiac stomach contraction and retraction in starfish. J Exp Biol. 216(Pt 21): 4047–4053. 23913946 10.1242/jeb.092171

[msy160-B104] Semmens DC, Mirabeau O, Moghul I, Pancholi MR, Wurm Y, Elphick MR. 2016 Transcriptomic identification of starfish neuropeptide precursors yields new insights into neuropeptide evolution. Open Biol. 6 2: 150224. 26865025 10.1098/rsob.150224 PMC4772807

[msy160-B105] Semmler H, Chiodin M, Bailly X, Martinez P, Wanninger A. 2010 Steps towards a centralized nervous system in basal bilaterians: insights from neurogenesis of the acoel *Symsagittifera roscoffensis*. Dev Growth Differ. 52 8: 701–713. 20874714 10.1111/j.1440-169X.2010.01207.x

[msy160-B106] Senatore A, Reese TS, Smith CL. 2017 Neuropeptidergic integration of behavior in *Trichoplax adhaerens*, an animal without synapses. J Exp Biol. 220(Pt 18): 3381–3390. 28931721 10.1242/jeb.162396 PMC5612019

[msy160-B107] Shinzato C, Shoguchi E, Kawashima T, Hamada M, Hisata K, Tanaka M, Fujie M, Fujiwara M, Koyanagi R, Ikuta T, et al 2011 Using the *Acropora digitifera* genome to understand coral responses to environmental change. Nature 476 7360: 320–323. 21785439 10.1038/nature10249

[msy160-B108] Southey BR, Amare A, Zimmerman TA, Rodriguez-Zas SL, Sweedler JV. 2006 NeuroPred: a tool to predict cleavage sites in neuropeptide precursors and provide the masses of the resulting peptides. Nucleic Acids Res. 34(Web Server issue): W267–W272. 16845008 10.1093/nar/gkl161 PMC1538825

[msy160-B109] Srivastava M, Mazza-Curll KL, van Wolfswinkel JC, Reddien PW. 2014 Whole-body acoel regeneration is controlled by Wnt and Bmp-Admp signaling. Curr Biol. 24 10: 1107–1113. 24768051 10.1016/j.cub.2014.03.042

[msy160-B110] Stach T. 2015 Xenoturbella In: Schmidt-Rhaesa A, Harzsch S, Purschke G, editors. Structure and evolution of invertebrate nervous systems. Oxford (UK): Oxford University Press p. 62–66.

[msy160-B111] Stach T, Dupont S, Israelson O, Fauville G, Nakano H, Kanneby T, Thorndyke M. 2005 Nerve cells of *Xenoturbella bocki* (phylum uncertain) and *Harrimania kupfferi* (Enteropneusta) are positively immunoreactive to antibodies raised against echinoderm neuropeptides. J Mar Biol Assoc UK. 85 06: 1519–1524.

[msy160-B112] Stamatakis A. 2014 RAxML version 8: a tool for phylogenetic analysis and post-analysis of large phylogenies. Bioinformatics 30 9: 1312–1313. 24451623 10.1093/bioinformatics/btu033 PMC3998144

[msy160-B113] Steele RE, Lieu P, Mai NH, Shenk MA, Sarras MP. 1996 Response to insulin and the expression pattern of a gene encoding an insulin receptor homologue suggest a role for an insulin-like molecule in regulating growth and patterning in *Hydra*. Dev Genes Evol. 206 4: 247–259. 24173564 10.1007/s004270050050

[msy160-B114] Stevens RC, Cherezov V, Katritch V, Abagyan R, Kuhn P, Rosen H, Wuthrich K. 2013 The GPCR Network: a large-scale collaboration to determine human GPCR structure and function. Nat Rev Drug Discov. 12 1: 25–34. 23237917 10.1038/nrd3859 PMC3723354

[msy160-B115] Stewart MJ, Favrel P, Rotgans BA, Wang T, Zhao M, Sohail M, O’Connor WA, Elizur A, Henry J, Cummins SF. 2014 Neuropeptides encoded by the genomes of the Akoya pearl oyster *Pinctata fucata* and Pacific oyster *Crassostrea gigas*: a bioinformatic and peptidomic survey. BMC Genomics 15 1: 840. 25277059 10.1186/1471-2164-15-840 PMC4200219

[msy160-B116] Suwansa-Ard S, Chaiyamoon A, Talarovicova A, Tinikul R, Tinikul Y, Poomtong T, Elphick MR, Cummins SF, Sobhon P. 2018 Transcriptomic discovery and comparative analysis of neuropeptide precursors in sea cucumbers (Holothuroidea). Peptides 99:231–240. 29054501 10.1016/j.peptides.2017.10.008

[msy160-B117] Telford MJ, Copley RR. 2016 Zoology: war of the worms. Curr Biol. 26 8: R335–R337. 27115693 10.1016/j.cub.2016.03.015

[msy160-B118] Terhzaz S, Teets NM, Cabrero P, Henderson L, Ritchie MG, Nachman RJ, Dow JA, Denlinger DL, Davies SA. 2015 Insect capa neuropeptides impact desiccation and cold tolerance. Proc Natl Acad Sci U S A. 112 9: 2882–2887. 25730885 10.1073/pnas.1501518112 PMC4352776

[msy160-B119] Tessmar-Raible K, Raible F, Christodoulou F, Guy K, Rembold M, Hausen H, Arendt D. 2007 Conserved sensory-neurosecretory cell types in annelid and fish forebrain: insights into hypothalamus evolution. Cell 129 7: 1389–1400. 17604726 10.1016/j.cell.2007.04.041

[msy160-B120] Thiel D, Bauknecht P, Jékely G, Hejnol A. 2017 An ancient FMRFamide-related peptide-receptor pair induces defence behaviour in a brachiopod larva. Open Biol. 7:170136. 28835571 10.1098/rsob.170136 PMC5577450

[msy160-B121] Tian S, Zandawala M, Beets I, Baytemur E, Slade SE, Scrivens JH, Elphick MR. 2016 Urbilaterian origin of paralogous GnRH and corazonin neuropeptide signalling pathways. Sci Rep. 6:28788. 27350121 10.1038/srep28788 PMC4923880

[msy160-B122] Tosches MA, Arendt D. 2013 The bilaterian forebrain: an evolutionary chimaera. Curr Opin Neurobiol. 23 6: 1080–1089. 24080363 10.1016/j.conb.2013.09.005

[msy160-B123] Van Sinay E, Mirabeau O, Depuydt G, Van Hiel MB, Peymen K, Watteyne J, Zels S, Schoofs L, Beets I. 2017 Evolutionarily conserved TRH neuropeptide pathway regulates growth in *Caenorhabditis elegans*. Proc Natl Acad Sci U S A. 114 20: E4065–E4074. 28461507 10.1073/pnas.1617392114 PMC5441806

[msy160-B124] Veenstra JA. 2010 Neurohormones and neuropeptides encoded by the genome of *Lottia gigantea*, with reference to other mollusks and insects. Gen Comp Endocrinol. 167 1: 86–103. 20171220 10.1016/j.ygcen.2010.02.010

[msy160-B125] Veenstra JA. 2011 Neuropeptide evolution: neurohormones and neuropeptides predicted from the genomes of *Capitella teleta* and *Helobdella robusta*. Gen Comp Endocrinol. 171 2: 160–175. 21241702 10.1016/j.ygcen.2011.01.005

[msy160-B126] Vilim FS, Sasaki K, Rybak J, Alexeeva V, Cropper EC, Jing J, Orekhova IV, Brezina V, Price D, Romanova EV, et al 2010 Distinct mechanisms produce functionally complementary actions of neuropeptides that are structurally related but derived from different precursors. J Neurosci. 30 1: 131–147. 20053896 10.1523/JNEUROSCI.3282-09.2010 PMC2826173

[msy160-B127] Walker RJ, Papaioannou S, Holden-Dye L. 2009 A review of FMRFamide- and RFamide-like peptides in metazoa. Invert Neurosci. 9(3–4): 111–153. 20191373 10.1007/s10158-010-0097-7

[msy160-B128] Wang H, Girskis K, Janssen T, Chan JP, Dasgupta K, Knowles JA, Schoofs L, Sieburth D. 2013 Neuropeptide secreted from a pacemaker activates neurons to control a rhythmic behavior. Curr Biol. 23 9: 746–754. 23583549 10.1016/j.cub.2013.03.049 PMC3651789

[msy160-B129] Wegener C, Gorbashov A. 2008 Molecular evolution of neuropeptides in the genus *Drosophila*. Genome Biol. 9 8: R131. 18717992 10.1186/gb-2008-9-8-r131 PMC2575521

[msy160-B130] Williams EA, Conzelmann M, Jékely G. 2015 Myoinhibitory peptide regulates feeding in the marine annelid *Platynereis*. Front Zool. 12 1: 1. 25628752 10.1186/s12983-014-0093-6 PMC4307165

[msy160-B131] Williams EA, Veraszto C, Jasek S, Conzelmann M, Shahidi R, Bauknecht P, Mirabeau O, Jékely G. 2017 Synaptic and peptidergic connectome of a neurosecretory center in the annelid brain. Elife 6:e26349. 29199953 10.7554/eLife.26349 PMC5747525

[msy160-B132] Woodhead AP, Khan MA, Stay B, Tobe SS. 1994 Two new allatostatins from the brains of *Diploptera punctata*. Insect Biochem Mol Biol. 24 3: 257–263. 8019575 10.1016/0965-1748(94)90005-1

[msy160-B133] Woodhead AP, Stay B, Seidel SL, Khan MA, Tobe SS. 1989 Primary structure of four allatostatins: neuropeptide inhibitors of juvenile hormone synthesis. Proc Natl Acad Sci U S A. 86 15: 5997–6001. 2762309 10.1073/pnas.86.15.5997 PMC297759

[msy160-B134] Zandawala M, Tian S, Elphick MR. 2018 The evolution and nomenclature of GnRH-type and corazonin-type neuropeptide signaling systems. Gen Comp Endocrinol. 264: 64–77. 10.1016/j.ygcen.2017.06.007 28622978

[msy160-B135] Zatylny-Gaudin C, Cornet V, Leduc A, Zanuttini B, Corre E, Le Corguille G, Bernay B, Garderes J, Kraut A, Coute Y, et al 2016 Neuropeptidome of the cephalopod *Sepia officinalis*: identification, tissue mapping, and expression pattern of neuropeptides and neurohormones during egg laying. J Proteome Res. 15 1: 48–67. 26632866 10.1021/acs.jproteome.5b00463

[msy160-B136] Zhang W, Yan Z, Li B, Jan LY, Jan YN. 2014 Identification of motor neurons and a mechanosensitive sensory neuron in the defecation circuitry of *Drosophila* larvae. Elife 3:e03293. 10.7554/eLife.03293 PMC4244571 25358089

[msy160-B137] Zhang YZ, Shen HB. 2017 Signal-3L 2.0: a hierarchical mixture model for enhancing protein signal peptide prediction by incorporating residue-domain cross-level features. J Chem Inf Model. 57 4: 988–999. 28298081 10.1021/acs.jcim.6b00484

