# Supplementary material for: Xenacoelomorph Neuropeptidomes Reveal a Major Expansion of Neuropeptide Systems during Early Bilaterian Evolution
Source: Mol Biol Evol. 2018 Aug 24;35(10):2528–43. doi: 10.1093/molbev/msy160 (PMC6188537; doi:10.1093/molbev/msy160)
Supplement: Supplementary Data [file msy160_supp.zip › Supplementary_Figure01-03.pdf]

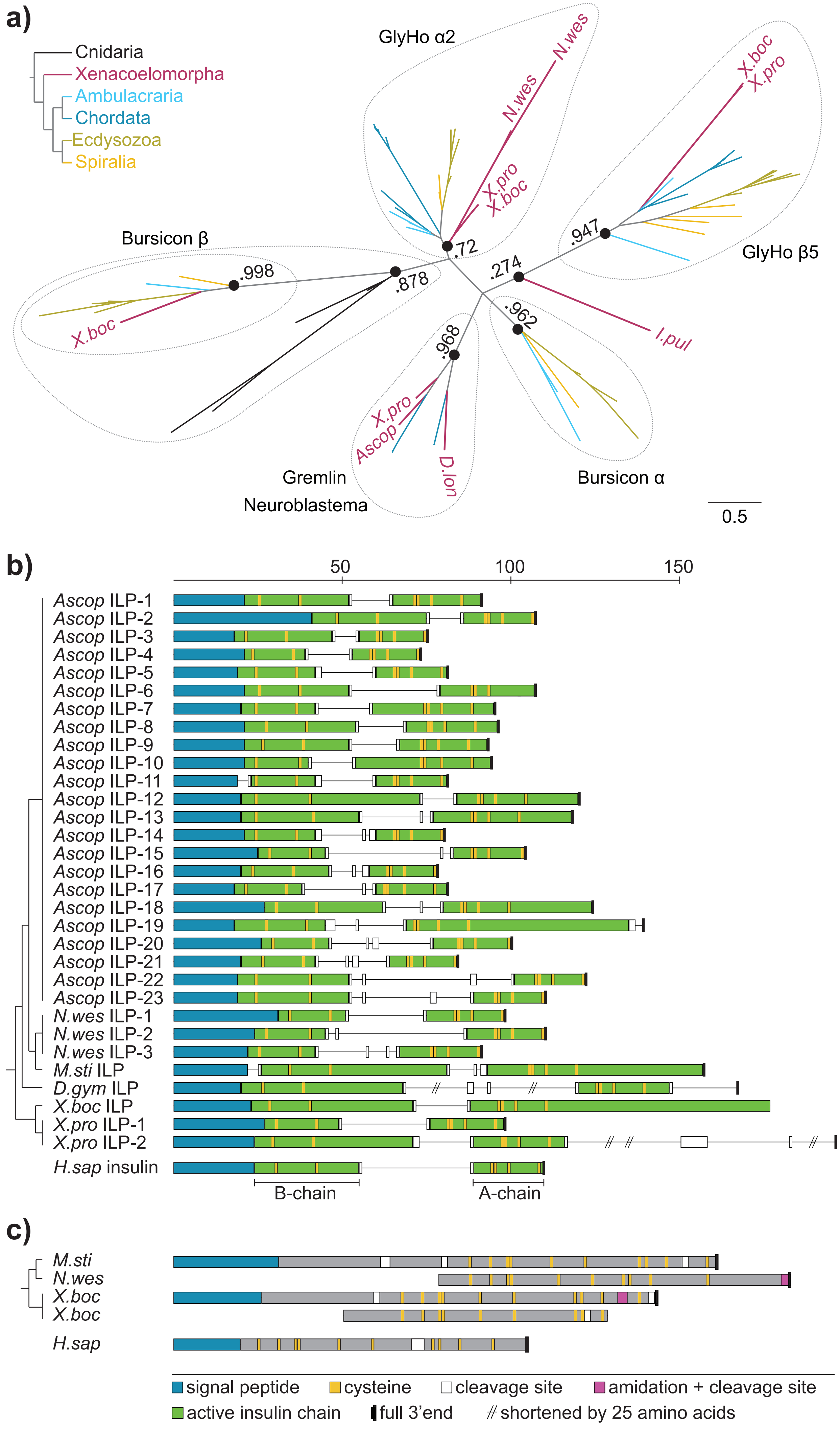

a)
Cnidaria
Xenacoelomorpha
Ambulacraria
Chordata
Ecdysozoa
Spiralia
GlyHo α2
N.wes
N.wes
X.pro
X.boc
X.boc
X.pro
GlyHo β5
.947
.72
.274
Bursicon β
.998
.878
X.boc
.962
I.pul
.968
X.pro
Ascop
D.lon
Gremlin
Neuroblastema
Bursicon α
0.5
b)
50
100
150
Ascop ILP-1
Ascop ILP-2
Ascop ILP-3
Ascop ILP-4
Ascop ILP-5
Ascop ILP-6
Ascop ILP-7
Ascop ILP-8
Ascop ILP-9
Ascop ILP-10
Ascop ILP-11
Ascop ILP-12
Ascop ILP-13
Ascop ILP-14
Ascop ILP-15
Ascop ILP-16
Ascop ILP-17
Ascop ILP-18
Ascop ILP-19
Ascop ILP-20
Ascop ILP-21
Ascop ILP-22
Ascop ILP-23
N.wes ILP-1
N.wes ILP-2
N.wes ILP-3
M.sti ILP
D.gym ILP
X.boc ILP
X.pro ILP-1
X.pro ILP-2
H.sap insulin
B-chain
A-chain
c)
M.sti
N.wes
X.boc
X.boc
H.sap
signal peptide
cysteine
cleavage site
amidation + cleavage site
active insulin chain
full 3'end
shortened by 25 amino acids

Supplementary Figure 1: Conserved metazoan neuropeptides of xenacoelomorphs. **a)** Phylogenetic comparison of glycoprotein hormone related peptides. Color coding of tree branches is shown on the upper left: Magenta = xenacoelomorphs, blue = chordates, light blue = ambulacrarians, olive = ecdysozoans, yellow = spiralians, black = cnidarians. Support values are indicated as SH-like local support. Scale bar on lower right side indicates amino acid substitution rate per site. **b)** Precursor structures of xenacoelomorph insulin-like peptides and human insulin (genbank: P01308.1). Scale bar on top indicates length of precursors in number of amino acids (for b and c). **c)** Precursor structure of xenacoelomorph prokineticin related peptides and human insulin (genbank: NP_115790.1). Ascop = *Ascoparia* spec., D.gym = *Diopisthoporus gymnopharyngeus*, D.lon = *Diopisthoporus longitubus*, GlyHo = glycoprotein hormone, H.sap = *Homo sapiens*, ILP = insulin-like peptide*,* I.pul = *Isodiametra pulchra,* M.sti = *Meara stichopi*, N.wes = *Nemertoderma westbladi*, X.boc = *Xenoturbella bocki*, X.pro = *Xenoturbella profunda*.

Accession numbers for sequences used for Supplementary Figure 1a:
[ELT88681.1 Capitella teleta] [ELU07048.1 Capitella teleta] [XP 970934 Tribolium castaneum] [XP 011441100.1 Crassostrea gigas] [XP 011848532.1 Mandrillus leucophaeus] [XP 013088838.1 Biomphalaria glabrata] [XP 798219 Strongylocentrotus purpuratus] [XP 001863304 Culex quinquefasciatus] [XP 014797396.1 Calidris pugnax] [KFZ68810.1 Podiceps cristatus] [KQK77418.1 Amazona aestiva] [XP 003485714 Bombus impatiens] [GPHB5 Homo sapiens] [EFN71945 Camponotus floridanus] [ABO20870 Musca domestica] [ABX55995 Carcinus maenas] [NP 001103719 Strongylocentrotus purpuratus] [ALJ99968.1 Asterias rubens] [CAR94630 Branchiostoma lanceolatum] [BAH57330 Branchiostoma belcheri] [XP 002610667 Branchiostoma floridae] [CAR94703 Strongylocentrotus purpuratus] [CAR94705 Saccoglossus kowalevskii] [CAR94704 Strongylocentrotus purpuratus] [XP 001377718 Monodelphis domestica] [C6SUS5 Anolis carolinensis] [XP 001343437 Danio rerio] [EGI61460 Acromyrmex echinatior] [XP 002427818 Pediculus humanus] [NP 001124375 Bombyx mori] [XP 002065223 Drosophila willistoni] [ACC99601 Dermacentor variabilis] [NP 001191641.1 Aplysia californica] [XP 013094496.1 Biomphalaria glabrata] [A0RZD4 Tribolium castaneum] [K7JAJ6 Nasonia vitripennis] [KFV46949.1 Gavia stellata] [XP 012719142.1 Fundulus heteroclitus] [NP 990309.1 Gallus gallus] [C6SUQ7 Pediculus humanus] [ALJ99969.1 Asterias rubens] [XP 011441101.1 Crassostrea gigas] [NP 001180095 Bos taurus] [XP 003122599 Sus scrofa] [XP 002755562 Callithrix jacchus] [NP 569720 Mus musculus] [XP 003419654 Loxodonta africana] [XP 001371214 Monodelphis domestica] [XP 003229755 Anolis carolinensis] [NP 001015386 Drosophila melanogaster] [C6SUR3 Bombyx mori] [Q4S0U3 Tetraodon nigroviridis] [D0R090 Branchiostoma lanceolatum] [C6SUR8 Strongylocentrotus purpuratus] [B3XYE2 Bombyx mori] [P41271.2 Homo sapiens] [EFX79541.1 Daphnia pulex] [XP 015784790.1 Tetranychus urticae] [NP 001164244.1 Tribolium castaneum] [XP 015792016.1 Tetranychus urticae] [ABX55997.1 Daphnia arenata] [XP 001636257.1 Nematostella vectensis] [XP 001629625.1 Nematostella vectensis] [KXJ29715.1 Exaiptasia pallida] [XP 015759821.1 Acropora digitifera] [Platynereis dumerilii: bursicon alpha, bursicon beta, glycoprotein hormone beta from Conzelmann et al. 2013: The neuropeptide complement of the marine annelid Platynereis dumerilii. BMC Genomics - supporting material]

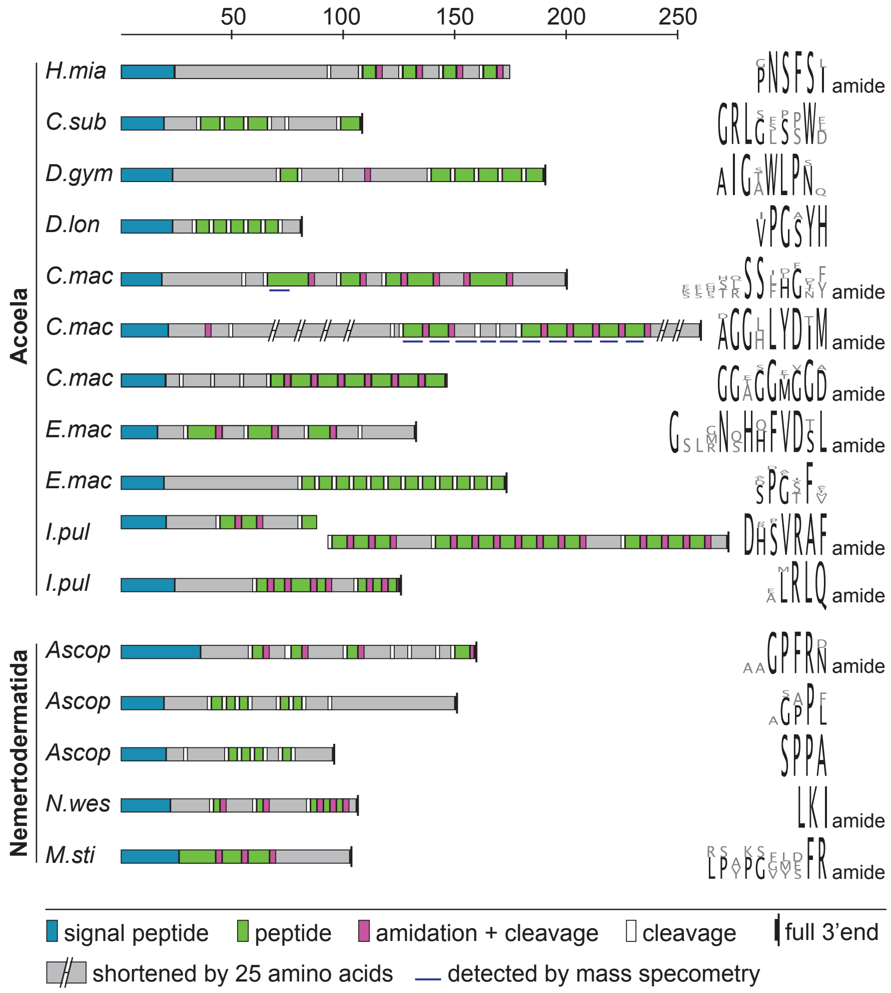

Supplementary Figure 2: Precursor structure and peptide sequence logo representations of xenacoelomorph-specific multi-copy peptides. Scale bar on top indicates length of precursors in number of amino acids. Ascop = *Ascoparia* spec., C.sub = *Childia submaculatum*, C.mac = *Convolutriloba macropyga*, D.gym = *Diopisthoporus gymnopharyngeus*, D.lon = *Diopisthoporus longitubus*, E.mac = *Eumecynostomum macrobursalium*, H.mia = *Hofstenia miamia*, I.pul = *Isodiametra pulchra*, M.sti = *Meara stichopi*, N.wes = *Nemertoderma westbladi*.

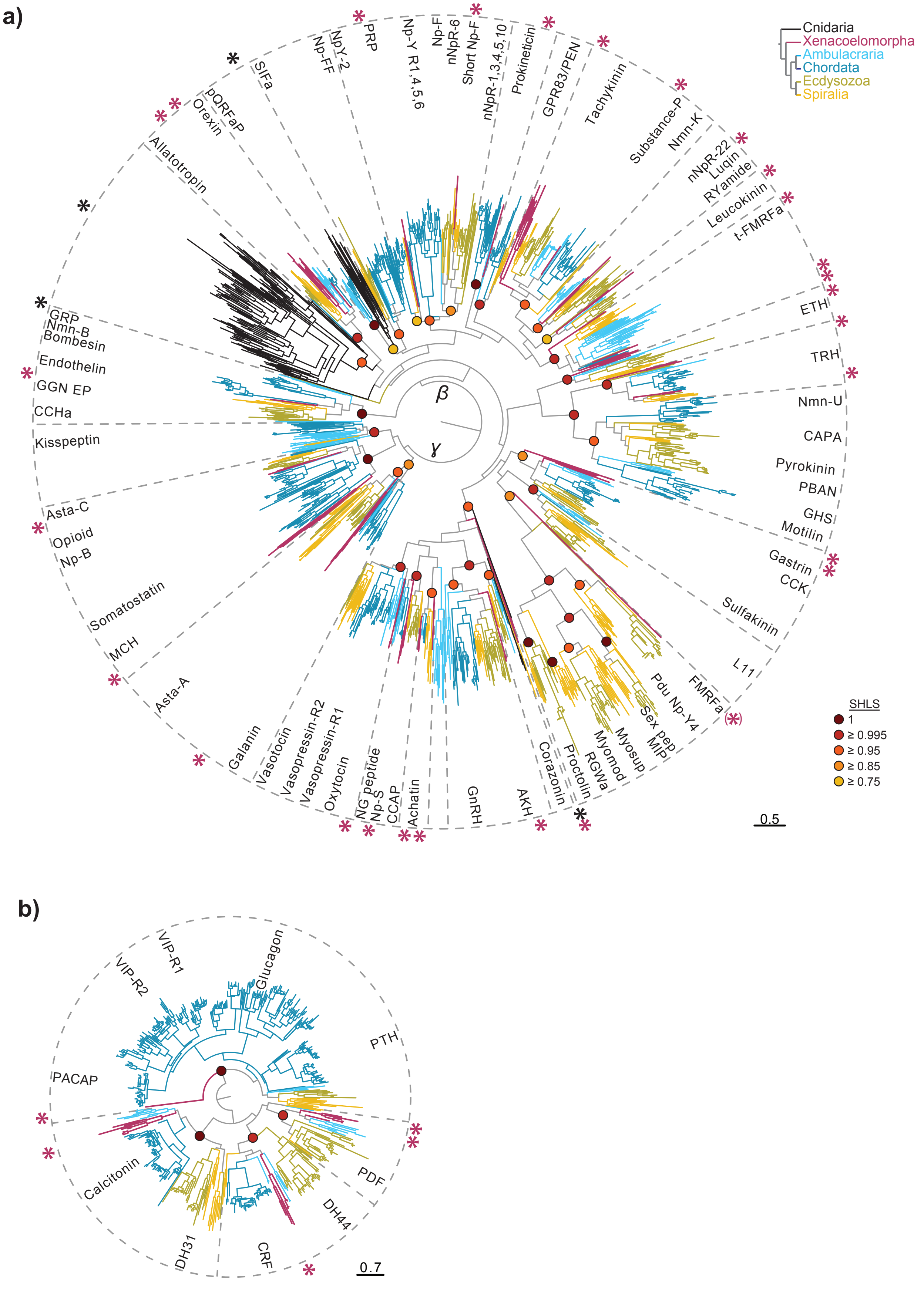
a)
b)
Cnidaria
Xenacoelomorpha
Ambulacraria
Chordata
Ecdysozoa
Spiralia
SHLS
1
≥ 0.995
≥ 0.95
≥ 0.85
≥ 0.75
0.5
0.7
β
γ
Allatotropin
Orexin
pQRFaP
SIFa
NpY-2
Np-FF
PRP
Np-Y R1,4,5,6
Np-F
nNpR-6
Short Np-F
nNpR-1,3,4,5,10
Prokineticin
GPR83/PEN
Tachykinin
Substance-P
Nmn-K
nNpR-22
Luqin
RYamide
Leucokinin
t-FMRFa
ETH
TRH
Nmn-U
CAPA
Pyrokinin
PBAN
GHS
Motilin
Gastrin
CCK
Sulfakinin
L11
FMRFa
Pdu Np-Y4
Sex pep
MIP
Myosup
Myomod
RGWa
Proctolin
Corazonin
AKH
GnRH
Achatin
CCAP
Np-S
NG peptide
Oxytocin
Vasopressin-R1
Vasopressin-R2
Vasotocin
Galanin
Asta-A
MCH
Somatostatin
Np-B
Opioid
Asta-C
Kisspeptin
CCHa
GGN EP
Endothelin
Bombesin
Nmn-B
GRP
VIP-R1
VIP-R2
Glucagon
PTH
PACAP
Calcitonin
PDF
DH44
CRF
DH31

Supplementary Figure 3: Phylogenetic analysis of neuropeptide GPCRs using FastTree. a) Phylogenetic tree of rhodopsin type neuropeptide GPCRs. Rhodopsin *beta* GPCRs are rooted against rhodopsin *gamma* GPCRs. SH-like support values of the corresponding nodes are represented as red-to-yellow circles as shown on the lower right. Scale bar indicates amino acids substitution rate per site. Magenta asterisks indicate the position of xenacoelomorph sequences, while black asterisks indicate the position of cnidarian sequences. Dashed lines depict orthologous receptor types of different animal groups. Color coding of tree branches is shown on the upper right: Magenta = xenacoelomorphs, blue = chordates, light blue = ambulacrarians, olive = ecdysozoans, yellow = spiralians, black = cnidarians. **b)** Phylogenetic tree of secretin type neuropeptide GPCRs. SH-like support and taxa color coding are depicted as in Supplementary Figure 3a. Abbreviations: *a* = amide, *AKH* = adipokinetic hormone, *Asta* = allatostatin, *CCAP* = crustacean cardioaccelatory peptide, *CCK* = cholecystokinin, *CRF* = corticotropin releasing factor, *DH* = diuretic hormone, *GGN-EP* = GGN excitatory peptide, *e*- ecdysozoan, *ETH* = ecdysis triggering hormone, *GHS* = growth hormone segretagogue, *GnRH* = gonadotropin releasing hormone, *GRP* = gastrin releasing peptide, *Np* = neuropeptide, *nNpR* = nematode neuropeptide receptor, *Nmn* = neuromedin, *L11* = elevenin, *MCH* = melanin concentrating hormone, *MIP* = myoinhibitory peptide, *Myosup* = myosuppressin, *PACAP* = pituitary adenylate cyclase-activating polypeptide, *PBAN* = pheromone biosynthesis activating neuropeptide, *PDF* = pigment dispersing factor, *Pdu* = *Platynereis dumerilii*, *NG pep* = NG peptide, *pQRFaP* = pyroglutaminated RFamide peptide, *PTH* = parathyroid hormone, *PRP* = prolactin releasing peptide, R receptor, *SHLS* = SH-like support, *t-* = trochozoan, *TRH* = thryotropin releasing hormone, and *VIP* = vasoactive intestinal peptide.
