# Supplementary material for: Xenacoelomorph Neuropeptidomes Reveal a Major Expansion of Neuropeptide Systems during Early Bilaterian Evolution
Source: Mol Biol Evol. 2018 Aug 24;35(10):2528–43. doi: 10.1093/molbev/msy160 (PMC6188537; doi:10.1093/molbev/msy160)
Supplement: Supplementary Data [file msy160_supp.zip › Supplementary_Figure04.pdf]

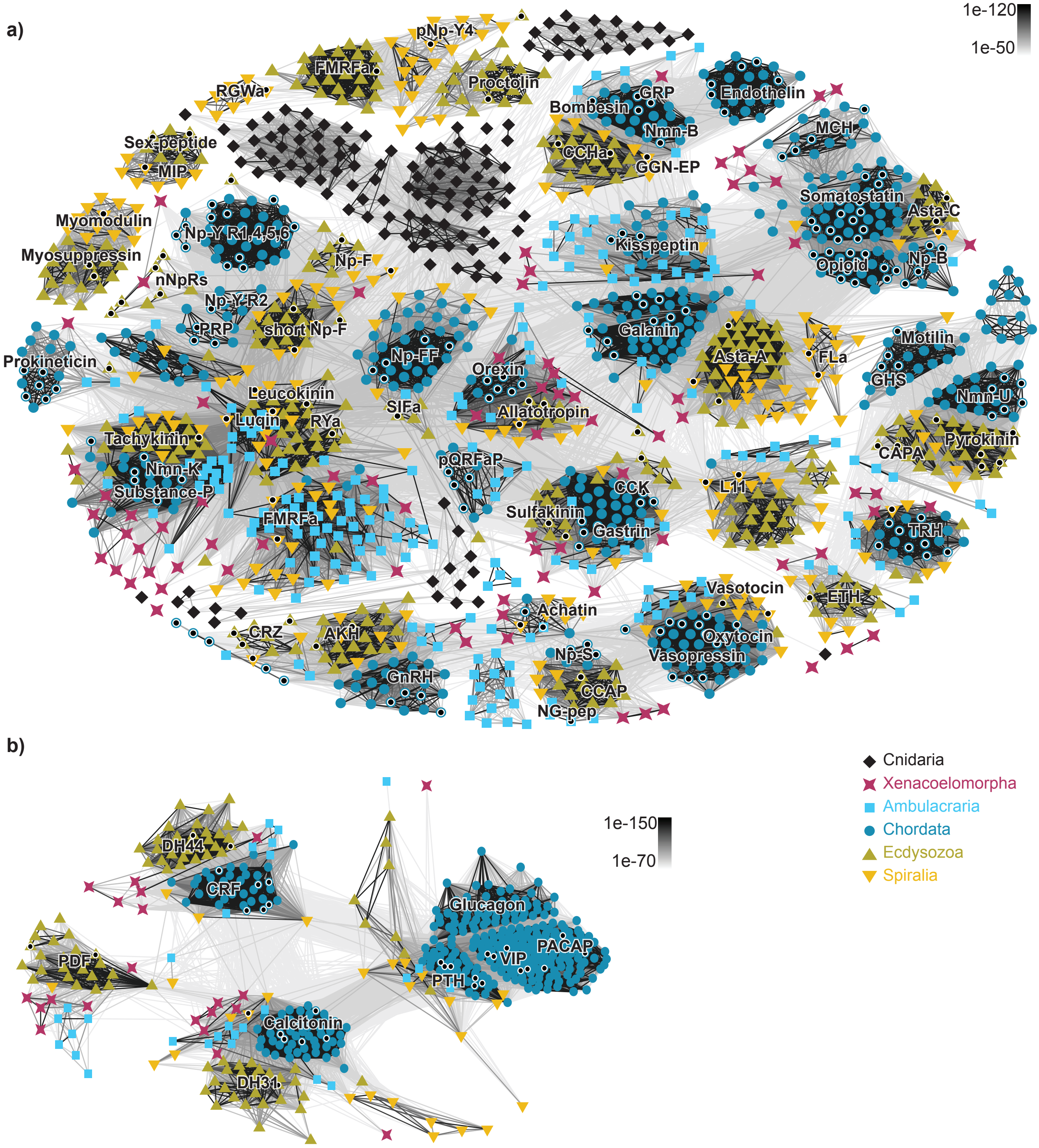

Supplementary Figure 4: Cluster analysis of neuropeptide GPCRs. a) Analysis of rhodopsin type neuropeptide GPCRs. b) Analysis of secretin type neuropeptide GPCRs. Connections are based on blast similarities < 1e-50 (in a) and < 1e-70 (in b). Colour and symbol coding: magenta star = xenacoelomorph, blue filled circle = chordate, light blue square= ambulacrarian, olive upward triangle = ecdysozoan, yellow downwards triangle = spiralian, black diamond = cnidarian. A black dot inside a white circle indicates a receptor for which ligand-receptor activation has been shown before. Abbreviations are listed in Supplementary Figure 3.
