# Supplementary material for: Xenacoelomorph Neuropeptidomes Reveal a Major Expansion of Neuropeptide Systems during Early Bilaterian Evolution
Source: Mol Biol Evol. 2018 Aug 24;35(10):2528–43. doi: 10.1093/molbev/msy160 (PMC6188537; doi:10.1093/molbev/msy160)
Supplement: Supplementary Data [file msy160_supp.zip › Supplementary_neuropeptide_precursors.pdf]

Predicted xenacoelomorph preproneuropeptides

Signal peptide
[K/R] basic cleavage site
[K/R] redundant cleavage site (NeuroPred prediction – only in calcitonins and insulins indicated)
G[K/R] C-terminal amidation + cleavage site
detected by mass spectrometry
Cysteine
*** full 3' end**

Note: Bioinformatic prediction of C-terminal amidation was generally confirmed by mass spectrometry (ms) if the according peptide has been detected. All methionine residues in peptides that were detected by ms showed evidence for oxidation, unless otherwise stated. Posttranslational pyroglutamic acid formation is indicated in the heading of sequences (Cmac_822.1 and Hmia_98008391).

Ascoparia sp.

>Asco_19853.0_t1 LPFamide
MRLLLLISGLIVLVEDAIRVQASDGALSQSEDNNSQISNDHPNVEKKAAFDIPFGKKFAFDMPFGKRLPFDMMPDYKAAFHMPFGKRAAFELPFGKKAAFELPFGKKAAFELPFGKKAAFELPFGKRAA
FELPFGKKAAFELPFGKRDEGGIVDKRREKRSVRSMLDEMDKRGKESTKTRKIGDAWGMPFGKRFAYELPFGKRDNSLEETNKRNDFNDPFGMRMSMFDMPFGKRSLDGDDWFNRLAANHPKKAVFDLP
FGKREGAVVFEGKIEEKIEAEAF*

>Asco_16584.0_t1 WDLamide
MELWCTNIKVYLFVSAVILGYCVAYSDQSISKRPLNWSLGKRSSDWTDVKRGLNWSLGKRGLNWSLGKRGLNWDLGKRGLNWDLGKRSSIPSSERYSSQEGEFVDEDHIADKAEEICLKNSKKLCGITL
LNDGSVVPYCEEKCRYMIEER*

>Asco_11717.0_t2_t1 ELamide
MRYVMLLGIVLYATVLMDYSTGEDNDAYSKHKRAFISELGKRAYISELGKRGYISELGKRAYISELGKRGYISELGKRAYISELGKRDKNYDNEPDRRAFISELGRR*

>Asco_16126.0_t4_t5 SFYNamide
MTTRYMWLSSISAFLIVILLIEISSSLELQPSQGLPSDISQDLRDDIERFEAEHGIDLDELTRKRSFYNGKRSFYNGKRSFYNGKRSFYNGK*

>Asco_6783.0_t1 LRIGamide
MNVNCTFGLVVFVVVFSVCLSLPYRSFDSSESAGSPESLSNESSEVVRRSPLRIGGKRAPLRIGGKRDDEVWSDEYEKRAPLRIGGKRAPLRIGGKRAPLRIGGKRAPLRIGG*

>Asco_11475.0_t1 PFRNamide
MMRYSGILSAVLIVVLCGVAYCYAEEENGECIFDCDEYDPSYQADKRRGPFRNGKRSGEYEMDKRRGPFRNGKRSGLEWVTYLTSDDFGDKRGPFRNGKRILDDEVIGNDEKRRGPFRNSKRSATIETE
FIRDRRSASDFKRAAGPFRDGR*

>Asco_6780.0_t1 GPPL_peptide
MANIVISVATFLALISLVRCLYLNDKSIDAFDNDDLPEVERRAGAPFKRGAPFKRSPPLKRNFADDTRAWLEKRGPPLKRGPPLKREYSEMESDSVKKVVYTSPEWLEAKNQENSINCIVVHNMYNYSL
MKALGFISIILSVSGFNILSPQVKNL*

>Asco_20578.0_t1 SPPA_peptide (alternative signal peptide MKAEIVLIPIVISMIVCTTA)
MKAEIVLIPIVISMIVCTTAARTFYDGYDKRAPPSVVASRSPAWPLDWKRSPPARRSPPAKRSPPAKRFRWNLKRSPPAKRFETDLRHTWCSVFNQCR*

>Ascop_6882.0_t1 Calcitonin-like
MTQTFLFCIIVIGLVSLIPQLSSAYPREADYPIDTARRFVSALDDLYDAVRSEKLEAYMLPKANSQFVRPTGPVKRCSNLSTCLLNRITHGLGRHAPDVGRGSPGKK*

>Asco_20640.0_t1 GnRH-/AKH-like
MKGIQIYASLFCVLIVVNVAFAANGYSGSMNWLPAGKKRAVSDDSKGGLVGPCICRAPNGRRRYFKTVVALDEDLDDTGDF*

>Ascop_18144.0_t1_t2 Vasotocin [5' missing]
MSNLCTLLAVSLFVISAAKACVIVACPRGGKRSIFKRDESIRECRQCGPSLTGKCYGNGLCCGPFGCRFGAEWAKECIEEDYDMRPCWNNAPPCGGNDVSQCAAPGICCDLQKGVCHSNGECGLDWSES
AVKDVKRQEKKATEGKRQEFLPQPSNWKRQEFISRKQ*

>Ascop_Locus_.0_t1 Insulin-like_peptide_1
MARLLLTSILVVVMVATLCRAYDLQCEMDLDERFKTVCGADNTVKFTENWRKRDMSMFLRGFLKRVSLREECCVEGCDNVEIEDRCDFYTA*

>Ascop_Locus_3164.0_t1 Insulin-like_peptide_2
NAEYGDSNRAIKQAVYGIGSNYDARFVLTFVLLVLIVATLAHEPSQISCDEDLTSRINFQCSDGHKMTYPGRSALKFLRAAVFQKREDVNEECCNEGCRKEEVLEHC*

>Ascop_Locus_7774.0_t1 Insulin-like_peptide_3
MKPSIIIFLAVVLVTSEAMRLCGEGLQEGVNKMCSSRGFATNEARSKRYLHRAARSVQHECCSEGCGWEEISEMC*

>Ascop_Locus_7304.0_t3 Insulin-like_peptide_4
MMKFFLLFLVIFSAVLVDKSASYCGVDLVTRWTELCRSKRGASAFIESPVAKRTLHEECCVEGCADEEIAEHC*

>Ascop_Locus_15602.0_t1 Insulin-like_peptide_5
MKLIFVVSVVFVLFASSSANKMWLCGSKLTNARDQACSYNRKRRDVSAASSNTILHRSKRTLSEECCHELCTWEEIYEVCY*

>Ascop_Locus_18694.0_t2 Insulin-like_peptide_6
MARFAAISVLVLVLAATLCYAGTKYCGKQLEEYFFDVCGKTAKYEFHMHNTRRAFPSTNLRSRAASKFLKSFASTYEKRLFATSLETECCEEGCSREEVQDSCEKYT*

>Ascop_Locus_20141.0_t1 Insulin-like_peptide_7
MKMKFFVALCIVALVLSVNANRKLCGGDLDNARSRICYHRGRRSVDATSSAAFLNRNKRATMRGNTRLTTINEECCNETCTDEEIGETCGGSTLT*

>Ascop_Locus_20344.1_t1 Insulin-like_peptide_8
MTRFILTWALVVILAATLCDAGDSVRSCYDTLNSRFYYVCNKDKTVAFSTDYDRRSMATFLRSVGLDKRDNLHEECCTEGCYNEEIMEACDFYAPG*

>Ascop_Locus_20403.1_t1 Insulin-like_peptide_9
MARFILTCVLVVVIAAALCYAVDQRDCGVYLENRFWNICDKDATINLETTFRRSVSMFLRSRGFEKRWSLHEECCIESCSNEEVRADCSYYAP*

>Ascop_Locus_18191.0_t1 Insulin-like_peptide_10
MSRIILLFVLVSVVAVTMVQTDIYCGEFYLEQKFILVCGKKAIKPFDLPMANRRSASNFLTMDERGTYNLWEECCAEGGCSDEEITEGCYDYTH*

>Ascop_Locus_19326.0_t4 Insulin-like_peptide_11
MKVFLVCFLLVLFAYSAESRKLRLCGALLTTAHNTLCGYDRKRRDVSAASSDTILHRTMRSLSEECCNKYCHLEEISEACG*

>Ascop_Locus_4791.0_t1 Insulin-like_peptide_12
MARFLLSLAALALYIVCVAGAPKYCGRHLGKSLEEKYREVCPGHFKAKPFHTGHILRSIDLEDAVYEANPVLRRNVAAAFLSKRDNLWHECCDEGCSNEEIHEVCAFYGEQFEKFKPGGK*

>Ascop_Locus_2505.0_t1 Insulin-like_peptide_13
MVRFVVVACLLVVWLTTVAAHPKYCSSRATDVSLEDKFRKLCDVNMSIIPFGREMRSFEERGRIPDLRAYRSSKAAKFLHLRGNLWHECCVEGCSDHEIVEACGFYGNASEEYHNEWA*

>Ascop_Locus_2617.0_t1 Insulin-like_peptide_14
MKVSIVAVLCILVVVAASADALRLCGERLTETRNQQCGSGRKRRDMSAASSDTMLRRERRNLSEECCNEGCSWEEIFEVC*

>Ascop_Locus_20650.0_t1 Insulin-like_peptide_15
MSTLSVAPPLIVSVICLSLLVSSNAEILCGSDIPKAMAMACAFGKRSGPTRPEGLTRQRVRFPSTLGDNTDTVFDHRLKRQWKGIADYCCDRGCTLDELASAVC*

>Ascop_Locus_12564.0_t1 Insulin-like_peptide_16
MKLIALLCVTAVVCLAVSDAMRICGESLTEQSNGACTHGVRTASDKRSNSFAKRFVKRSASEECCHEGCSWEEIYEGC*

>Ascop_Locus_14352.0_t2 Insulin-like_peptide_17
MKFAVVCFVAVLLAAAEASHYCGVDYENYRFEVCNHHKRDLTDQASDTYLSSNKQKRDMRHECCSGSGCTWNEVHDDCNRG*

>Ascop_Locus_16747.0_t1 Insulin-like_peptide_18
MMCSVNILRVLMLLSVIMASLIVISDAGHYCNIEMDEKWRQVCAGKTRLLPLFYFYYRGMSKREAANFLENEKRVSLVKRSVYHECCDEGCSDEEVNESCEPLSNQENYQEFVDSGGMVARYNR*

>Ascop_Locus_11131.0_t1 Insulin-like_peptide_19
MKVIAACLVVAFAALAYCDDEEQYTMYCGEALQRKIVDTCHLRIQRRRSDTFFRRALPRIHELLWRGMKEECCSDEGGCVDEEVEDKCPIIHFLMPEPIIMVDPIPKPLPTLIVDPVEPIIGKGGENPF
PEPPKAKRHH*

>Ascop_Locus_16610.0_t2 Insulin-like_peptide_20
MKRLCFMLIVSLHVFSFLSDTTVVEAEIICGRDISRALAMACAFGKRSAPTSITTTRRKRRHLDLPPDQTQITTQKRWRGIAHYCCQIGCDINQLAAAVC*

>Ascop_Locus_13690.0_t2 Insulin-like_peptide_21
MNLAFVSVLTLLCVVAIVDADEYYNYCGIDLVRKWYQLCRNKRDSFAEGLSRKRRNVDELVSKRDLHNECCNENCKDEEIAERC*

>Ascop_Locus_11179.0_t3 Insulin-like_peptide_22
MKLALFVCILVLAIAVCGADRKQLCGNEGLATIKEIQSEKCIIKTPHHHHEKKPAKKPAGGEGGEGGEGGAGGEGGEGAGRQGAPRMQKRSADTYLSEMKRGDILEKCCSKGCSVEEIREAC*

>Ascop_Locus_11179.0_t4 Insulin-like_peptide_23
MKLALFVCILVLAIAVCGADRKQLCGNEGLATIKEIQSEKCIIKTPHHHHEKKPAKKPAGGEGGEGAGRQGAPRMQKRSADTYLSEMKRGDILEKCCSKGCSVEEIREAC*

additional *Ascoparia* sp. prepronenuropeptide candidates:

>Asco_16273.0_t6 [3’ missing] (alternative signal peptide MTSHGRTSTLLLVWLSFGFAISKA)
MTSHGRTSTLLLVWLSFGFAISKADAAYSTTELDGAFARRLRAKIDQIIERSQLDELIDNRAIPVKRTTPEWLDELKDDINDAKRAIPVRRAIPVKKAIPVKRVESELDEKRAIPVKKAIPVKREE

>Asco_17603.0_t1
MVNPVVAVIFCISVQSHIISGVLVKPPSGFGFDTFVSSPNGKRMPFPIDDRLTVNGRTPARENGKHLIPEDKLMVTKLAFFPDDRFNVNGRSPANDKRISFPTMIRKRSANGKRAFFPDDRFKVNGKSP
ANGKRDYFPDDRFKVNGRAPANGKRVFSPGERFEVNGRSPANGKLDFFLDDRLDVGRSTTNGNDMQIFNHTIDLTFDELFQLAYKQVENELMKEITNNNDCKADRNHEFVNNRNNNLGFWKIDRAD*

Childia submaculatum

>Csub_c30872_g1_i1 FNMamide
WYQRRVVLPVFFFFVAVALAHYQDPSGSRLSDSFEEYPHLMEFSDEDALEKRAYAFNMGKKIAEDFTEDKKPYAFNMGKKAYAFNMGKKAYAFNMGKKAYAFNMGKKLDEEDFTQDKKSYAFNMGKRPY
AFNMGKRPYAFNMGRR*

>Csub_c355_g1_i1 SFVNamide
MKNSMFFSFVFAFLALTSTISSFRVFAPATRRGILFQKTVARELGKRVGLRTWNWHEDEQEFFKRSFVNGKRSFVNGK*

>Csub_c12842_g1_i1 PSFamide (FxxxFamide)
MNIQYKKTYLCFELFLCITINLSLNSAHLTPQTQIIPHKGLRGEELRAKSELNQGASFRKPTANCQGCGGPFLLGKSQEFADIFNIYLTSTGKTSSSSSWKRFSSSFGKRFTASFGKRFQPSFGKRFRP
SFGKRFDKRLDDLFGQTFKFSIGERLSESKNSTFTPSFGKKDMSKEDIFIPKLRGTLQPRLRRSSQCTHALV*

>Csub_c14012_g1_i4 GRL_peptide (alternative signal peptide MKVAAILVLFMVGLVAVVLCIS)
MKVAAILVLFMVGLVAVVLCISDESSSEGNGLNLDKRGRLGSSPWEKRGRLGFSPWDKRGRLGLSSWDKRGRLTLNKREDGLVSPAQPLLDDDFESKMETKRGRLSLPSWL*

>Csub_c14972_g1_i1 AWD_peptide
MKITSFFIGSILLIITLQVCQYTSGHSIQVPDDIDGPVSLKRAMAWDFHHGGNGKRAIAWDFLKRNDENEENTGRRLNIAWDLVKQASQNEGSPGQKRMAWDFNKRNSQEKRAMGWNFLH*

>Csub_c13095_g1_i1 MRF_peptide (alternative signal peptide MKNIGLIVLAALFVCLQLACCAG)
MKNIGLIVLAALFVCLQLACCAGIDQDGLSRTEYKSDIDKRFRFAPRSNHPQETFTKRDENDELEKRMRFRENDDRWKRMRFISQRRMRFVPEKRMRFIPEKRMRFAPEKRMRFSPHKRMRFAPH*

>Csub_c13564_g1_i1 LWD_peptide
MKCNLVALYLAILFSTLKGVESVPDDQLNEVKGSQEDGHNEAKKLWDYGASTKRHQPDVEENTEGKRLWDYGQSKRLSDNEPSKRLWDYGPAKRFVENEPSKKLWD

>Csub_c12811_g1_i1 amidated_MFGYG_peptide (MxGFG_petide) [3’ missing]
MKFPKIVNVFSTIIFAILFQCILTNGHSLNQELSTGEKYPESKRIFGYTGEMAGHEKRMFGYGGDMAAGKRMFGYGGDMATGKRMFGYGGDMAAGKRMFGYG

>Csub_c28735_g1_i1 LRFamide [5’ missing]
STWGNIRFGKRHFDKAWDAYNVGEPEVKRYLRFGKRFEDPSADKRYLRFGKRYLRFGK*

additional *Childia submaculatum* prepronenuropeptide candidates:

>Csub_c17540_g1_i2 [5’ missing]
AEYMGGEDNGLGKKEGEDSGLGKRDGEDKGLGKREGEDNGVGKREGDDNGVGKKEEEDNGVGKREGEDNEPSKKEEEEDNGVGKREGEDNELGKKDEVEDNGAGKREGDDNVVGKREGEEDNGERLVEG
DKEEACTRERDEKGQEGRVIGNRGDNWTGSDGKITGGEDTNCGIGKQEEKRGEEQENDTCGKRADSAEENGKMERVDNVPGTMANGETLDDSGVIKGNDVKDTHFEVGIKLDEEQLSDTGLY*

>Csub_c17540_g1_i1 [5’ missing]
AEYMGGEDNGLGKKEGEDSGLGKRDGEDKGLGKREGEDNGVGKREGDDNGVGKKEEEDNGVGKREGEDNEPSKKEEEEDNGVGKREGEDNELGKKDEVEDNGAGKREGDDNVVGKREGEEDNGERLVEG
DKEEACTRERDEKGQEGRVIGNRGDNWTGSDGKITGGEDTNCGIGKQEEKRGEEQENDTCGKRADSAEENGKMERVDNVPGTMANGETLDDSGVIKGNDVKDTHFEVGIKLDEEQLSDTGL*

Convolutriloba macropyga

>Cmac_8297.1 amidated_SS_peptide
MKLAPLLFVSICCLSTALSSDQNGAAEDSNLIIFPGDLDVLSTPDLIELLDHSDEVKRSSFHGKFRKRILGSDPFEEHTRSSILGEYGKRGDVDKVEDQDKRTRSSFHGNYGKRSSFHGNWKRSSFHGN
FGKKSSPSLSSLHGTFGKRSSLSSLHGDFGGKRAAWYNKLGHQSSFDEIYGKRDFLGDEIQNTPEESGQLGYLVNVY*

>Cmac_1031.1 AWD_peptide (alternative signal peptide MSSDSMLSSSLSLLFVISCYYCCVSLITC)
MSSDSMLSSSLSLLFVISCYYCCVSLITCEDATGPAAPALIAPEPISLHGGHIPAQLLMKRGVAWDFTKKVPVGKRNLAWDFTRRSSDDTEGKRALAWDFTRRSGDEGKQSMPWDLDGKRNLAWDFTKR
TPVALIPEDKRALAWDFTKKYDSDKRALAWDFTKKSVIPVEIASNSPPYDKRALAWDFTR*

>Cmac_643.1 SSAMHFF_peptide (SSxxxF_peptide)
MSRAANHSGVSKLMFGFCVLVAVTIFVQETTALTIAVPETEQDALLYSEDSSDPTSYDKRSSAMHFFKRESPLTEEELLKRSSPWHYGKRSSPWHYGKRSSPWHYGKRSSAMHFFKRDSNGYPYLDESS
SEGFYA*

>Cmac_3219.1 MHFamide (SSxxxFamide) (alternative signal peptide MICLSILIANCLINLSS)
MICLSILIANCLINLSSQSVVDRDLSSEQFYAASNLVAPPIILKRAHRMHFGKRAPDSSSEDFEQYPSYYLEPLIPEKRSNSMHFGKRASSLWRLEAPQFNSKKEAGSSMHWGR*

>Cmac_1299.1 SSFRSF_peptide (SSxxxF_peptide)

MSGSGSQILASFLNIFLISVVLCVLSALYCSGDYYPTDLDFKRTEDLFESPDLRNNWSSLRGFNRRNWSSFRSFKRSPQRWSSFRSFKRRSTDNPLD*

>Cmac_6993.1 PSFamide (FxxxFamide) (alternative signal peptide MSQVLTFAVTITIAISCFFNFGSAA)
MSQVLTFAVTITIAISCFFNFGSAAIPSLSWVSKDSSSRVSPVSLGLGVPVFMGTSASDPATDIDRRAFMVKRNSVEDPNFTRGLFMSPDASWGNQPVPQWMSVKRLGAKSHFSPSFGKRMTFAPSFGK
RQPSAALFQSGRGQTVGVQGSASEKKRMARKEFYPSFGKRNLVNFMENQAKPFPFQVNRK*
a
>Cmac_1739.1 LWD_peptide
MSTNLLPFVLLSAVLCFSSLVTSRSLESESGGSPVLFLSDPENNHPHLIHSGNNPDKRLWDYQYKRNSGLPVSLGAENPDEKRLWDYQYKRAVGLPEGVATGGDEKRLWDYQYKRNAGAAGDVIGEQEK
RLWDYRMLPKKTAAFA*

>Cmac_2537.1 LYDIMamide
MRFGHIFLVVAGSLLQLSEVSSREIPQFTRQKSSTNYMLGKRSVDLGKLQKKWGLSGVVDSVSNVASDAIDSADDLKDKAIEQAANSAAAKTVTDKVADVTDTVNNEASNLDLEDAFEVADELGLPTDL
DTVIGVAGDLGIPVTQQGVIDLAQEMQIEEMMAQMPVEDLIAMGQDLAGQAVGGGGSEAATVSIEILDKLATAEAMVDNLASCKSSKDSDEGIQARKKDVKKAGGLLYDTMGKRAGGLLYDTMGKRESG
DLYNVLKKRGELDDFAKRTHLYNIMKKRAGGHLYDIMGKRAGGHLYDIMGKRAGGHLYDIMGKRAGGHLYDIMGKRDGGLLYDIMGKRDGEVAQILPSENSDVMDDLLSTGLGLATDLALPMATGGLIG
GGGGAAPDNGNLYVILDNVNELLIKLNKLQSA*

>Cmac_607.1 MxGF peptide
MQQMKNQGNLFMAGFLLLLGFAILFGQGARATEETYPVDVETRAIYDFGQPLGNLMGSPDVRRFGPVSDEEAKRMGGYSAFEKRMGGYGGLGLKKRMGGYSNFEKRMTGFGGMAKRMGGYSILKRMAGY
QI*

<u>additional *Convolutriloba macropyga* prepropeptide candidate:</u>

>Cmac_78.1 [3' missing]
MNKFLLFLAAFAVLVALSEARSKHSKRKFPTFARSDDHHHKRQGDQGTQVDDDKKDKDKDTDKGDKKGGEGGAGKKGGESGTVGDGKKGGTGGMGGDGKKGGAGGMGGDGKKGGAGGMGGDGKKGGAGG
MGGDGKKGGAGGMGGDG

>Cmac_321.1
MKSVSGVPCAKLTAVAFALFTCALILSCWTAVISADESPVEPERLRRCARDSTDCVEKAKRLVKRAIGCDLSNPYMDCFSKRNVAAKRAIGCDLSNPYMDCFSKRVPIVYESMDMAKRAIGCDLSNPYM
DCFSKRHAAPVPVVQADKRAIGCDLSNPFMDCFAKRSEPEPVKRAIGCDLSNPYMDCFSK*

>Cmac_822.1 (N-terminal pyroglutamic acid)
MKSFSAVSLFGSFLLVLLHVISTTRAQYLSPESNVGPDSVDKRSYQFLIPEKRSYQFLIPEKRSYQFLIPEKRSYQFLIPEKRTVV*

>Cmac_844.1
MSKSAVSFAPSLLVLSIAFVWTSVTSAGVGDKRSSSIGHVDGNRLQSEFYPGSETNSGQQFWKRLVSSMFAPQPQLRQSPNSRGEEEEEGGLDENGLDEFEGSKRYGEKEAPGFYQRGDENGEEEEEEE
MKRARQLEMFERSLRANSAYPVMKRGRHIMNCQRFKTFGQGYSGLC*

>Cmac_1033.1
MNYKVAAAVLVISITLLVTTLPTVEATAFYPPMDRRSRIYRFQKRSGYPPAVINNGKRLSFLQTAVSNMQTRPHWL*

>Cmac_1118.1
MNSKVLIAIFLCVSGVLAREKTPRGLPELREVKDAGLVESPVEPPVVLDTRFRAESLTKRDVEVIPISGEGRAEPSLHLQPISERAMEKRGAIAKATASEQKVL*

>Cmac_1194.1
MAFFMKSTACLQMFAIVVCLATLASSFYMPEDDFSGLDEESKRGVPPKVDPNCRDNYSRITCYHRANQCRTHTSLRMNCKQTCQVCRLPYKG*

>Cmac_2039.1
MESKFACFCIVVCLLLALYLSPVIGYEEEESPELKRRNFHAMSGYMKRGYEGNYAEEEGKRNGLGQLDTWMDKKWYRKSFPH*

>Cmac_2152.1
MKSYQQSVSFAVFTSAVVLTTFFIAVCQSISDDAKLPGPDIRAASMGARSAPGLMQARELANQMKRNWNSMRGKRENGAWMQAAALANGLNDESYSPYRMMRQMRLK*

>Cmac_2219.1 [5' missing]
RNLKKKHGVENEIQCSSFFVVFSFKSFIEFCDKVVMGSKLVVALLLSMTIVVLFQAEQSSALEKRHGFGTTGTGDSGPQGKKREMETAERAEENFKRNNHLFESLHDQ*

>Cmac_4469.1 [5' + 3' missing]
KRMDFNNEIDKRMDFNDEMKRMDFNNELKKRMDFNDGTKRMDFNDKLLKNKRMQFNDELEKRFYLTPAEIHNPRYRQQNMRWHNYDNEENTEKRAVRRTRHRRIRPKGGYRHKREEDLEPVEEEEREEP
YVSEGERHEDTKLNEKRGLSTEFLGYDDKKRSLASDVAGWSKRDPEEENQGYDTQKRSLASDVAGWSKRDPEEENQGYDTQKRSLASDVAGWSKRDPEEENQGYDT

>Cmac_7555.1
MIKFLSLNMYAFALLMTLWAFSAVKATDLEDEELDEKRGYFLNKAHKRELGDNEEEKRYLVHSFLRPSSKRAYFRNEALKGLANEEEKRDFFDPEYLDSLDKRGFFTREMLDHLGEDGKKRGYFTQEML
DRLGEEKKRGYFSREMVGSLGQDGD*

>Cmac_15344.1
MNRNLLISFSLLLLIACCNGNPNTVQEFQNDDSKRGYFLNNAMLDQDDRKRGFFLSKTRAKRVFSDENSDETENKRGFFTDKFVNNMGENYNYRRLQRNV*

<u>***Diopisthoporus gymnopharyngeus***</u>

>Dgym_c11555_g1_i2 DRFamide (FxxxFamide)
MFTLIFGGLVFCFTCITQISSHSSESNVFPVHEKQDLIDNDGDPYFVNQPEKRLAFVDQFGKRSEFVDRFGKRADFADQFVNMPRFVDKFGDNADFVDQFGKRSAFVDRFGKRAYTVDQSEKEPDFVNR
FGKKSGFVDRFGKRSDFDVSRQKKPDFVDRFGKRIDFVNQLEKKPDFVDRFG*

>Dgym_c12227_g1_i3 ASALHFamide_isoform_1
MASSETNFARCHQSSPAFQMFSLLFLASLVLSLASTCRALELMNEPELTSMESQEFIPFDSQESGEGLYYRDLDDKRASALHFGKRASALHFGKRASALHFGKRASALHFGKRASALHFGRK*

>Dgym_c12227_g1_i4 ASALHFamide_isoform_2
MASSETNFARCHQSSPAFQMFSLLFLASLVLSLASTCRALELMNEPELTSMESQEFIPFDSQESGEGLYYRDLDDKRASALHFGKRASALHFGKRASALHFGKRASALHFGRK*

>Dgym_c12147_g1_i1 AMNAARLNFamide [3' missing]
MVTNYSKNKTWICWSFVTILLFEMMYWSPVCSAHEVNDKNGKSDILNKFEKHHLTTAKIDEGIPKDSSLEDEKNIDDIGVPMKSDELNEFSKKNFPVSGNLFEKKAMNAARLNFGKKAMNAARLNFGKK
AMNAARLNFGKKA

>Dgym_c12581_g1_i2 LHFamide_2 [5' missing]
AEYMGRMPRNHSTSSHPHYLAKTRLSTLLPSLLALIFLLNGLLAGADYLYDSLDNNAMYDGGLLEPNMVNFDDAEESNYWMYYYPIENSANSIPYNKRVSPLHIGKRVSPLHFGKRVSPLHFGKRVSPL
HFGKRVSPHHIGK*

>Dgym_c12998_g1_i1_i2 FNMamide
MALRVVLPVFFFFVAVALAHYQDPSGSRLSDSFEEYPHLMEFSDEDALEKRAYAFNMGKKIAEDFTEDKKPYAFNMGKKAYAFNMGKKAYAFNMGKKAYAFNMGKKLDEEDFTQDKKSYAFNMGKRPYA
FNMGKRPYAFNMGRR*

>Dgym_c13842_g1_i5 AWD_peptide_isoform_1
MKMSIMFHLALYSLGTFIMWQISVTAAFDVEDKRGMAWDFKRAGELNELNLPLQNGYDKRGMAWDFKKRYPVDNPDDKRGMAWDFKKRDHELDDKRGMAWDFKKRDVNSEEKRGIAWDFDKRPLISTKR
GVAWDFK*

>Dgym_c13842_g1_i1_i2_i3_i4 AWD_peptide_isoform_2
MKMSIMFHLALYSLGTFIMWQISVTAAFDVEDKRGMAWDFKRAGELNELNLPLQNGYDKRGMAWDFKKRYPVDDPDDKRGMAWDFKKRDHELDDKRGMAWDFKKRDHELNDKRGMAWDFKKRDVNSEEK
RGIAWDFDKRPLISTKRGVAWDFK*
>Dgym_c14376_g1_i6 WLP_peptide
MTLIMKCTILFVFVIFHTCEVANGLSSQSLEDISGEGDLKYFSKEEPDLLYISNFGNYEDMDHLLNSFENKAKRKIGSWLPSKKSDFEDDSFSEEWNGGEAKRTIGTWLPSTKGKRSKFDSSDEIFLPE
ERSLYFNTNPVVYKRKIGAWLPNKKRAIGTWLPNKKRAIGAWLPNKKRAIGAWLPNQKRAIGTWLPN*

>Dgym_c10999_g1_i1 MIGFG_peptide [3’ missing]
MKSRFQAIIALSIFLLLPDFMKVQSYEEDRKRVVDFGTSRRSTDDLDKKRMIGFGSKRYFKDDFDERINGFRTKKWDSSEKDFDKRMIGFGTKKWDSSEKDFEKRMI

>Dgym_c12368_g1_i1 SFYNamide_partial_1 [3’ missing]
MDQQIWRKQSNCRLYRLSSAQTALVVAMLMTTLQVTHSFGTGNEDWSRVKALGGPPNEFWSSIYAGSFYDMDKSMPSFRGQSLNQDISSNKDFLTTTEDNGINHLDSMENKSSYNEKRSFYNGKRSFYD
GKRSVD

>Dgym_c12368_g1_i4 SFYNamide_partial_2 [5’missing, 3’ missing]
RRVHGGQSLNQDISSNKDFLTTTEDNGINHLDSMENKSSYNEKRSFYNGKRSFYDGKRSVDEKRSFYDGKRLFDNENSTKYNGKRSFYDGKRSFYDGKRSFYDGKRSFDDDKRSFYDGKQSFD

>Dgym_c12368_g1_i2 SFYNamide_partial_3 [5’missing, 3’ missing]
RRVHGGQSLNQDISSNKDFLTTTEDNGINHLDSMENKSSYNEKRSFYNGKRSFYDGKRSVDEKRSFYDGKRLFDNENSTKYNGKRSFYDGKRSFDDSKRSFYDGKRSFYDGKRSFDDDKRSFDDDKRSF
YDG

>Dgym_c9071_g1_i2 Insulin-like_peptide_1
MKYLQVTLSLCLLLGSLADGQTLHQLCGRELSEMLFLICEGQFYSSDPNVLMYMANISNNVDITVLNKRTTPLLKERSSTKLNHSRFKIQNNSWDDIPEMIYQLQRSLLRSTKKQRHHKQLPYQEPRLK
SVKNRVSLHNNPPPRPEDFGSKSSVLHRGKASSPPEEVSKRSITRECCTSPCSYFHLTYYCMRSYEERRNWTDAQWIGSLFQFPAMNY*

additional *Diopisthoporus gymnopharyngeus* preproneuropeptide candidates:

>Dgym_c13266_g1_i3_i4
MLSSKGIVCLLVTMALMELFYTSSAFESNQWDKPMYRAMGKRTGFHYGGTKRSPEPLRVFAKRDPLRVFSKRDDDSFEIPERRSDELDKRYD*

>Dgym_c13982_g1_i2_i5_i6 [3’ missing]
MKQPTLMPILLLSAVVTLGLLAHGVPIDWDDVEMKRTFPGDESQNEMDKKLFVYPDKKEMDKKLFVYPEKKEMDKKLFVYPEKRDEDVTHDVEKKLFVYPLESKKSMQKKLFVYPD

>Dgym_c13982_g1_i3_i4 [3’ missing]
MKQPTLMPILLLSAVVTLGLLAHGVPIDWDDVEMKRTFPGDESQNEMDKKLFVYPDKKEMDKKLFVYPEKKAMNKKLFVYPDEKEMDKKLFVY

Diopisthoporus longitubus

>Dlon_c34989_g3_i1 AWD_peptide (alternative signal peptide MKVSVLLICTAVFTITALCSSMVQS)
MKVSVLLICTAVFTITALCSSMVQSGAVKDSKRGLAWEFVKRRGDDDDMSNKRGLAWDFVKRSDMEGAQWNKRGMAWDFVKRAYSQLNDQQQRFATLERMQ*

>Dlon_c31781_g1_i2 ASALSFamide
MSWMKSRLNGQRLVILSILLSICIQMINGHQPLIYEENQANGDKSLESERASALSFGKRHYITNQGLVKRASALHFGKRMRMLDDYDKRGSEEFHFGHENGLERRASPLSFGKRVPQFERRASALSFGK
RNEDFEREANYGGRNGEFERRASALSFG*

>Dlon_c31676_g1_i2 MMGFG_peptide
MDFLKIFLIAMLGLVLLTSAGEYSGEGDESGSYERKRMMGFSRKRMMGFGNKRMMGFGAKRMMGF*

>Dlon_c8422_g1_i1 FVDxFamide (FxxxFamide)
MTTTSRTLLLHCSVLLFAAALISFTHSYPQQWLGDSLPNPLTFHQPTLVMERRDSSMNFVDGFGKRAAYGSNEAAELPKRNQPYNFVDSFGK*

>Dlon_c17292_g1_i1 PGSYH_peptide (alternative signal peptide MKQFFLCLCLALLMITHA)
MKQFFLCLCLALLMITHALYQVDAFSGEESVYLKRIPGSYHKRVPGSYHKRVPGSYHKRVPGSYHKRVPGAYHKRLVYSDEDE*

>Dlon_c29952_g1_i1 LWD_peptide [5’ missing]
TQSTWAEEFKRLWDLEKRVPEESKRLWDLEKRVPEESKRLWDLEKRLPESGLKRMWDLERRGEGGAFDTSATHSNPDRR*

>Dlon_c50571_g1_i1 FNMamide [5’ missing]
FFFFFMGKKAYAFNMGKKAYAFNMGKKLDEEDFTQDKKSYAINMGKRPYAFNMGKKT*

additional *Diopisthoporus longitubus* preproneuropeptide candidate:

>Dlon_c31386_g1_i2
MLSTRHLLIVGFFMSFAIHNSYSMWIRGSGDLDDGEAYVRRYLADPQTSASKRAWAYNLGKKSFGLADSASIDEAQKRMWSYNLGRR*

Eumecynostomum macrobursalium

>Emac_c6149_g1_i1 MRxQ_peptide
MELNGWKQWVIFLTVILGITTHLVGAHEESLSSTGLEEENNYPREGDVMRPEYFYFFDPLEESEFESEENPEDLSEVKRMRIQAMNRRSEEDEFEAEKRMRVQSLGKRTSSEYEMPLEKRMRLQSYNKR
MRVQAMGKRMRVQAMGKRVRLQSFA*

>Emac_c1841_g1_i1 SSAMHFY_peptide (SSxxxFamide)
MSNFKANLSNVIAMVSLCVVFVMITKLQTTNAISVITPQLAFGPSHEAQNDKRSSAMHFYKRGELELEPNTFQEARRRSSGLFFGKRIPILEESDEHTGDKRSSAMHFYKRQPMELSDSHNVLDSADKK
SSAMNFGKRSSAMHFYKRQADGSYLLDDNFPSVV*

>Emac_c4407_g1_i1_i2 FVDSLamide
FIHFTTAFLVLVTICSAFPFGEGNYFFGEKRGSLRNSHHFVDSLGKRSDHGDDIYYEKRGGNQHHFVDTLGKRSEDVYNSEELVNKRGMNHQFVDSLGKRANDNVDNSKLKRLLFSKPSYLQYLEQVDA
NSNESDY*

>Emac_c6146_g1_i1 PGxF_peptide
MTNFWMELTLLLSMLAIVPCLTWETAPRLSLTPAEQKADDYISTLSEEDIKEMLAEAWEESQLQEDEEPYITDTNEDDDAEMKRAPGSFTKKSPGTFVKKSPGSFVKKSPGTFVKKSPGTFVKKDPGTF
MKKDPGSFMKKSPAVFAKRFPGSFEKRSPGTFVKRSPGSFIKRSDEIFF*

>Emac_c5290_g1_i1 MxGFG_peptide_isoform_1 [5’ missing]
GYGADKRMVGFGEDKKMVGFGEDKRMVGFGEDKRMTGFGANKRMTGFGVGKRMIGFGDELDKRMTGFGTGKRMTGFGTGKRMVGFTE*

>Emac_c5290_g1_i2 MxGFG_peptide_isoform_2 [5’ missing]
NAEYMGVGFGEDKRMVGFGEDKRMTGFGANKRMTGFGVGKRMIGFGDELDKRMTGFGTGKRMTGFGTGKRMTGFGGGK*

additional *Eumecynostomum macrobursalium* preproneuropeptide candidate:

>Emac_c291_g2_i1 [5’missing, 3’ missing]
VKRNEVEERMNELKRTPEEEDFDEEEDEEDEENEDDDKRSLTRSFVDGKRSLTRSFVDGKRGLTTDFVHGKRSLTRGFVNGKRSLTRGFVNGKRGFATTHVKRYPTRTFHYGKRGFASTHVGKRPFATT
HVGGKRAFASTHVGKRPFGAISAGKRPFGAISAGKRPFGAISAGKRPFGAIS

Hofstenia miamia

>Hmia_98013351 SALHFamide

MTYPGLSFFIQIISILLIASLILTQTFAQEIDEDERSSALHFGKRIDDPMERSSALHFGKRFSSGNQDKRTTFEDRDVGLHWGKRGKRTSALHFGR*

>Hmia_98009329 MVGFG_peptide
MNSPIIFILLTVSIFLLSSAIQPNPDDAPLFQKRMVGFGTRSISDNKRMVGFGTRSQIPEKKMVGFGTKRNDDDYITKRMVGFGTRSANPEIVELPDKRMVGFGTKKRMVGFGTKKRMVGFGTRSVYPD
AFFTESDY*

>Hmia_98020157 MMGFG_peptide[3' missing]
MPTMLYIFSVFISIVFFQSISAENTDEKRMMGFGGTNHKKSSKLGDYFETQDLDDDQYNLNKKDINSELLSEELPDEDKRMMGFGSNQKRMMGFGSNQKRMMGFGSNQKRMMGF

>Hmia_98047105 AWE_peptide [3' missing] (alternative signal peptide MTSNTVCALAGTLLILTVILSLT)
MTSNTVCALAGTLLILTVILSLTKQSYELETAEFKSGNTYEENRKKRGLAWEFAKRHPWDKRGMAWEFAKRDDGGEGKRGMAWEFAKRNNDDNSKRGMAWEFAKRDAADEGKRGMAWE

additional *Hofstenia miamia* preproneuropeptide candidates:

>Hmia_98048862 [3' missing]
MQIKGCFIIYALILTLFVTAFVKAEQDNESSEDFLKDYVEKLDEDIPVFEDSINEDQDDDETKSHYKELLNTIIEHYRSMYNLPKERAIEFLRKLLSESNNESSLSKRGNSFSLGKRLDSFDLTKRPNS
FSIGKRSFLNDFQKRPNSFSIGKRSFFNDLQKRPNSFSIGKRNFL

>Hmia_98000235
MMQPCVLTATLVLVAIFHAELNEACTTNCQIQRLAKRVSQMEQDLASLAKRDAVYSAQEVDDDIENFQERRDLESDVVDDEFDNAEEKRGFNDNEENPEEDKESIKRFEDIYNIYRELKGEGQINI*

>Hmia_98008391 (N-terminal pyroglutamic acid)
MKTLPRFVFVLVALLFVSEMAAAYSGANVDKRQYLADSYDFNRIMKRLRFMPDKSFISRPVKKWETFEERK*

>Hmia_98038224 (alternative signal peptide MKAIFSSIFILYCFA)
MKAIFSSIFILYCFAASGNSMYTDEDLDVPELYERQLDDVQELATRWGKLIKQFHKREPEDDQYFESPEDEEKRAGRLIKQFNKKDDEDKRVGRLIKQFHKKDDEEKRAGRLIKNFSKRTFGTNPHEIR
YFHS*

>Hmia_98040349
MNFRILCFLCAIMILFTPKVSAIDGDATPQNDLERENRAWRSYQKRAWRSWQKRAWRSYGGAY*

>Hmia_98052542
MKPLFNHFVLFIVVISLYIIQSTARTLRTNGFGGAYAKGSFGKRGIADGVYYGRYAEGFPFDSIQ*

Isodiametra pulchra

>Ipul_2301.1 SSAMHFY_peptide (SSxxxF_peptide)
MYKVRVSVLLLVVLCLSSALAFIFETPSELEDKRSSAMHFYKRSTPLFFGKRSSAMHFYKRSGDQPVLLSEYDK*

>Ipul_6429.1 AWD_peptide
MAPTGSALMTIGGLLLLCGTGACSGEEELVLVRPVAKRAIAWDFTDSLPQRRVRNAAEQDKRIVWDFTKKVPGEDKRAMAWDFTKREAEDEPENRYLGQPDKKAMAWDFTKKAEDPEKRTLPYRFTSKK
EDSKKAMAWDFTKRAMAWDFTK*

>Ipul_9265.1 LRLQamide
MASMSGWCHAVLCLGAIIALFPTHGGSSGLDSGDQLMLGPDHIFETESTDMPQEISPEDLEKRALRLQGKRALRLQGKRGSGWEMRLQGKRLRLQGKRGDEEELGLQDKRLRLQGKRLRLQGKRLRLQG
*

>Ipul_6032.1 MRF_peptide
MSGSRDIFRIILFVSLGTLICSQAFLDEGEEDGEMDKKMRFNPGASHFDHKRMRFAPASAQMDFKRMRFAPAAAQFKRMRFAPRAESTEFEEDKKMRFAPQKKMRFAPLRSINNVEDDSTLD*

>Ipul_1005.1 LWD_peptide_01
MRASWLVLLASLQAVAFSQDTEGGSEETKRLWDYQDNKRLWDYGAKRLWDYGAKRSSEDEAKRLWDYGAKRSDDTSEDKRLWDYGVKRSEEGDEGEKRLWDYGAAAKRSEGEDKRLWDYGAKRLWDYGA
KRDNEKRLWDYKASKRVWEYKKSADDV*

>Ipul_8156.1 LWD_peptide_02
MLALLTLLCVLHTASQFVILEPIGYRVIDTKRYDPESYSLDRSRESKRLWDYSKRGITPLTPLEALTSHFTRDQLASEKRLWDYAPKRRYVPVESKRLWDYEVENKKKNK*

>Ipul_11864.1 PSFamide (FxxxFamide) [3' missing]
MMRLSVLVYLAIVFRCCFSAPNFTPLYSSQSDVPVSEEELMRMLPDVDSQQGFDLIAIAPEGEDLDDSTLEFDQPLERFIAGPPERRAFQPSFGKRFSDSSMPRRAFQPSFG

>Ipul_7403.1 PSFamide (FxxxFamide) [5' missing]
PSFGKRYISTFQPSFGKRDMSEDRRAFQPSFGKRAFQPSFGKRSLRPVILSEDDSLPGQSGIVQNRASLADFLDRHHVLIM*

>Ipul_7035.1 amidated_MIGFG_peptide [3' missing]
MLRLTLSLLLLAAFSAAQEFTSRGGQNEFPAGWKQWKRDGVDADEAEYLATQEAAEEGEELAEMYGDVDDEEDKRMIGFGQTGKRMIGFGGSGKRMIGFGGSGKRMIGFGQTGKR

>Ipul_15733.1 VRAFamide_partial_1 [3' missing]
MQPPPATLVLLTLLLSGAALSVERHYVRDVEDPLALQDDSEGLEKRDPSVRAFGKRDHSVRAFGKRTDEDLLSGQDDPESYDKRDHSVRAF

>Ipul_11839.1 VRAFamide_partial_2[5' missing]
KRDHSVRAFGKRDHSVRAFGKRDRSVRAFGKRTGGDIIWVADDPEFFDKRDHSVRAFGKRDHSVRAFGKRDHPVRAFGKRDHSVRAFGKRDHSVRAFGKRDHSVRAFGKRDRSVRAFGKRTGGDIIWVA
DDPEFFDKRDHSVRAFGKRDHSVRAFGKRDHPVRAFGKRDHSVRAFGKREMLLARE*

>Ipul_14820.1 LNFamide
MLSRIAILTSLAFVLVKSGHGRLTWTPEVWVLRSDSPVVNSFMSEDDADAFDLIPEPEQFNFQDDLSQEEDPYMAAPIYGKRSQPLNFGKRSQPLNFGRRSQPLNFGKRTQPLNFGKRSQPLNFGR*

>Ipul_3009.1 [5' missing] LHFamide
GMWEPDNAAEGVIGGTGSGTEQSVAREERRSAVEMEKRTLMGLVAAMQILALLIAPAVSGYAYEYEEVPELVRRSHTMHFGKRFPEDGARDFDGREVAAGLHKRATSSLHFGKRQ*

>Ipul_91.1 bursicon alpha like
MKILFAILLLTPVFCQDVLDGELGTKSDPFKTSQEDIDRIRKGEYDAKLILREEDGSEAKTDDINFNNCALPSEEDSNERTRCRGADSELTLGADCKYICKPYEQCSPRYWLQYYFLVNIGNYQCKPYY
KYPIVRSGCDGQCKSYSSINPFPKYYSKKFCQCCDASSFKMGYVILECWPLDWPVDIKPYSKLVWYKYPIVASCDCQQCNDFHFWEKNQEIGQDEEDAFFN

additional *Isodiametra pulchra* preproneuropeptide candidates:

>Ipul_996.1 [5' missing]
KRCVEIRHIGKRCVEIRHIGKRCVEIRHIGKRCVEIRHIGKRCVEIRHIGKECVEVRHNGKKMCVINDVMMTS*

>Ipul_12352.1 [5' missing]
RVELRHNGKKCVELRQNGKKRVELRHNGKKCVELRQNGKKRVELRHNGKKCVELRQNGKNVCDYAITVRAVCNYVITMTSWV*

>Ipul_11125.1
MRSFVTISAVLAAAILLSVSASDQFSGRRVQKSYDSIPNAFSTFDGYDAGEKRLFRPGFFSKRPFVPGAAELDEQQKRKIGCPMDDPTLPCFG*

>Ipul_3076.1 [5' + 3' missing] (no oxidation of methionine detected)
AEPEQSRMACGRSRGYSRRWKWDHASSSRHLSTRLLHSAEQLHAMQMRLVALVSLLAVCWAQHELEEPAAAEGEMGEDWDVEDLGEDKRSDFTGIPEYFYKKSIFADAAGATGKRSFFTQAAANTAAGK
KSMFGDAAGATGKKSLFGDAAEESGKRSMFAGAAQRTGKRSLGDALLDDMSEEDKKSMFANAAGRTGKKSFEPTDFKKSMFAGAAQRTGK

>Ipul_5671.1 [5' + 3' missing]
LHSLNWDNGGNCVDRNIGNICAGKPAATHPVIPEPVLQMVALNTLLAAAGGLLLLAPLARSSEDWLEERFDPSIYDLDEYDIERRVDPYMAHLNGRNIEERSVNHPMANLQNIQGRNIDEDPREDEMLS
VRMHDFGGVDMDDLHGRSMDGGLQGWEDEDERGYHPHSGKREGDGLVSAFGGYEERGLAQSMASGKRGLAQNM

>Ipul_701.1 (no oxidation of methionine detected)
MAAEMGSCGGGDWRGLRHLPSRSQPNRSQQQTAAAAARMKLSSLSLLLMVALSALFVDNASGSSIREMKRRLGCPLDDPFLACSLVKRSRDQMLPQGSMQEKRRLGCALDDPWMPCELVKRSAEETEEE
*

Meara stichopi

>Msti_3522.1 WDLamide
MARQIQIAVFVLILGIVLARVPSVDKRKMSFDLGKRKLNWDLGKRGMDLSDLSEEEYDELLDSYNGGKRAMNFDLGKRAMNFDLGKRTLNWDLGKREDDGEKRRLSWDLGK*

>Msti_6636.1 GFGN_peptide (achatin) (alternative signal peptide MRNYFVILELAILALITSA)
MRNYFVILELAILALITSAAFEEISSSDNDFLDKRGFGNKRGFGNKRGFGNKRGFGNKRSIDDGEIDTKQELVDKLVSIVMKDETIENRDERGFGNKRDYTGPLAGIGTRYSRGFGNKRGFSEPLSGIG
YRFSRGFGNKRSPPGGVKHFVIEGVCDDSSGENSDNFLLSEFVNLLNLKPGRHETENGIHQVVQGVPKCAAIGALI*

>Msti_11548.1 FRamide
MVTCKSAIKCLIFTFVFTLILISETQGRSSNDAYRSAPGGYSFRGKRLPKSVMDFRGKRLPYPGFLEFRGKKNFNSDDIDENGNDSPYSVSVPSYSSGGGKILNLN*

>Msti_5141.1_calcitonin-like_1
MVAMPTVQPVLYVMGLLLIFSCLVHHSTCNNHRKLGDQSSDENEYYWPIRDIARQIARVKTSDNSDYYWPVRDASQRLSRPVKRCTQMSTCLLNRLSQHMTSNSMKKPRYTGVKSPGK*

>Msti_7629.1_calcitonin-like_2
MYVPLTCLASLLLLATVGTSMSTRQRAILEDSAQGDSWDEFSNSPIYGPFKRLMMPLSKRCDFSTCLGDRVSSGLLGGINVGAFSPGK*

>Msti_6316.1 GnRH-/AKH-like
MKVVLRVLLIHLAICMCMATNGWTGSVNWLPGGKRSQLPVEAHDIDPELEALQAEVPDDETVTPCICSLPTNDRRRYFKSAVNYILWDSEGNPVSVVPQSGKRDD*

>Msti.22299.1 Insulin-like_peptide_1
MNTVNFLILVVVSATAICTVSAIFKKDMKWSGRLCGSKLADKWTELCDDYTSNYTMSILYLSHIRQANLANPKNVLQNRFGRSMPTILRRMRRSENINSDELVDLCCLKTCGFTTLVRFCSISEHWEKL
FEDLKEGKISAEFLDKIDQINMKSTNRI*

>Msti.rna.tri.12781.1 prokineticin like
MHGNQHRLENKFLTCIFILFSQRVASFHMEAKYQPTDDFGLKSQWGIEISSNSLLKHDVKRMMIHVECSVDDDCGAGRCCSNSRNRQIRTCKSLHGVDDFCLPHIPEWGIQYERQHVWCPCVQEAICEN
VAGRKAGRCLVKK*

additional *Meara stichopi* prepronuropeptide candidates:

>Msti_5165.1 [5’ missing]
NGRLQTGFYVNGRLQTGFYVNGRLQTGFYVNGRLQTGFYVNGRLQTGFYVDGRLHELADV*

>Msti_43027.1 [5’ missing]
RRLHHGLGRLHLYLHHGLRRLHHGLGRLHLYLHHGRRHLHHGLGRLYHGLGRLHHDQQHCLRRLDQVLKYQMFPTN*

>Msti_44607.1 [5’ missing]
RTGWRPECLRWGRTCWRIQCLRWGRTGWRPECLRWGRTCWRIQCLRWGRTGWRIQFLRWGRTGWRI*

Nemertoderma westbladi

>Nwes_ 37655.0_t1 GFGN_peptide (achatin)
MSFQGIFGLFALMGIFVVSECLRIIPSPCRNQDGTIVNRVPMVTPPQGVLQHTRTTNKGLLHAMQRRGFGNKRYPGNSLPSEDVQTVTKQSGHAVETRGFGNKRYPGDTVTMVTKRYGKEVVTRGFGNK
RFPGDYMQIARRLVGTAMPNKDDEFTPRAKENAVRMEMFGDSPGSNGNSGTRSC*

>Nwes_49866.0_t1 LRIGamide_isoform_1
MKSCIWIGVLAALVLIAYLTVTDATDEFDKRLRIGGKRHYSAENSAEHSSNSVEKRLRIGGKRDMMKRLRIGGKRSDDDSREKRLRIGGKRDNGLDKRLRIGG*

>Nwes_36484.0_t1 LRIGamide_isoform_2
MKSCIWICLMAALVLIAYLTVTDANAEFEKRLRIGGKRSHHSDESNSNDHSDSSVEKRLRIGGKRDVMKRLRIGGKRNDDSREKRLRIGGKRDNGMEKRLRIGG*

>Nwes_7201.0_t1 LRVGamide (LRIGamide) [3’ missing]
MGTSWMVVAALVVMAHIVHSWPDSSDVDKRLRVGGKRSDDFEKRLRVGGKRDDDEEMDKRLRVGG

>Nwes_60610.0_t1 AWD_peptide
MKITSFFIGSILLIITLQVCQYTSGHSIQVPGDIDGPMSLKRAMAWDFHHGGNGKRAIAWDFLKRNDENEENTGRRLNIAWDLVKQASQNEGSPGQKRMAWDFNKRNSQEKRAMGWNFLN*

>Nwes_35014.0_t1 TFFNamide (SFxNamide)
MVKLIVVAFVIIVSLTMEITSKPYESSQISAFHTNPWIELLSPEYQSLLKKCRDELDEDKRTFFNGKRTFFNGKRKRDCDFSKLFSPEYNSLMEKYGEEVDKKTFFNGRRKRNGEFNNMDK*

>Nwes_54093.0_t2_t3_t7_t8_t14_t15_t21_t24 WSFTNamide_1
MGNKIRFIYAGVILFVLVLEAIVSDAQKDVRNKLEEVELPNDAENQLVHRMKKWSFTNGKRNINSNDKRWSFTNGKRSYENADKRWSFTNGKRWSFTNGKRSVS*

>Nwes_44523.0_t2_t6_t7 WSFSNamide_2
MNRTTLHLIIALGLIIMNSLVKGDHNVGDVLYGEKGDGKRWSFGGGKRWSFGGGKRSFNDAKRWSFSNGKRNNQDETI*

>Nwes_52296.0_t8_t19_t2 LKIamide
MSALVRYICFLLCLLVTLDSIFARHWGHFVEDGGDVANDLEKRLKIGKRMYDEPYRSIDLDKRLKIGKRSSEENLSESDEDEINEDKRLKIGKRLKIGKRLKIGKRFFW*

>Nwes_37502.0_t1_t2 ELamide_1
MPAALSFTTLTILFVICHVSMATFVYELGKRGRNDDNSDDSMERRAPFVYELGKRRLQPGFTNEYTNRKRNFVYELGKRSIRSVENQMKTLGNNAGK*

>Nwes_33619.0_t1 ELamide_2
MLMRLLTITLLLFWCSLQSRARYVYELGKRGDSSEFLKSAVALDPTNKRFVYELGKRSSILKKSSPEWNEQQRRDSDEPQQRSYVFELGKRSYE*

>Nwes_32435.0_t1 LWD_peptide [3’ missing]
MKCNLVALYLAILFSTLKGVESVADDQLKEVEGSQEDGHNEAKKLWDYGASTKRHQPDVEENTEGKRLWDYGQSKRLSDNEPSKRLWDYGPAKRFVENEPSKKLWDYGPSKKLWDYGPSKKLW

>Nwes_32435.0_t2 LWD_peptide [5’ missing]
YGPSKKLWDYGPSKKLWDYGPSKKLWDYGPSKKLWVYGPSKKLSDYGPSKKLWDYGPSKKLWDYGPSKRLWDYESKKHSSNDEVN*

>Nwes_6928.0_t1 WDLGamide_partial_1 [3’ missing]
MTSCSEILNWRLLFIFVFVGYILSSVDAEVDPNDKRPIGWDLGGKRPIISKRGLNWDLGGKKRGLNWDLG

>Nwes_32008.0_t1_t2 WDLGamide_partial_2 [3’ missing]
MMLIRDRLSLLWRVLFTFAIIGFAVNADSDDSTLDNKKPIGWDLGGKRGLNWDLGGKRALNWDLGGKRGLN

>Nwes_27067.0_t1 WDLGamide_parital_3 [5’ missing]

GGKRGLNWDLGGKRGLNWDLGGKRALNWDLGGKRGLNWDLGGKKRGLNWDLGGKKKRGLNWDLGGK*

>Nwes_12264.0_t1_t2 WDLGamide_partial_4 [5’ missing]
WDLGGKRGLNWDLGGKRGLNWDLGGKKRALDRENNKRGLNWDLGGK*

>Nwes_25514.0_t1 PxFVamide_1 [3’ missing] (alternative signal peptide MDQTLMTSLLLIVILAISTVTS)
MDQTLMTSLLLIVILAISTVTSSQSGVADDKNAQAVSVSHSDQDTPRLVRRYVPQFVGKRHPPVSDAADTGDGPEADADADPELSENQLNEQELLELLSDYDFFNRFDGENHEDLDVNQDVVNGDDKSD
GVDDTLPDSDSDIDNSQEEKRYVPSFVGKRYIPNFVGKRYVP

>Nwes_14711.0_t1 PxFVamide_2 [5’ missing]
SFVGKRYIPSFVGKRYVPSFVGRKRYQPTFVGKRYVPNFVGKRYQPLFVGKRYQPTFIGKRENYEDKRYRPMFVGKRYVPSFVGKRYVPSFVGKRSTVDSTDETMTGVNSDESTPVLVEKRSAPQAETL
SSDEDNHLRHKRSLIEMEDKRTWGRPWAVRDILTQKSLEGTKRFVAPEFVGRRDSISTILSALKALQVARSEPRLATKRFQAPLFIGKRSSHEDSSLYWNKLNIDPLTESYLQELQEMQL*

>Nwes_51167.1_t1_t7_t8_t15_t20_t22_t24_t26_t27_t47 LHFamide [5’ missing]
IGRRTRTQLHIGRRTRIQLHIGRIIRTQLHMRRRTLTQLHFGRRTRTQLHTSSTTRIQPHTRWTTQYLIPTHVYQLNSRITTPVGFLRVHFEPEHPRFTPNQRP*

>Nwes_46971.0_t4 Calcitonin-like
MSKLLSLSVVVLVVALFLASTDAEHASIINREKLIKRCTTMSTCLVNRVTEGLMGNANKGRYVGPYSPGRRRRFAKQRR*

>Nwes_61081.0_t1 Glycoprotein hormone alpha
MMEIIFINLLKLVYLSILIDRCFTKEAYGCHVAHYVLSLEYPSCESQHIPLLACKGYCESFSYSSPMGNVRKQFVSQQQCCRIIESNQVEFYQRCEDGLYRGYTVNATQCHCSHCKLETLTMKNINKRS
THGRTLIHKHSNNSSKTLIDDDVDWDDRIWQHKLFTEP

>Nwes_30166.0_t2 Glycoprotein hormone alpha (alternative signal peptide MNFFENSKLLIFFPLVLFIA)
MNFFENSKLLIFFPLVLFIAENKSQEDDVDSGDGCFLTEYTMKLDFTDCETKHIHLYACRGYCVSYAYSGQQWRHSVNTPRFIAHSSCCRIYGFTRVNFNYICEDGIYTGSVPNATSCYCDNCELPEGS
QSRVTRGTQRAVRLEKNLPAPLRNATHITDKPQFQFQLTPKHNSKIFKP

>Nwes_43918.0_t3 GnRH-/AKH-like_01
MEPRVIVIVCIIAIIVDSCTAANGFSGSSNWHPSGKRNGKSSEGSNENPSSNEISGELGPCVCSGADGVRKYFKTIDEIKELSSGSGSGSSEES*

>Nwes_45035.0_t1 GnRH-/AKH-like_02
MEFRSVIILVVIVFEIGSSSAANGFTGSSNWLPSGKRGIQYNGVKGKADIGPCVCSTPDGTTKHFTSVKEIMKEGDQT*

>Nwes_49732.0_t1 GnRH-/AKH-like_03
MEFRVLVALVILVVTAIDIAKGANGFTGASNWFPGGKRNNMANLRQTGLATSMKAMANARAPKTKPETGPCVCFLPGGNLKYFESIHEVPRIEGPDID*

>Nwes_29216.0_t1 GnRH-/AKH-like_04
MSSHYTMKSYSALLLLSSVLLFIVYTTVDAETANGFTGSHNWSPGGKRTANGFTGSHNWSPGGKRNAPSGISAQEQDLQIYGPCICIAPDGSTKYVASLSDPEISEKNP*

>Nwes_64678.0_t1 Neuropeptide Y/F-like_01
MPLSSGSRLWCVSAILTGLLCLILPAGLGSATPLWSRGDQGSGGDQPVFKTPEELRRYLQDMNEYYAIVNRPRYG*

>Nwes_43226.0_t10 Neuropeptide Y/F-like_02
NTNTITMNRTVTCLLFVALALVLSVTCQPDSMLAPPGRPVEFRSPDQLRRYLKALNDYYAIVGRPRFGRSADKQTLGQAFEQQAVGNE*

>Nwes_64887.0_t1 Neuropeptide Y/F-like_03 [5’ missing]
RRVHGNSDSSSALTSSRSTSNSSSKDAAMIDVFENPFELRNYLKQLRELYAIRNRPRFGK*

>Nwes_Locus_30704.0_t1 Insulin-like_peptide_1
MYLKRFETPRWKYYLYIWTLLFSTLFQVGWQDYICGSDVPRAIVRACAAQKRSVGNYYENPLSKNNLMKSLKLKRAASGIASHCCAAGCSSGDLLAFC*

>Nwes_Locus_5627.0_t1 Insulin-like_peptide_2 (alternative signal peptide MAVYIRTRCICFCLLLLCTTMA)
MAVYIRTRCICFCLLLLCTTMAESKYICDGQQLSRAILRACAFFKRDGRSNINNQGIQDGAADFNSMEFFVKPKISQLHHKNLRSKRVGDGVASHCCQKGCSMRQLSSVC*

>Nwes_Locus_45386.0_t1 Insulin-like_peptide_3 (alternative signal peptide MFLLVAVLVSMILLQG)
MFLLVAVLVSMILLQGIPLSSQEILCGSAVPRAIVRACASQKRSSHMPYQSWQNVERRNDLIRRVYRSGMRGIASHCCSYGCSSSDLVAFC*

>Nwes_Locus_57309.0 Prokineticin like [5’missing]
TVAYSMASTCSSNLDCPQDQCCGQPVYDVLNLAKTCKPYAKQGQGCHGNILFASCDCEQGLTCVEHTGLLSFLHTDKGICRTDASTDHKTTGSAGILDLFGKK

additional *Nemertoderma westbladi* preproneuropeptide candidates:

>Nwes_37876.0_t1
MTKAWLNFGALVILAQLFPISDAFRGENWMFYKDLCQDGSIDCVLRDLPEVIMWGSDGNDQEFTAKKSLIGQRLGYEQNSPEKKIFINQGLGNDPGKRMFVNQRLGNYPGKRMFINQRLGNAPDKRMFV
SQRLIRKR*

>Nwes_21237.0_t1
MLLFAISSLMYLIIVNVVQINTDIESTITSKWNNYVHDDDGGHSQNFSNVKGDLGENVTGDFGKNVTGDVGKNVTDDVGENDTDDIGKNVYTGDIGKIVTGEVGKIVTGDVGKNVTSDVGKNVSTEFSI
TITPTDVVYLVPNICHFIWLNEPIFPFHSYLSLISAYKNLNPSRPDIRCLTL*

>Nwes_15548.0_t1 [5’ missing]
SRVYGKRGGEILIPHVYGKRGGEILFPRVYGKRGGEILIPRVYGKRGGEILLPRVYGKRGGEILIPRVYGKRGGEILLSRVYGKRTGEILIPRAYGKRAFGGRGDHFIPVVNEDKHDAAIVIPSRHGKR
GGGVFIPSAYSRPDEGE*

>Nwes_15548.0_t2 [5’missing, 3’ missing]
LCATSSVTINRLRYHNSVALSLLIFAAAVLLHSSQLGISATDLQKRGAEVYIRHVVGKRSALSIDGRDEPQQRPAANIYDTDDSLALAGYNKEAKRPAFTLPYNSKNEGKRGAEILIPRVYGKRTGEIL
LSRVYGKRGGEILFPRVYGKRGGEIL

Sterreria sp.

>Sterr_c21028_g1_i1 WDLamide_isoform_1 (alternative signal peptide MKAFHQCLPAVLLFAAIGTQVA)
MKAFHQCLPAVLLFAAIGTQVAHCEFELSQTNEKRPMTWDLGKRPISWDLGKRSDEKRPMSWDLGKRSDEKRPMSWDLGKRPMSWDLGKRSDSAESDKDFEELIAKKPMSWDLGKRSSDKRRR*

>Sterr_c21028_g1_i2 WDLamide_isoform_2 [3’ missing] (alternative signal peptide MKAFHQCLPAVLLFAAIGTQVA)
MKAFHQCLPAVLLFAAIGTQVAHCEFELSQTNEKRPMTWDLGKRPISWDLGKRSDEKRPMSWDLGKRPMSWDLGKRSDEKRPMSWDLGKRSDDKRPMSWDLGKRSD

>Sterr_c21856_g1_i1 LRIGamide [5’ missing]
YQRRVRAEESGDIDKRLRIGGKRAYDEKRLRIGGKRSDDDELEAIKRLRIGGKRAYDEKRLRIGQEKRRRRV*

>Sterr_c16337_g1_i1 ELamide [5’ missing]
INAEYGDGQNENSDQFADKRMRHEWWELGKRLRPEWWELGKRSMQPEWWELGKRLRPEWWELGKRAPRHEWWELGKRLRPEWWELGKRSKDGEFESS*

>Sterr_c22814_g1_i1 Calcitonin-like_1
MGSRSSSPWQLVSVIVVILLISSSSSSFIMRRFDTLEGNEEQLNSLQEMARRSDVIPLHIAYKRFMSEAKRCTNSPTCLLNRVTEGLGSRYRNYPRYTGAFSPGKK*

>Sterr_c17081_g1_i2 Calcitonin-like_2
MNLLVPLLIVSIALSITDGRNILRSEMTKTMKVKNPLAKNLFAKRCEGLGTCLLNRVTSGMLGKGPSVGAWSPGRK*

>Sterr_c4877_g1_i1 GnRH-/AKH-like_01
QRRVRGFALSLLVVVMAIDMTLAANGFTGSTSWLPGGKKRSPSFSVDDSAPFSEENAQPAQLGPCVCLHPDGSRRYFKTVVNYVLLNKDGEEISAKFDEEPNKKERRFF*

>Sterr_c566_g1_i1 GnRH-/AKH-like_02 [5’ missing]
QRRVRGSTSWLPGGKKRSQEPVVTDTKEQAVLGPCCVLNQMARDVTLKRLLTTYSGTKMTNLWLPRKYHNLKTNLFSRQTPVVTQSL*

Xenoturbella bocki

>Xboc_12867.1 SFWNamide
MNYYIYPLFLAILLWYQLPLTASEETLADYMKEDGTTDSGIGIRSFWNGKRAWADQGLDEMINEEARAFWNGKRSFWNGKRSFWNGKRAPVETDFDEDKRSFWNGKREPDVGENYDDALLKKSFWNGKR
SFWNGKRSFWNGKRADDSQREDIPPVEYMELFDRLFGHQSDGKLAP*

>Xboc_21216.1 PxLFVamide
MTNMAIISVCVLLVLAVNIVNGSADFCEQFPDLCDDAEMSKRQLNVFPWYEVWNSGKRQDVEIRREPPLFVGKRREPPLFVGKREEASYFVGEKK*

>Xboc_836.1 LRFDIamide (alternative signal peptide MKLFDLFCVTLVAGIASVYCDA)
MKLFDLFCVTLVAGIASVYCDAEEGFMPASSDVDKRLKFDIGKKRHFDDKRLRFDIGRKRAWEEGQENDYAQELVLGMADGVHDYLANNADDSVSKRLYDMSKRLRFDIGKRLKFDIGKRLGADESNDL
VVIGGVEIPVCAQEDEPGLSLCGFAPMGGRWWPICSDNCEELKADSYD*

>Xboc_2558.1 GFGN_peptide (achatin)
MSCTSVTVCYWLLMCVLMCATVLSTPVGELGVYDDADLKDNLGDISDTSAQETARLVSSCLSYVSELMRSDVDNALMLDIDDRGFGNKRIAGFGNKRIPGFGNKREPGFGNKRGFGN*

>Xboc_1521.1 SLQFamide [5’ missing]
GRRSLQFGRRSLQFGRRSLQFGRRSLQFGRRSLQFGRRSLQFRHLSMSHARRFPNTQQGRR*

>Xboc_4670.1 Calcitonin-like (alternative signal peptide MNNKTLYIVLSMVLSTMLVLASS)
MNNKTLYIVLSMVLSTMLVLASSAPTDANSRKKRDLESDLAAVKFIEALLNLEDNVERISYEKELYEQPMPINMESNPVRRTDCSMSVCLQSQIAHALLSRPKGPDTGANSPGK*

>Xboc_4807.1 Glycoprotein hormone beta
MKSTFAFLILIVCSFAAEGLATTTELDDLVMCVKREYRQHIASMSGCRDERILTVACWGRCETQMVPKLEPPYKESFHSVCIPYNYTIGQIQMQDCDEGVDPTYSFPQPGICMCQSCADQGYVVACH

>Xboc_39242.1_ Bursicon beta
MLTLLLLTLAVGWGAATEGTAETCHVVFSDTTIRQQVDYGAEGQAVCTGTVTLHRCEGNCYSQARPSVLHGFASSCNCCRETVLVETEVVLLDCFDGAGDGDHLPDVRYTLRIQEPVECACSRCYN

>Xboc_26735.1_ Glycoprotein hormone alpha (alternative signal peptide MHHRRTLVIVVTLVVVVHCICPILT)
MHHRRTLVIVVTLVVVVHCICPILTFPTLTNGLSPPMDDHTRLGGPLYFRGFHNGSRAVGEEVDSRILNVKIRENKARSKRSGIVGCHLVGYIQRVEIDGCTPVNVAMNACRGYCVSYAYPTNPGGPYL
FTASTQCCRITERHRVPFIVECDNGGKYQGFFLSARACACGICDYES

>Xboc_6444.1 GnRH-/AKH-like
MFTIYSQAMPRMDRRAMMAIGLVLMLVVQSCLAANGFTGSSNWLPAGKRSFMDQETPAPDDVEENGGEKTIGPCVCAVLDNGRRYFKTVINYMLWDEKQAHNDRMQANRRDLLDDKLWL*

>Xboc_7489.1 Vasotocin
MYRTVFIYTLVTVLSLYADVASSCLVQGCPIGGKRSMNDAERQCSACGPGYRGVCVGLQTCCGDFGCHMGTDDAKMCLTEQINPEPCHVEGRKCGLNAYAKCVADGICCDFETCTLDEKCQQIGEGHDS
WPANNNDAGVGRITAFLRSLRADQ*

>Xboc.20871.1 Insulin-like_peptide_1 [3’ missing]
MGIVTMVCMSLLVLLAVIGGSDAVNRHLCGAELANTLRMLCGDRGYNAPQYEGAHGVMSHSHYTIPVFRTKRAAHNYLGAVVPNRMKRGTGRIVQECCRQTCSLSNLALYCAPERLPIDISSENSEESF
EFLETSVDTTSAESATDGVEEGEYSSGDSELNEVEVIDNDGTNMIAYR

>Xboc.rna.tri.22210.1_ Prokineticin like
MLGERCVGFRGIVLTTCVVYTVVVLVTVTSAYHGLSLNTADEPPFSEDSLYTNQGIVLDQVRKRSKTSSRTRHEVYNVFRRAPPVEVCYKDADCRPHGCCVRSHYIPTINQCRPLAGAGQKCAPPDLFI
RGLRDTDYCPCTASVTCVKVNRKDSFGYCLA*

>Xboc.rna.tri.35664.1_ Prokineticin like [5’missing, 3’missing]
FGGVFTREDRFPKTIQRCSEDVDCPVSHCCAYSLFAQLKECKPLGSEGDTCNVFSFPYAYDGDRQGRLCPCRRHLLCN

additional *Xenoturbella bocki* prepronueropeptide candidates:

>Xboc_4088.1 [5’ missing]
SVGSGRRSVGSGRRSVGSGRRSVGSGRRSVGLSRRPVGSMGSIGSGRLPAGSGRLPVASPVSGP*

>Xboc_5920.1 [5’ missing]
GRGERGGSVYGRGERGGPVEGRGERGGSVYGRGERGGPVEGRGERGGSVYGRGERGGPVERRGERSGSVEEIGERGGSVEGRGGSCGSVDVSITGSVTALSRSALSVASTLWASLARA*

>Xboc_1407.1 [5’ missing]
LLVARVGRGGLLVARVGRGGLLVARVGRGGLLVARVGRGGLLVARVGRGGLLVARVSRGGLELYKVVQLSSDSQNRSHLRSKFIADDDVAVS*

Xenoturbella profunda

>Xpro_16203_g1_i1 SFWNamide
MNYQCCPLLLSLLLWHHFPSTASEATLADFLKHDGTTDSGGIGVRSFWNGKRAWTDDGLGEPIETMARSFWNGKRSFWNGKRAALEVQPDFDERSFWNGKRDPALQQQQTVDELFDDHHSKKSFWNGKR
SFWNGKRATDVGNGVVPPAEYLELFNELFGHQSNGKLAA*

>Xpro_12884.1_i1_i2 LRFDIamide (alternative signal peptide MKLFDIFCFALFAGVASVYCDA)
MKLFDIFCFALFAGVASVYCDAEDSFIPASSDVDKRLRFDIGKKRHFDDKRLRFDIGRKRAWEGTDGGDYAQELVLGLADEGHEFDDETINKRLMYDINKRLRFDIGKRLRFDIGKRLGADHLKETTDT
LIIGGVEIPVCAQEGNAGMSLCGFVPLGGRWWPICSDDCEELNEENSYD*

>Xpro_21851.1_i1 GFGN_peptide (achatin)
MAATSVYMTCCVLMAVLLAFTTVMCDPITNDLGVGLYDDENIQDFQEDSQLGSEARASLVSTCLGYVSDLMRQDLHSEMFDIQTRGFGNKRTPGFGNKREPGFGNKRDPGFGNKRGFGN*

>Xpro_9341.1_i4 Glycoprotein hormone beta
MRPWLRMMIVCLSVAVSLATTTDLENLFKCVKREYRQHIASKIGCRDERVPTIACWGRCETQMFPKLEPPYKESFHSVCIPYNYTVGHVEMQDCDEGVDPIYTYPQPGICMCQRCGEQDYTVACH

>Xpro_1530.1_i1 [5’ missing] Glycoprotein hormone alpha
EVDSRILNIKIRETESRLKRTRSSVGCHLVGYMHSVQIDGCTPVNVAMNACRGYCVSYAYPTNPGGPYLFTAATQCCRITERHRVPFIVQCVNGEKYMGYFLSARACACGICDS

>Xpro_1888.1_i2 GnRH-/AKH-like
MFTIYNRGMTGMDQRAMMLVIGITMVLVVQTCFAANGFTGSSNWLPGGKRSYTDRGTPKPDEIDENAGEKTIGPCVCAVQDNGRRYFKTVINYMLWDEKQAQNERVQADRRDLLNDKLWL*

>Xpro_ 9608.1_i1 Vasotocin
MDRMLFVCVLVTVFSLCTNSVSACLVQGCPIGGKRSLNNADRQCSACGPGYRGVCVGVQTCCGDFGCHMGTEDAEMCLTEQNNPEPCQLKGKKCGLNAFSKCVADGICCDIETCTLDDDCLHVTNTNYN
SWATGEGGVDTIKKFLRSLRGDQ*

>Xpro_3311.1_i1 Insulin-like_peptide_1
MLMKKPVSVFVTCLIINQLLFTNSVNALRKCGSHLVSTLHMVCHGRYNKRWGPVELDRYSALDFLGSYPLHPAAKRQVVSECCYKSCTMSELTTYCQH*

>Xpro_13741.1_i Insulin-like_peptide_2

MDTVNMVCVSLLLLVFAMTVSVEGGNRHLCGAELANTLRMLCGDRGYNTPQYGGAHGIMSHRHYSLPVFRSKRAAHNYLGALVPHRMKRGTGRIVQECCRQTCSLSNLALYCAPQRRPIDISSEESGEE
NFQFLDDTNQDDTSSSYNGGDSTAEGIATSSSVGSDETGAEYEFSGDDTDAMMTEDVEVLDNDGSNLIAYRRRRRRRRRDMAELSAEYGFGDGLEGAETTSERRVLWGKWHGLPVRQQATLESEEDEDV
ESPDWIRFGLPR*

additional *Xenoturbella profunda* prepronneuropeptide candidates:

>Xpro_20770.1_i1 [5’ missing]
NLGLFGRKTKCRHIFGRIAKFRPLFGRKPKFRPLFGRKTKFRPLR*

>Xpro_14971.1_i1_i2_i3 [5’ missing]
GGGRSLPGESGGGRSLAIESGGRRSHTVESGGWRSLAVEAGGRRSHTVESGGWRSLAVEAGGGRSLDGRAAVCIIGASYSSCPSPADG*

>Xpro_14077.1_i1 [5’ missing]
GYRTEMSGYRRGKPGYRTEMSGYRRGKPGYRTEMSGYRRGKPGYRTEMSGYRRGKLGYRDIGEGGQDIGISDRNVKTLKMTLKSYHVSKREKAALLLPVILINKKYKAE*

Reference sequences

Signal peptide **basic cleavage site** **C-terminal amidation + cleavage site** **Predicted active ligand** **Cysteine**

Human oxytocin shown in Figure 2a:
>NP_000906.1 oxytocin-neurophysin 1 preproprotein [Homo sapiens]
MAGPSLACCLLGLLALTSACYIQNCPLGGKRAAPDLDVRKCLPCGPGGKGRCFGPNICCAEELGCFVGTAEALRCQEENYLPSPCQSGQKACGSGGRCAVLGLCCSPDGCHADPACDAEATFSQR

Neuropeptide Y/F shown in Figure 2b:
>ADC84429.1 neuropeptide Y prohormone 1 [Schmidtea mediterranea]
MTFIYGFLCLTLVNVICSQKSLFIEPPAKPEFFDDPELLRNYIKKLNEYFAIVGRPRFGKRFDRGFS
>sp|P01303.1|NPY_HUMAN Pro-neuropeptide Y
MLGNKRLGLSGLTLALSLLVCLGALAEAYPSKPDNPGEDAPAEDMARYYSALRHYINLITRQRYGKRSSPETLISDLLMRESTENVPRTRLEDPAMW

Human calcitonin shown in Figure 2c:
>sp|P01258.2|CALC_HUMAN Calcitonin Precursor
MGFQKFSPFLALSILVLLQAGSLHAAPFRSALESSPADPATLSEDEARLLLAALVQNYVQMKASELEQEQEREGSSLDSPRSKRCGNLSTCMLGTYTQDFNKFHTFPQTAIGVGAPGKKRDMSSDLERD
HRPHVSMPQNAN

GnRH and corazonin shown in Figure 2d:
>NP_001076580.1 progonadoliberin-1 isoform 2 [Homo sapiens]
MKPIQKLLAGLILLTWCVEGCSSQHWSYGLRPGGKRDAENLIDSFQEIVKEVGQLAETQRFECTTHQPRSPLRDLKGALESLIEEETGQKKI
>sp|Q5DW47|CORZ_APIME Pro-corazonin [Apis mellifera]
MVNSQILILFILSLTITIVMCQTFTYSHGWTNGKRSTSLEELANRNAIQSDNVFANCELQKLRLLLQGNINNQLFQTPCELLNFPKRSFSENMINDHRQPAPTNNNY

Achatins shown in Figure 2e:

>XP_014662331.1 [Priapulus caudatus]
MAGMYGPWTMVGSRTAVIVALLSAAVVAQQPNQRDFEAFLDRATLADELTQRDERGFGNKRNLVAVDGDATLPEYAAAAGYAANDVTAEKRGFGNKRSLPLDAEDVNKRGFGNKRVLESGWYGDDDDDD
GVLGVVPALTLATQPRLLHESQLEFVDDSGASEQFEKRGFGNKRGFGNKRGFGNKRGFGNKRGFGNKRGFGNKRGFGNKRGFGNKRGFGNKRGFGN*

>XP_002732147.1 [Saccoglossus kowalevskii]
MASSLLHRIILFLLVSTFLKVRTESSESSPNLHIVGNIQLTELADQGDDALIEIDENEVKRGFGNKREDVVFADDVKRGFGNKRDGFQTILDDEKRGFGNKRAEPEKIYGNTIFGVASLKDLEEDEGRK
RGFGNKRDFENSKVSDSDSESVDTIADISELKYGLENKRGFGNKRGSSSMELIDDKRGFGNKRVDTLTELQADDDDKRGFGNKRGFGNKRVDTFEEFQVDDDDKRGFGNKRGFGNKRGFGNKRVDTFEE
FQVDDDDKRGFGNKRGFGNKRGFGNKRGFGNKRGFGNKRTETAGYEPDSPLKGFIDKWRKMEEEAVSDRLSEKKNS*

>AQS80481.1 [Charonia tritonis]
MTSYYRYLLTLAIVIAAVKLLLADDLDFNDDASFALGEDFEPFGDIDFGKRGFGNKRGFGDKRGFGDKRGFADKRGFGDKRGFADKRGFGDKRGRSYSPSNLMAALFRSYYHRQPLAGSIALKRLLEKQ
GIWQ

>PAA66935.1 [Macrostomum lignano]
MTAQLVRLTWLIAVATIAACAILCREASGTTMPAASPNLHDWEEQAGVDTELDFNKRGFINKRSFINKRGFINKRGFINKRGFINKRGFINKRGFINKRGFINKRGFINKRGFINKRGFINKRGFINKR
GFINKRGFI
NKRGFINKRGFINKRGFINKRDYFKKRGFFDKRQDDLLFPMKVRMCVAYGLFCDRIHVWPFQQG

>XP_015908358.1 [Parasteatoda tepidariorum]
MNKLRDLILTVIAVLEIQLICCGAEESELQEVREGLYPSTMAYLKGANHQQPEYSWRNYNSPDEENVSSDDVRNDEETEKRGFGEKRGFGEKRGFGEKRGFGEKRGFGEKRGFGEKRGFGEKRGFGEKR
GFGEKRGFGEKRGFGEKRGFGEKRTPQHHAQSNHFISFVTQWVRILKPEDLEKYKHLSRGLSD*

Achatins mentioned in text:

>Locus_56724.0 assembled from SRX1343820 [Halicryptus spinulosus]
MFRLYTFMLLFLFTLLLVHSCLAFEAEVESMPLLLKSIVANEAKTEMYNPSPIDELANEEYREDLVELENEGFDNNQEYDNEIRLEERGFGNKRGFGNKRGFGNKRERSNVLENHFQEFTKRGFGNKRA
DIENKRGFGNKRGFGNKRGFGNKRIGVSSAAFRLAKLTNVRDIFDELAKSDNEKRGFGNKRGFGNKRGFGNK*

>XP_002598112.1 [Branchiostoma floridae]
MTSLEGGVSPGTRPRSGHVGPQPPCQAALRVRMLLLQVLLLGCVALHLSESSPVQTLYEPPFLSSDHHGNDISGADDTFAADFGQSTTEPDDLGIGFQTRGFGKKEAWAPYAHALSARGFGNKRGMGNK
RGFGNKRAAEDERYAAVQELVERLRHQAEREGLADLMRYGSQAEGGDEADVTDAKRGFGNKRASRLAR

Echinoderm SALMFamides shown in Figure 5a:

>ALJ99974.1 L-type SALMFamide [Asterias rubens]
MKGQHLLAVAVVVVAGSFGIIEAYSPFGGYNRAPFDNVWVRADSMARGGSTGEDEANEQRMTGAKRPAGASAFHSALSYGKRGDDDSAEVERRAYHSALPFGKRTPIEKRAYHTGLPFGKRDDEAAEQD
AMMERRGFNSALMFGKRLHSALPFGKRGYHSALPFGKRLDTTDEGDIIERRGYHSGLPFGKRATDDEAVNDILDQLRSEEN

>L-type SALMFamide [Patiria minata] - from Elphick et al. 2015: Reconstructing SALMFamide Neuropeptide Precursor Evolution in the Phylum Echinodermata: Ophiuroid and Crinoid Sequence Data Provide New Insights. Frontiers in Endocrinology, 6.
MKLYPLLAVAVLFVAGPFRIIEAYSPFGGYHRALLGNVWVRASDNRARPASTPEEEANEQRMTGAKRPAGSPVFHSALTYGKRADEADTDAAVERRAFHSALPFGKRTAMDRRGLHSALPFGKRDDEEA
EQDALMERRGFNSALMFGKRIHTALPFGKRGYHSALPFGKRSDEEEGTAMERRGYHTGLPFGKRDDGTDAAVSEILSQLRSED

Mollusc PxFVamides shown in Figure 5b:

>AQS80535.1 PXFVamide-1 [Charonia tritonis]
…SVLAHNTGTDANSSQLDSKGHVASSAVTSPSPSGGSNGAADNAESTASAPSNPLPPQHPDLNPPFVISESGESLVPAASSLTYQLPMTVLLARAPLQAPHRPYVTLLGPGDEEYAASLIQEPQANKVQ
DNEITEHREPEDIEAIDQVGSNDNEVTDDFDQTDDKLSDEEHPNQKLRVKRGGYTAPYFVGKRSLEESEEIEQEELQKRDGDDYGSDEQKEAENQEDDAAEKRASPMFIGKRRTPMFVGKRGPAPMFVG
KRRTPMFVGKRRTPMFVGKRGASMFVGKRGPTPMFVGKRRTPMFVGKRRTPMFVGKRGPAPMFVGKRRTPMFVGKRRTPMFVGKRRTPMFVGKRRTPMFVGKRRTPMFVGKRRTPMFVGKREDDDFAGY
TLTEGGADPVIDGQPASMFDEKRAPAPMFVGKRAPAPMFVGKRRTP

>pfu_aug1.0_10475.1_31931.t1 [Pinctata fucata] – from Steward et al. 2014: Neuropeptides encoded by the genomes of the Akoya pearl oyster Pinctata fucata and Pacific oyster Crassostrea gigas: a bioinformatic and peptidomic survey. BMC Genomics, 15.
…DIDPALDEFEGSEGEIYKRSHKPIYVGKKSYDEPKDDVSNIDGAYASDLDTLDNDLRYLIQHSDAFQRDTRYKPRIVGRSDENPMDGEWMEKRRSPLFVGKRRAPIFVGKRRMHLIVGRGLDRAPKFV
GRRRSPLFVGRRSNGRNYRDPVFSYVVMRRSVSSSGNAAPFSQASSAQSLLLALDDQSLADKSRQGRYIHPTAQAQGHVAQFALPQHKRFVSPTFIGKRTDEPNNLDSYDPDNSGFDIDHQPMVVHKRF
EVPMFIGKRYSDTIENRENWNAEHSGAINWACSKYEDFIVRVKRLASKLLVQGYLAQRLKSSMRKFFGRYGDIIKAYQISLSRMVSDILNFD

Insect Allatostatin A's shown in Figure 5c:

>XP_003425678.1 [Nasonia_vitripenis]
MSSSNLSGTAMSLTIFCVLSILGGTSVAMDEQPSASSSSGVSSSSSSSSHGPHFSPLVDPREQIVGGKRAYTYRSEYKRLPIYQFGLGKRWVDDKRSQPFSFGLGKRTRPYSFGLGKRSSYSEDDDSRY
GLDLSYLIQPSDLYEQLAQRDALENYLQQQQAIKRTGGFNFGLGKRDAEMNEGMMREDGLHEKVPVKHSRDKYLFGLGKRFYEPATMQDDEDEEMLEDA

>NP_001037036 [Bombyx mori]
MLSACLPLLLVLGAALAAEQVAEHGAEQVAEHAAPLEKRSPQYDFGLGKRAYSYVSEYKRLPVYNFGLGKRSRPYLFGLGKRSAGAEQLDDDISNEADQNTLDELFDQYDDSAAVPTGYVEKRARPYSF
GLGKRFAEEPAEDKRARMYSFGLGKRARSYSFGLGKRLSSKFNFGLGKRQRDMHRFSFGLGKRSEDDTSENYIDA
