# Supplementary material for: Xenacoelomorph Neuropeptidomes Reveal a Major Expansion of Neuropeptide Systems during Early Bilaterian Evolution
Source: Mol Biol Evol. 2018 Aug 24;35(10):2528–43. doi: 10.1093/molbev/msy160 (PMC6188537; doi:10.1093/molbev/msy160)
Supplement: Supplementary Data [file msy160_supp.zip › Supplementary_Tables.pdf]

Supplementary Table 1: Distribution of plesiomorphic bilaterian neuropeptides and neuropeptide receptors in xenacoelomorphs.

	Xboc	Xpro	Asc	Nwes	Msti	Ste	Dgym	Dlon	Hmia	Ipul	Emac	Csub	Cmac
Bursicon/GlyHo*										(P)			
Bursicon B*	P												
GlyHo A*	P	P		2xP									
GlyHo B*	P	P											
ILP*	P	2xP	23xP	3xP	P		P						
Prokineticin	2xP			R P	P								
Achatin	P	P		R P	R P			R					
Vasotocin	R P	R P	R P										
GnRH	R P	R P	P	4xP	P	P							
NpY/F				R1,2 3xP	R1								
t-FMRFa	R			R	3xR		R						
Tachykinin	R			R2			R1,2	R1,2	2x R2	R1		R2	R1,2
Luqin	R			R					R				
Allatotropin	R			R	R		3xR	2xR	R				
Leucokinin	R			R									
Sulfakinin	2xR			3xR				R					
TRH				R1,2	R1,2								
ETH				R	R								
CCHa					R								
NpS										R		R	R
e-FMRFa/MIP							R						
Asta-A	R			R					2xR			R	R
MCH			R	2xR	R		R		3xR				R
Calcitonin	2xR P	3xR	P	2xR P	R 2xP	2xP							
CRF	R	2xR		2xR	R								
PDF	R			4xR	R								
PTH	R												

R = receptor, P = Peptide. A number before "R/P" indicates number of similar sequences, while a number behind "R" indicates separated groups of xenacoelomorph sequences in this bilaterian receptor group. Brackets indicate that this sequence shows great divergence. The asterisks indicates that we only tested for the presence of peptides, but not receptors (as these receptors are also known from non-bilaterian).

Supplementary Table 2: Distribution of novel neuropeptides in xenacoelomorphs and number of detected precursors.

	Xboc	Xpro	Asc	Nwes	Msti	Ste	Dgym	Dlon	Hmia	Ipul	Emac	Csub	Cmac
SFxNa	1	1	1	3			1					1	
LxFa	1			1			3	1	1	2		1	
PxFVa	1			2									
AWDF				1			1	1	1*	1		1	1*
LWDY				1				1		2		1	1*
FxxxFa			1				1	1		1*		1	1
FNMa							1	1				1	
MRF										1	1	1	
SSxxxF										1*	1		3*
MxGFG							1	1	2*	1*	1		1*
LRIGa			1	3		1							
ELa			1	2		1							
WDLa			1	1	1	1							
LRFDIa	1	1											

The asterisks indicates that at least one of the processed peptides from this precursors was detected by mass spectrometry.

Supplementary Table 3: Affinity of xenacoelomorph GPCRs to protostome and deuterostome sequences of the corresponding receptor types in the RAxML and FastTree sequence analysis.

**Receptors**	**RAxML**	**FastTree**
Prokineticin	Deuterostome	Deuterostome
Np-F/Np-Y/PRP 1	Protostome (Np-F)	Protostome (Np-F)
Np-F/Np-Y/PRP 2	Deuterostome (PRP)	Deuterostome (PRP)
Leucokinin	Both	Both
Luqin	Deuterostome	Deuterostome
Tachykinin 1	Both	Both
Tachykinin 2	Deuterostome	Deuterostome
Tachykinin 3	Deuterostome	Both
t-FMRFamide 1	Both	Both
t-FMRFamide 2	Deuterstome	Deuterostome
t-FMRFamide 3	Protostome	Protostome
Asta-A/Galanin	Protostome	Both
Asta-C/MCH 1	Both	Deuterostome
Asta-C/MCH 2	Deuterostome	Deuterostome
GGN-EP/GRP	Deuterostome	Deuterostome
ETH	Both	Both
TRH 1	Both	Both
TRH 2	Protostome	Protostome
Sulfakinin/CCK 1	Both	Both
Sulfakinin/CCK 2	Deuterostome	Deuterostome
GnRH	Both	Both
Achatin 1	Both	Both
Achatin 2	Deuterostome	Deuterostome
CCAP/Np-S	Deuterostome	Deuterostome
Vasotocin 1	Both	Deuterostome
Vasotocin 2	Both	Both to vasotocin/CCAP/Achatin/GnRH
Allatotropin/Orexin	Deuterostome	Deuterostome
DH31/Calcitonin	Both	Deuterostome
PDF 1	Deuterostome	Deuterostome
PDF 2	Deuterostome	Deuterostome
DH44/CRF	Deuterostome	Deuterostome
PTH	Deuterostome	Both

The sequence affinities reflect the previous controversies of the phylogenetic placement of Xenacoelomorpha. However, these affinities are based on our gene trees (Figure 1a and 1b and Supplementary Figure 3a and 3b) and not on species trees. Therefore, they are not appropriate to determine any phylogenetic relationships. The evaluation is simply based on whether the most closely related sequence is a deuterostome or protostome sequences and do not reflect any support by bootstrap values or SH-like support values. “Both” indicates a similar affinity to protostome and deuterostome sequences. If the xenacoelomorph sequences group in more than one cluster within the same receptor type, these clusters are indicated by numbers.

Supplementary Table 4: Species and accession numbers of sequencing data and tissues/stages of original RNA source.

Species	Accession No.	RNA source	Animal clade
Childia submaculatum	SRX1534054	Several complete adults	Acoela
Convolutriloba macropyga	SRX1343815	Several complete embryos and hatchlings	Acoela
Diopisthoporus gymnopharyngeus	SRX1534055	Several complete adults	Acoela
Diopisthoporus longitubus	SRX1534056	Several complete adults	Acoela
Eumecynostomum macrobursalium	SRX1534057	Several complete adults	Acoela
Hofstenia miamia	PRJNA241459	Several regenerating and developmental stages	Acoela
Isodiametra pulchra	SRX1343817	Several complete embryos and adults	Acoela
Ascoparia sp.	SRX1343822	Several complete adults	Nemertodermatida
Meara stichopi	SRX1343814	Several complete embryos and adults	Nemertodermatida
Nemertoderma westbladi	SRX1343819	Several complete adults	Nemertodermatida
Sterreria sp.	SRX1343821	Several complete adults	Nemertodermatida
Xenoturbella bocki	SRX1343818	Single complete adult	Xenoturbella
Xenoturbella profunda	SRP064117	Body wall of a single specimen	Xenoturbella

Supplementary Table 5: Species and accession number of sequencing data or origin of genomes.

Species	Accession No. / link	Animal clade
Astrotoma agssizii	SRR1695485	Echinodermata
Labidiaster annulatus	SRR1695480, SRR1695481	Echinodermata
Leptosynapta clarki	SRR1695478	Echinodermata
Acanthaster planci	OIST Marine Genomics Unit, Great Barrier Reef COTS Assembly; http://marinegenomics.oist.jp/cots/viewer/download?project_id=46	Echinodermata
Saccoglossus mereschkowskii	SRR1695461	Hemichordata
Ptychodera flava	OIST Molecular Genetics Unit; https://groups.oist.jp/molgenu/hemichordate-genomes	Hemichordata
